# USP10/GSK3β-mediated inhibition of PTEN drives resistance to PI3K inhibitors in breast cancer

**DOI:** 10.1172/JCI180927

**Published:** 2025-09-23

**Authors:** Nishi Kumari, Sarah C.E. Wright, Christopher M. Witham, Laia Monserrat, Marta Palafox, John L.C. Richard, Carlotta Costa, Moshe Elkabets, Mark Agostino, Theresa Klemm, Melissa Eccles, Alex Garnham, Ting Wu, Jonas A. Nilsson, Nikita Walz, Veena Venugopal, Anthony Cerra, Natali Vasilevski, Stephanie Bridgeman, Sona Bassi, Azad Saei, Moutaz Helal, Philipp Neundorf, Angela Riedel, Mathias Rosenfeldt, Jespal Gill, Nikolett Pahor, Oliver Hartmann, Jacky Chung, Sachdev S. Sidhu, Nina Moderau, Sudhakar Jha, Jordi Rodon, Markus E. Diefenbacher, David Komander, Violeta Serra, Pieter Johan Adam Eichhorn

**Affiliations:** 1 Cancer Science Institute of Singapore, National University of Singapore, Singapore.; 2 Curtin Medical School, Faculty of Health Sciences and; 3 Curtin Medical Research Institute, Curtin University, Bentley, Western Australia, Australia.; 4 Vall d’Hebron Institute of Oncology, Barcelona, Spain.; 5 Massachusetts General Hospital Cancer Center and Harvard Medical School, Boston, Massachusetts, USA.; 6 The Shraga Segal Department of Microbiology, Immunology, and Genetics, Ben-Gurion University of the Negev, Beer-Sheva, Israel.; 7 The Walter and Eliza Hall Institute of Medical Research, Melbourne, Victoria, Australia.; 8 Department of Medical Biology, The University of Melbourne, Parkville, Victoria, Australia.; 9 Harry Perkins Institute of Medical Research, University of Western Australia, Perth, Western Australia, Australia.; 10 Department of Pharmacology, Yong Loo Lin School of Medicine National University of Singapore, Singapore.; 11 Genome Institute of Singapore, A*STAR, Singapore, Singapore.; 12 Mildred Scheel Early Career Centre, University Hospital Wuerzburg, Wuerzburg, Germany.; 13 Institute for Pathology, University of Wuerzburg, Wuerzburg, Germany.; 14 Anatomical Pathology, Western Diagnostics, Murdoch, Western Australia, Australia.; 15 Institute of Lung Health and Immunity, Helmholtz Center, Munich, Germany.; 16 German Center for Lung Research, Munich, Germany.; 17 School of Pharmacy, University of Waterloo, Kitchener, Ontario, Canada.; 18 Department of Surgery and Cancer, Faculty of Medicine, Imperial College London, London, United Kingdom.; 19 Department of Physiological Sciences, College of Veterinary Medicine, Oklahoma State University, Stillwater, Oklahoma, USA.; 20 MD Anderson Cancer Center, Investigational Cancer Therapeutics Department, Houston, Texas, USA.; 21 Ludwig Maximilian University, Munich, Germany.; 22 Department of Medical Biology, University of Melbourne, Royal Parade, Melbourne, Victoria, Australia.

**Keywords:** Cell biology, Oncology, Breast cancer, Signal transduction, Ubiquitin-proteosome system

## Abstract

Activating mutations in *PIK3CA*, the gene encoding the catalytic p110α subunit of PI3K, are some of the most frequent genomic alterations in breast cancer. Alpelisib, a small-molecule inhibitor that targets p110α, is a recommended drug for patients with *PIK3CA*-mutant advanced breast cancer. However, clinical success for PI3K inhibitors (PI3Kis) has been limited by their narrow therapeutic window. The lipid phosphatase PTEN is a potent tumor suppressor and a major negative regulator of the PI3K pathway. Unsurprisingly, inactivating mutations in PTEN correlate with tumor progression and resistance to PI3K inhibition due to persistent PI3K signaling. Here, we demonstrate that PI3K inhibition leads rapidly to the inactivation of PTEN. Using a functional genetic screen, we show that this effect is mediated by a USP10-GSK3β signaling axis, in which USP10 stabilizes GSK3β, resulting in GSK3β-mediated phosphorylation of the C-terminal tail of PTEN. This phosphorylation inhibits PTEN dimerization and thus prevents its activation. Downregulation of GSK3β or USP10 resensitizes PI3Ki-resistant breast cancer models and patient-derived organoids to PI3K inhibition and induces tumor regression. Our study establishes that enhancing PTEN activity is a new strategy to treat *PIK3CA* mutant tumors and provides a strong rationale for pursuing USP10 inhibitors in the clinic.

## Abstract 2

PTEN mutations limit therapy efficacy, but enzymatic inactivation of PTEN by chemotherapy can also occur causing resistance. We propose a method to reactivate PTEN for improved outcomes.

## Graphical abstract

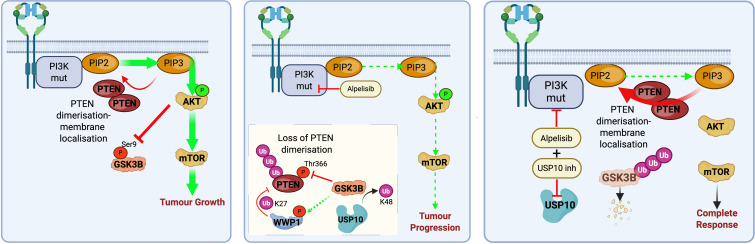

## Introduction

The phosphoinositide 3-kinase (PI3K) pathway is the most frequently altered pathway in human cancer. Numerous small molecules targeting the pathway have been evaluated in clinical trials (1–4), but PI3K pathway (re)-activation mediated by the presence of rare, preexisting, resistance-conferring genetic mutations or through adaptive or epigenetic mechanisms has limited overall therapeutic sensitivity and prevented clinical approval for most of these agents (5).

PI3Ks are a family of lipid kinases that regulate numerous cellular processes, including the cell cycle, survival, metabolism, motility, and genomic instability (6, 7). PI3Ks are holoenzymes consisting of a regulatory subunit (p85) and 3 possible class IA catalytic subunits p110α, β, and δ. All are activated downstream of RTKs or GPCRs (7). Activated PI3K phosphorylates phosphatidylinositol 4,5-bisphosphate [PI(4,5)P_2_] (PIP2) to generate the second messenger phosphatidylinositol 3,4,5-triphosphate (PIP3). PIP3 drives the activation of several downstream effector proteins with pleckstrin homology domains such as phosphoinositide-dependent kinase-1 (PDK1) and AKT/PKB. AKT is phosphorylated by PDK1 at T308 and subsequently by mTORC2 at S473, resulting in full activation of the protein (6). Once activated, AKT facilitates downstream signaling by phosphorylating a multitude of substrates driving several pathways, including the mTOR pathway marked by upregulation of the ribosomal protein S6. Some of these phosphorylation events are causally implicated in numerous malignancies. This includes the phosphorylation of glycogen synthase kinase-3 (GSK3) at Ser9, which suppresses GSK3 kinase activity (8, 9). Signal termination within the PI3K pathway is partly mediated by the phosphatase and tensin homolog (PTEN) (10). The principle catalytic function of PTEN is to dephosphorylate PIP3 to PIP2, leading to repression of PI3K-AKT downstream signaling (11). It is, therefore, unsurprising that genomic alterations in PTEN, and the maintenance of PIP3 activation, have been associated with PI3K inhibitor (PI3Ki) resistance in patients (12).

Recently, it was discovered that PTEN homodimerization at the plasma membrane represents a crucial step in enzyme activation (13, 14). PTEN dimerization is negatively regulated by several inhibitory posttranslational modifications, including the phosphorylation of S370, T382, T383, and T385 in the C-terminal tail of PTEN (15, 16). Phosphorylation promotes the interaction of the acidic tail of PTEN with its membrane-binding C2 domain, masking this domain to the lipid bilayer (16, 17). This closed conformation results in a catalytically inactive state, leading to persistent PIP3 upregulation and AKT activation. The absence of phosphorylation of these sites induces an open PTEN conformation, facilitating dimerization, and the binding of PTEN to the membrane (16). Hence, therapeutic approaches that promote PTEN dimerization may be promising strategies to reactivate its tumor-suppressor function.

Another mechanism regulating PTEN is ubiquitination (18). The majority of ubiquitination events, such as those mediated by K48 or K63 chain topologies, lead to proteasomal or lysosomal degradation of the substrate, respectively. Noncanonical ubiquitination, such as K27-linked ubiquitination, can interfere with protein-protein interactions (19, 20). Recently, it was demonstrated that K27 ubiquitination of PTEN by the WWP1 E3 ubiquitin ligase inhibited PTEN homodimer formation (15). Both genetic and pharmaceutical inhibition of WWP1 led to PTEN reactivation and downregulation of AKT signaling. Along with WWP1, several other E3 ligases influence PTEN stability and/or activation through monoubiquitination or polyubiquitination (18). The action of E3 ligases is directly opposed by deubiquitinating enzymes (DUBs). Several DUBs, including OTUD and USP family members, influence PTEN ubiquitination levels and thereby regulate either its stability or nuclear exclusion (21–23).

As with all targeted therapies, resistance is principally primed through reactivation of the targeted pathway either through mechanisms of primary or acquired resistance. However, the overall effectiveness of PI3Kis in the clinic has been surprisingly modest, partly due to a limited therapeutic index, indicating the potential presence of a rapid intrinsic feedback loop that restricts PI3Ki efficiency (24, 25). Therefore, in the present work, we set out to identify potential mechanisms that may regulate PTEN activation after PI3Ki treatment. In this study, we demonstrate that PI3K inhibition leads directly to the inactivation of the tumor-suppressor PTEN, limiting PI3K pathway downregulation. Moreover, we have characterized this mechanism and defined potential therapeutic strategies to circumvent it.

## Results

### PTEN expression does not correlate with PI3K downregulation following PI3K inhibition.

**Figure 1:**
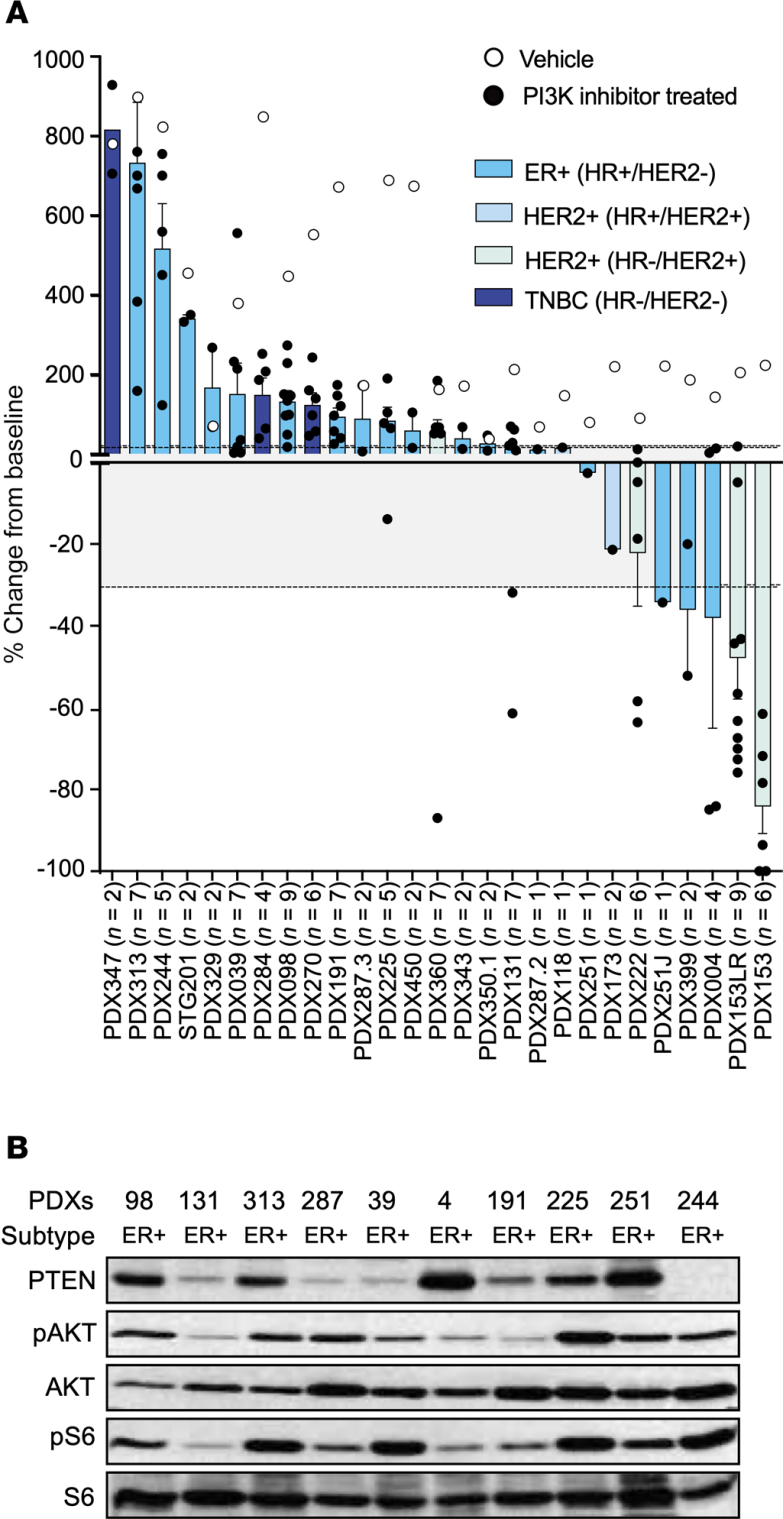
PI3K inhibitor monotherapy in PI3K mutant breast cancers. (**A**) Waterfall plot representing the growth of 27 PDXs treated with BYL719 (35 mg/kg) or GDC-0032 (25 mg/kg). The number of tumors (black circles) treated per model is indicated in the brackets (*n*). Bars represent the average of treated samples. Vehicle control average is represented as white circles. The percentage change from the initial volume is shown at day 35 of treatment. Dashed lines indicate the range of PD (>20%), SD (20% to –30%) and PR/CR (<–30%). Data are represented as mean values ± SEM. (**B**) Whole-cell lysates of ER^+^ patient derived xenografts derived from **A** were probed with indicated antibodies.

Patient-derived xenografts (PDXs) are clinically relevant preclinical models that can be used as surrogates to define mechanisms of resistance to targeted therapies. In a previous study, we interrogated PDXs obtained from ER^+^, HER2^+^, or triple-negative breast tumor specimens grown in immune-deficient mice with the PI3Ki BYL719 (alpelisib) and ribociclib (26). We have now extended our original data to include a total of 27 PDXs treated with BYL719, and 2 treated with the PI3Ki GDC-0032 (PDX329 and PDX350.1) (Figure 1A). The overall response to therapy was classified according to relative change in tumor volume upon treatment (similar to the Response Evaluation Criteria in Solid Tumors criteria) (27). In line with clinical observations with BYL719 or GDC-0032, we observed no complete responses, and we recorded partial responses in 5 PDX models (20%), stable disease in 6 PDX models (24%), and progressive disease in 16 models (56%) for a preclinical response rate (i.e., complete + partial responses) of 18% and a preclinical benefit rate (i.e., complete + partial responses + stable disease) of 40% (Figure 1A). As anticipated, tumor regression tended to be associated with low levels of phosphorylated AKT (pAKT) and downstream phosphorylated S6 (pS6) (Figure 1B) (28). In contrast, PDXs with sustained PI3K signaling tended to progress on treatment.

Since genomic loss of PTEN has been associated with PI3Ki resistance, we sought to correlate PTEN expression with response rate to PI3Kis (12, 29). As expected, we observed that PDX244, which harbors a PTEN deletion, had elevated levels of PI3K pathway activation and resistance to PI3K inhibition. In contrast, PDX004, which expresses high levels of PTEN, showed downregulation of the pathway and a therapeutic response. Notably, we also found 3 tumors (PDX098, PDX313, and PDX225) with relatively high PTEN levels and elevated levels of pAKT and pS6. All 3 tumors displayed progressive disease (Figure 1A and Supplemental Figure 1A). Sequencing of the original patient tumors identified a mutation in AKT^E17K^ in PDX313 but no genomic alterations in PTEN (Supplemental Figure 1B) (26). This suggests that in these tumors, PTEN may be ineffective in suppressing the PI3K pathway.

### USP10 is a positive regulator of PI3K signaling.

**Figure 2:**
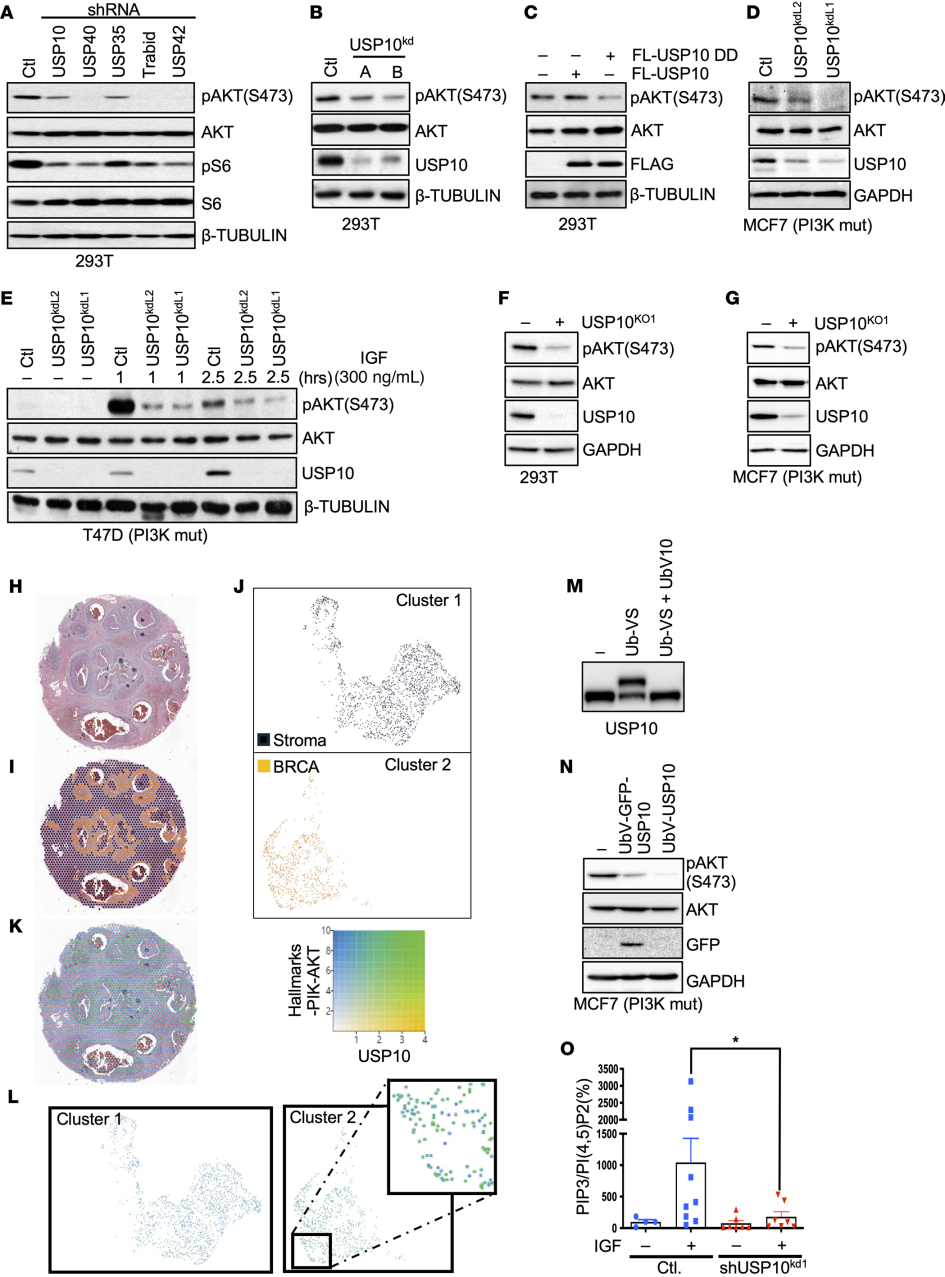
USP10 is a regulator of PI3K signaling in breast cancer. (**A**) Immunoblot analysis of HEK293T cells expressing shRNA vectors targeting the indicated DUBs. Ctl, control. (**B**) Immunoblot analysis of HEK293T cells expressing USP10 shRNA vectors A and B and probed with the indicated antibodies. (**C**) Immunoblot analysis in HEK293T cells transfected as indicated and probed with the indicated antibodies. (**D**) Immunoblot analysis of MCF7 cells expressing USP10 shRNA vectors L1 and L2 probed with the indicated antibodies. Mut, mutation. (**E**) T47D cells expressing USP10 shRNA vectors L1 and L2, treated with IGF (300 ng/mL) as indicated. Lysates were probed with the indicated antibodies. (**F** and **G**) Immunoblot analysis of HEK293T (**F**) or MCF7 cells (**G**) or USP10 KO counterparts (USP10^KO1^). Lysates were probed with the indicated antibodies. (**H**) Overview H&E image of human breast cancer FFPE sample used for spatial transcriptomic analysis. (https://www.10xgenomics.com/datasets/human-breast-cancer-ductal-carcinoma-in-situ-invasive-carcinoma-ffpe-1-standard-1-3-0). Data were visualized using the 10x Genomics Loupe browser (version 8.0). (**I**) K means = 2 clustering of human breast cancer FFPE sample used to segment tumor (BRCA indicated by orange) and stromal tissue (stroma indicated by black). (**J**) Uniform Manifold Approximation and Projection (UMAP) analysis of cell clustering of the 2 identified clusters displaying genetically defined subclones/differential gene expression of BRCA. (**K**) Correlation analysis of USP10 and PIK-AKT-Hallmark gene set (HALLMARK_PI3K_AKT_MTOR-SIGNLING, M5923) in UMAP on the breast cancer tissue section. USP10 and PIK-AKT signature-only expression areas are represented in yellow and blue, respectively; co-expression is visualized in green. (**L**) Co-expression of USP10 and the PIK-AKT signature from UMAP (**J**). Inset demonstrates co-expression of USP10 and PIK-AKT-Hallmark genes in BRCA. (**M**) Lysates of HEK293T cells expressing recombinant UbV10 incubated with HA-tagged Ub-VS for 30 minutes. Lysates were probed with a USP10 antibody. (**N**) MCF7 cells expressing ubiquitin binding variant–USP10 or luciferase control, as indicated. Lysates were probed with indicated antibodies. (**O**) Phospholipids were isolated from cells treated with DMSO or IGF (300 ng/mL) for 1 hour, and relative PIP3 and PI(4,5)P_2_ levels were quantified by ELISA. *P < 0.05 by 2-tailed *t* test.

Since PTEN activity is regulated by ubiquitination, we were interested first in determining the role of DUBs in the regulation of the PI3K pathway (16). Therefore, we performed an RNAi loss-of function screen using a library of shRNA vectors that consisted of pools of 4 nonoverlapping shRNAs designed to target approximately 94 known or putative DUBs (30) (Supplemental Figure 2, A and B, and Supplemental Table 1). HEK293T cells were transfected with each of the shRNA vector pools in duplicate, and immunoblot analysis was performed to analyze protein levels for pAKT/AKT and pS6/S6. The abundance of pAKT relative to total AKT or pS6 relative to total S6 were quantified. DUB shRNA pools that substantially decreased relative pAKT and pS6 levels were selected for subsequent rounds of selection. After 3 rounds of selection and validation, we observed a consistent decrease in pAKT in cells transfected with shRNA pools targeting USP10, USP42, USP35, Trabid, and USP40 (Figure 2A and Supplemental Figure 2C). Importantly, pS6 is downregulated in these cells as well, suggesting that the downregulation of pAKT is independent of any S6-mediated feedback loops. We chose to focus on USP10 because it was previously reported that USP10 can form a complex with PTEN (31, 32).

We first sought to establish the activity of the USP10-targeting shRNAs present in the shRNA pool. Therefore, we transfected these hairpins individually into HEK293T cells. Hairpins A, B, and D efficiently suppressed endogenous USP10 transcript and protein levels (Supplemental Figure 3, A and B). Similar results were observed in cells ectopically expressing FLAG-tagged USP10 (Supplemental Figure 3C), although hairpin D was not active in this experimental setting, because it targets the 3′-UTR of USP10, which is absent in the cloned transcript. Inhibition of USP10 with the validated knockdown (KD) vectors A and B efficiently downregulated pAKT, underscoring the validity of our screen (Figure 2B). To generate stable KD clones of USP10 we similarly tested an array of pLKO1-USP10 KD vectors (Supplemental Figure 3D). pLKO1-USP10 KD vectors L1, L2, and L3 were the most efficient at downregulating ectopically expressed USP10 and were used for the remainder of the experiments. Stable KD of USP10 using nonoverlapping hairpins decreased pAKT levels compared with a pLKO1-shGFP control (Supplemental Figure 3E). In contrast, overexpression of USP10 increased the concentration of pAKT, while the catalytically inactive USP10 mutant C424A (hereafter, USP10DD, for USP10 Dub Dead) decreased pAKT similar to USP10 KD (Figure 2C). This finding suggests the deubiquitinating activity of USP10 is required to regulate AKT phosphorylation.

Since the PI3K pathway is frequently hyperactivated in breast cancer typified by a high frequency of *PIK3CA* mutations, we analyzed the effect of USP10 depletion in the breast cancer cell lines MCF7 (PIK3CA-E545K) and T47D (PIK3CA-H1047R), which both harbor activating mutations in *PIK3CA*. In both cell lines, KD of USP10 substantially reduced pAKT levels (Figure 2, D and E). This also holds true upon the induction of PI3K-AKT pathway signaling with IGF, as seen in T47D cells (Figure 2E).

Previous reports indicated that USP10 has both positive and negative effects on PI3K, PTEN, and mTOR signaling (31, 33). Therefore, we sought to confirm our observed inhibitory effect of USP10 KD on PI3K signaling in breast cancer cells through 4 independent methods. First, we generated CRISPR-associated protein 9 (Cas9) KOs targeting *USP10* on chromosome 16 in HEK293T and MCF7 cells. USP10 KO decreased pAKT levels in both cell lines (Figure 2, F and G). Furthermore, RNA-Seq analyses of MCF7 CRISPR-Cas9 USP10 KO cells revealed a decrease in the levels of 3 established PI3K signatures (Supplemental Figure 4, A–D). Next, we probed publicly available spatial data sets comparing USP10 expression with PI3K gene expression signatures (Figure 2, H–L and Supplemental Figure 5, A–E) (34). In line with our RNA-Seq analysis, USP10 expression correlated with upregulation of PI3K gene expression signatures in tumor tissue only, with little to no USP10 expression being observed in the associated tumor stroma.

Lastly, we took advantage of a previously characterized ubiquitin binding variant 10 (UbV10) that specifically binds USP10 with high affinity and inhibits its deubiquitinating activity (35–37). To first confirm the inhibitory function of UbV10, we incubated cell extracts from HEK293T cells with or without recombinantly expressed UbV10, then the extracts were incubated with the ubiquitin active-site suicide probe ubiquitin vinyl sulfone (Ub-VS), which covalently modifies the catalytic site cysteine of DUBs (Figure 2M). As expected, UbV10 blocks the interaction of Ub-VS with the catalytic cysteine of USP10 and acts as an inhibitor of USP10 DUB activity. To further explore the inhibitory capability of UbV10 against USP10, we generated a 2′-*O*-methylation–capped mRNA expressing UbV10 or GFP-tagged UbV10 and ectopically expressed this in MCF7 cells. UbV10 expression downregulated AKT phosphorylation (Figure 2N).

Since AKT activation can also occur through mechanisms independent of PI3K activation, we sought to determine if the downregulation of AKT phosphorylation upon USP10 KD was due to inhibition of PIP3 (38). Changes in PIP3 levels were quantified using ELISA with PIP2 as a baseline control, because PIP2 is the most abundant phosphoinositide in the membrane and its levels are not influenced by PI3K activity (39). PIP3/PIP2 ratios were measured in T47D cells stably expressing a KD vector targeting USP10 and treated with IGF. IGF stimulation leads to an increase in PIP3 levels in control cells, but not in USP10 KD cells (Figure 2O). Taken together, our results indicate USP10 is a critical regulator of PI3K signaling in breast cancer.

### USP10 is linked to PI3Ki resistance in breast cancers with high PTEN expression.

Next, we sought to understand the role of USP10 for cell survival and transformation. An analysis of large-scale, genome-wide CRISPR-Cas9 KO data sets indicated that USP10 is classified as an essential gene for the survival of the majority of cancer cells as determined by both CRISPR-engineered regulatory element scoring (CERES) and CHRONOS analysis (Supplemental Figure 6, A–C) (40, 41). Indeed, depletion of USP10 in T47D cells substantially hampered the ability of these cells to grow in anchorage-independent growth conditions (Supplemental Figure 7, A and B).

**Figure 3:**
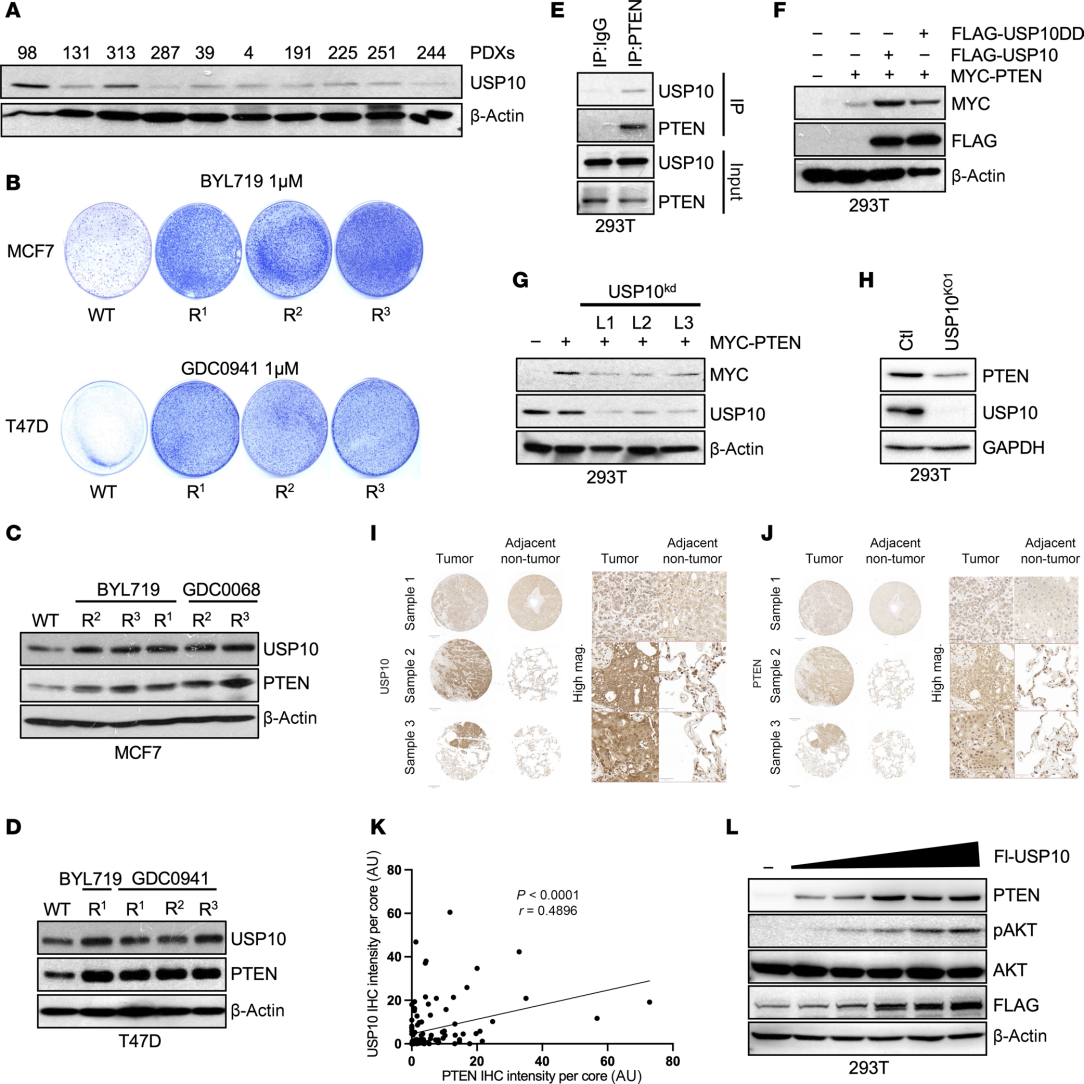
USP10 binds PTEN and regulates its protein expression. (**A**) Whole-cell lysates of PDXs from Figure 1B were probed with indicated antibodies. (**B**) Colony formation assay of MCF7 or T47D or corresponding PI3Ki-resistant clones R^1^, R^2^, and R^3^ treated with BYL719 (1 μM) or GDC0941 (1 μM) for 21 days. (**C** and **D**) Immunoblot analysis of MCF7 cells (**C**) or T47D cells (**D**) and corresponding resistant clones. Lysates were probed with the indicated antibodies. (**E**) HEK293T cells were lysed and immunoprecipitated with a PTEN antibody or IgG control (Ctl). Immunoprecipitated lysates and whole-cell extracts were probed with the indicated antibodies. (**F**) HEK293T cells were transfected with MYC-tagged PTEN and FLAG-tagged USP10 or FLAG-tagged USP10DD. After 48 hours, cells were lysed and blotted precipitates were probed with the indicated antibodies. (**G**) HEK293T cells were transfected with MYC-tagged PTEN and either USP10 shRNA vectors L1, L2, or L3. After 72 hours, cells were lysed and blotted precipitates were probed with the indicated antibodies. (**H**) Immunoblot analysis of HEK293T cells or HEK293T USP10 CRISPR KO cells (USP10^KO1^). Whole-cell lysates were probed with the indicated antibodies. (**I** and **J**) Representative immunohistology staining of tissue microarrays (TMAs) against endogenous USP10 (**I**) or PTEN (**J**) in sample and patient-matched nontransformed and human BRCA samples. Scale bar core overview: 1 mm; zoomed in: 50 μm. (**K**) Quantitative correlative analysis of protein abundance via IHC intensity in human BRCA using USP10 and PTEN in TMA sections. The analysis was conducted with the image analysis software QuPath (0.5.0) and manually by a trained pathologist. Pearson correlation between positive immunohistology signals of USP10 versus PTEN in human BRCA cores. *P* < 0.05 by 2-tailed *t* test. (**L**) HEK293T cells were transfected with increasing concentrations of FLAG-tagged USP10. After 48 hours, cells were lysed and blotted precipitates were probed with the indicated antibodies.

To test if USP10 is correlated with PI3Ki resistance in PDX tumors that exhibit high AKT activation, we explored the expression of USP10 in these tumor samples. Interestingly, 2 of the 3 tumors (PDX98 and PDX313) that have enhanced PTEN expression had high levels of USP10 (Figure 1B and Figure 3A). We next explored if USP10 upregulation is a common occurrence in PI3Ki-resistance models that express high levels of PTEN. We first generated MCF7 and T47D cells resistant to the PI3Kis BYL719 or GDC0941. Resistant cells were generated either by sequentially increasing the concentration of the PI3K pathway inhibitor over time to a maximum concentration of 1 μM (R^1^) or treating 3 days on and 3 days off with 500 nM inhibitor (R^2^) until resistance developed and then the drug concentration was increased to 1 μM using the same 3 days on–3 days off protocol (R^3^) (Figure 3B and Supplemental Figure 8, A–D). Interestingly, a subset of PI3Ki-resistant cell lines had a marked increase in PTEN expression with a corresponding upregulation of USP10 (Figure 3, C and D). Taken together, these data suggest USP10 may support PI3Ki resistance in breast cancers that retain high levels of PTEN.

### USP10 stabilizes PTEN protein levels by inhibiting protein dimerization.

To determine the molecular mechanism by which USP10 might affect PTEN and PI3K signaling, we first confirmed that endogenous USP10 co-immunoprecipitates with endogenous PTEN in HEK293T cells, (Figure 3E). Similarly, ectopically expressed USP10 and USP10DD interacted with PTEN (Supplemental Figure 9A). Under normal conditions, PTEN suppresses AKT activation. The observed upregulation of pAKT in tumors with high PTEN levels implies that USP10 may function through means other than directly influencing PTEN stability through deubiquitination. Nevertheless, we tested if USP10 affects PTEN ubiquitination. Depletion of USP10 did not enhance K48- or K63-associated PTEN ubiquitination, 2 ubiquitin chain linkages commonly associated with proteasomal- and lysosomal-mediated degradation, respectively (Supplemental Figure 9B). These data suggest USP10 is unlikely to directly influence PTEN stability through deubiquitination.

We noted, however, that USP10, but not USP10DD, expression led to an increase in the overall levels of PTEN (Figure 3F). Similar results were observed for endogenous PTEN (Supplemental Figure 9C). Concordantly, loss of USP10 expression downregulated PTEN protein levels in both HEK293T and MCF7 cells (Figure 3, G and H, and Supplemental Figure 9, D and E). MCF7 and T47D expressing UbV10 downregulated PTEN levels, as well (Supplemental Figure 9, F and G).

To determine the relevance of PTEN stability by USP10 in patients, we performed immunohistochemical staining of PTEN and USP10 on sequential sections of 107 primary or metastatic breast cancer samples (Figure 3, I and J). A significant positive correlation (*r* = 0.4896; *P* ≤ 0.0001) was observed between PTEN expression and USP10 expression in these samples (Figure 3K). We next sought to correlate USP10 expression with PTEN levels and upregulation of pAKT. As seen in Figure 3L, increasing expression of USP10 correlated with an upregulation of PTEN levels and enhanced AKT phosphorylation. Co-expression of USP10 with PTEN circumvented the negative regulation of PTEN on the PI3K-AKT pathway, an effect that was annulled in cells co-expressing USP10DD (Supplemental Figure 9, H and I). Furthermore, depletion of USP10 had no effect on pAKT levels in PTEN CRISPR KO cells (Supplemental Figure 9J).

**Figure 4:**
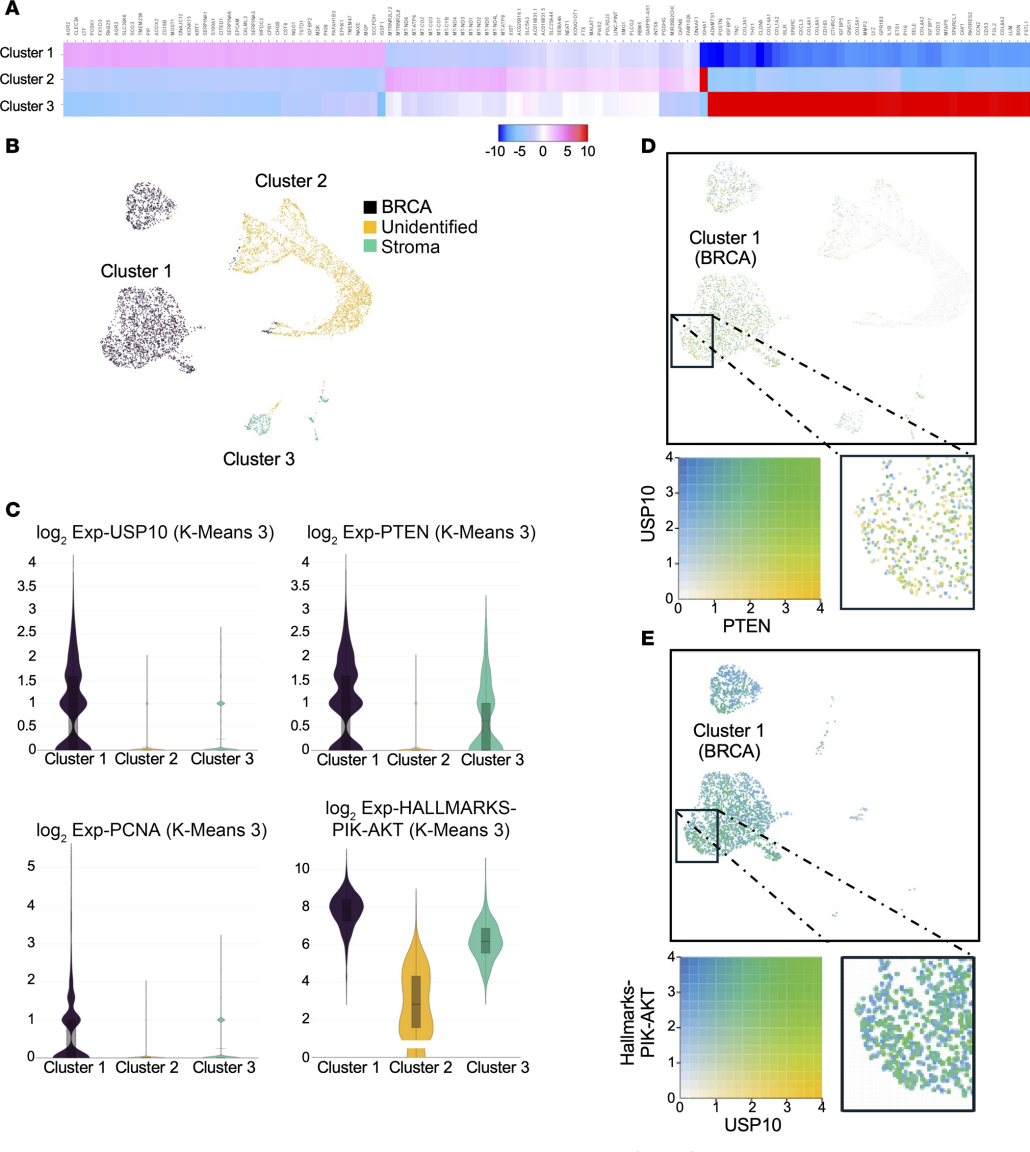
USP10 expression correlates with upregulation of PTEN and PI3K pathway signaling. (**A** and **B**) K means = 3 clustering of human invasive ductal carcinoma single-cell sequencing data set (https://www.10xgenomics.com/datasets/7-5-k-sorted-cells-from-human-invasive-ductal-carcinoma-3-v-3-1-3-1-standard-6-0-0). (**A**) Heatmap of differentially expressed genes in the K-means = 3 clusters. (**B**) USP10 expression in the different clusters is shown (black). Cluster 1 = cancer, cluster 2 = unidentified/mixed tissue, cluster 3 = stroma. (**C**) Violin plots of USP10, PTEN, PCNA, or PIK-AKT Hallmark genes in the K means = 3 clusters. Data demonstrate the predominant expression of USP10 and PCNA in cancer tissue (cluster 1), PTEN expression in cluster 1 (BRCA) and stroma (Cluster 3), and the expression of PIK-AKT hallmark genes in clusters 1 and 3, respectively. Data were visualized using the 10x Genomics Loupe browser (version 8.0). (**D**) Correlation analysis of the expression of USP10 and PTEN in the single-cell sequencing data set on human invasive ductal carcinoma. Cells with predominantly high expression of USP10 are represented in blue and PTEN-expressing cells are marked in yellow. Co-expression is visualized in green. The zoomed-in view shows the predominant co-expression of USP10 and PTEN in BRCA. Data were visualized using the 10x Genomics Loupe browser (version 8.0). (**E)** Correlation analysis of USP10 and genes of the PIK-AKT-Hallmark gene set on human invasive ductal carcinoma. Areas of high USP10 expression are represented in yellow, PIK-AKT-Hallmark gene sets only are marked in blue, co-expression is visualized in green. The zoomed-in view shows the predominant co-expression of USP10 and PIK-AKT-Hallmark genes in BRCA. Data were visualized using the 10x Genomics Loupe browser (version 8.0).

We next analyzed publicly available single-cell sequencing data comparing PTEN expression with USP10 expression and upregulation of PI3K gene expression signatures in breast cancer. Notably, USP10 expression was significantly enriched in cluster 1, which is defined by a distinct transcriptional signature associated with breast cancer, as evidenced by the expression of key breast cancer–related biomarkers (Figure 4, A–C, and Supplemental Figure 10A). Furthermore, PTEN expression was significantly upregulated in both clusters 1 and 3 (stroma associated) (Figure 4, A–C). Cluster 1 most strongly associated with PI3K pathway upregulation and USP10 co-expressed genes compared with clusters 2 and 3 (Figure 4C, and Supplemental Figure 10B). Importantly, single-cell sequencing analysis strongly suggests that a proportion of cells overexpressing PTEN and USP10 is associated with a transcriptional profile indicative of PI3K pathway activation (Figure 4, C–E). Collectively, these data suggest USP10 expression correlates with PTEN upregulation in a substantial fraction of human tumors harboring PI3K pathway activation.

**Figure 5:**
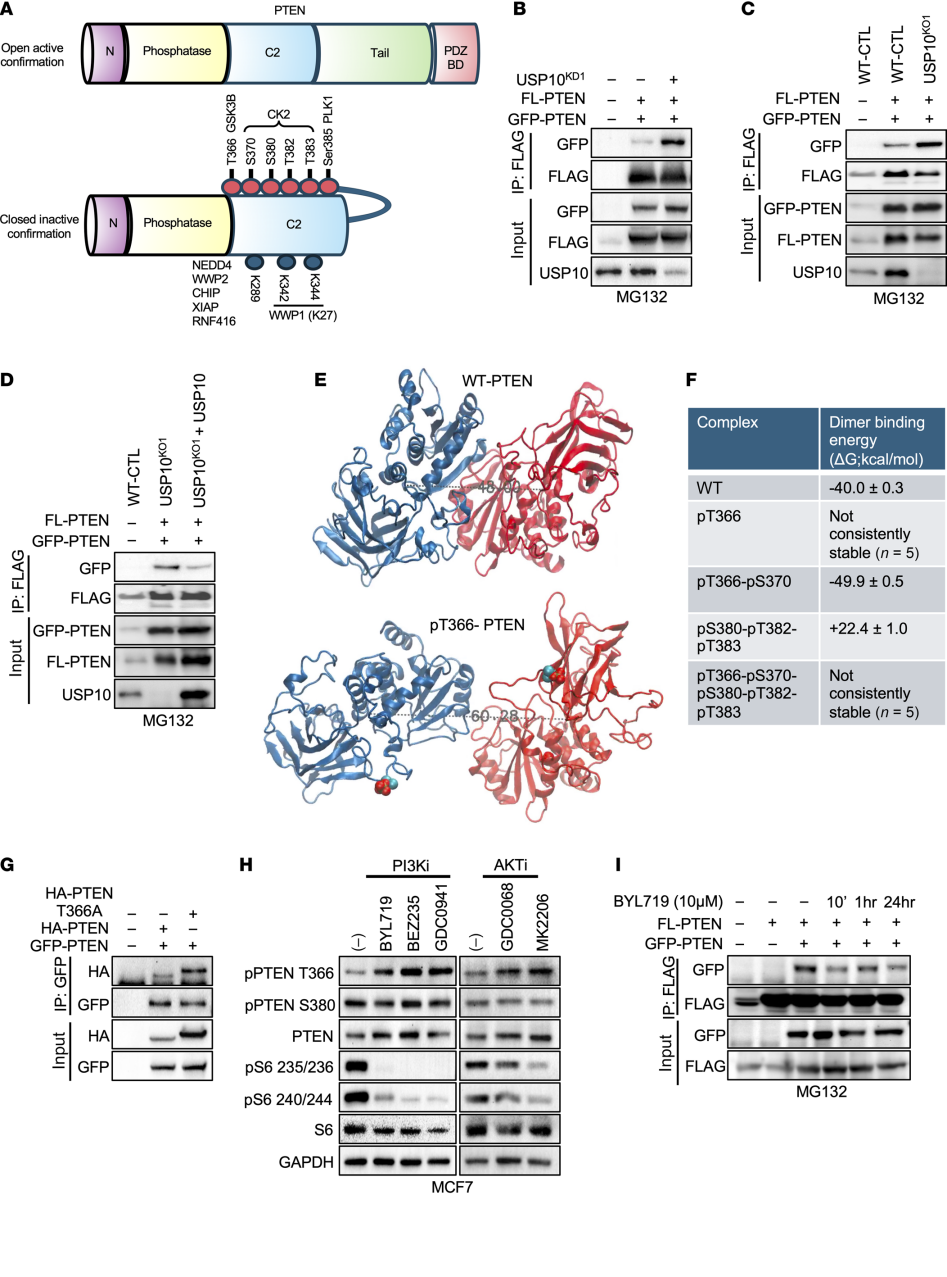
PI3Kis induce PTEN T366 phosphorylation, regulating its dimerization. (**A**) Schematic of PTEN in an open, active, unphosphorylated state (top) and PTEN phosphorylation in the C-terminal tail inducing a closed, inactive conformation (bottom). (**B**) Co-IP assay from total lysate of HEK293T cells, transfected with GFP-PTEN or FLAG-PTEN, with or without shRNA USP10^L1^. IP with anti-FLAG antibody. Western blot was probed with the indicated antibodies. (**C**) Co-IP assay from total lysate of HEK293T cells or HEK293T USP10^KO1^ cells transfected with GFP-PTEN and FLAG-PTEN. IP with anti-FLAG antibody. Western blot was probed with indicated antibodies. (**D**) Co-IP assay from total lysate of HEK293T, HEK293T USP10^KO1^, or HEK293T USP10^KO1^ cells ectopically expressing USP10, transfected with GFP-PTEN and FLAG -PTEN. IP with anti-FLAG antibody. Western blot was probed with the indicated antibodies. (**E**) Initially prepared model of unphosphorylated PTEN dimer or phosphorylated PTEN dimer (pT366) prior to molecular dynamics simulation. Phosphorylated residues are shown as spheres. Blue indicates monomer 1; red indicates monomer 2; teal/magenta indicates phosphothreonine residues. (**F**) Molecular mechanics — general born surface area binding energies for PTEN dimers with differential phosphorylation status. (**G**) Co-IP assay from total lysate of HEK293T cells, transfected with GFP-PTEN, HA-PTEN, or HA-PTEN T366A. IP with anti-GFP antibody. Western blot was probed with the indicated antibodies. (**H**) MCF7 cells treated for 24 hours with 1 μM indicated PI3Kis or AKT inhibitors (AKTi). Whole-cell lysates were collected and probed with the indicated antibodies. (**I**) MCF7 cells transfected with GFP-PTEN and FLAG-PTEN were treated with 10 μM BYL719, as indicated. IP with anti-FLAG antibody. Western blot was probed with indicated antibodies.

Multiple studies have shown that C-terminal tail phosphorylation of PTEN at Ser370, Ser380, Thr382, Thr383, and Ser385 by CK2 or PLK1 promotes a closed, inactive conformation that decreases PTEN dimerization and membrane localization (Figure 5A) (42–46). Furthermore, GSK3β has been demonstrated to phosphorylate PTEN at Thr366, promoting PTEN degradation, but, to our knowledge, has not previously been shown to regulate PTEN dimerization (45). Most importantly, the closed configuration of PTEN limits the accessibility for E3 ligases, which target the protein for degradation, and therefore enhances protein stability (16, 47). To test if USP10 regulates PTEN dimerization, we cotransfected 2 PTEN variants with different tags (GFP-PTEN [90 kDa] and FLAG-PTEN [55 kDa]) into HEK293T cells. shRNA-mediated depletion or CRISPR KO of USP10 in these cells promoted PTEN dimerization compared with control cells (Figure 5, B and C). In contrast overexpression of USP10 reduced PTEN dimerization (Figure 5D). This finding indicates USP10 likely upregulates AKT activation by promoting the closed, inactive conformation of PTEN, inhibiting its ability to dimerize.

To confirm the role of USP10 on PTEN activation and stabilization, we co-expressed USP10 with WT-PTEN or a PTEN mutant in which 4 C-terminal phosphorylation sites are mutated to alanines (PTEN-A4; S380A/T382A/T383A/S385A). PTEN-A4 expression was much lower compared with WT-PTEN, likely because of increased activation of the protein (Supplemental Figure 11A) (44). Although USP10 can promote the stability of WT-PTEN, as we observed, it does not enhance the stability of PTEN-A4. This finding suggests USP10 might affect PTEN dimerization by influencing its C-terminal tail phosphorylation (Supplemental Figure 11A).

### Phosphorylation of residue Thr366 regulates PTEN dimerization.

Since the C-terminal tail functions in PTEN homodimer stabilization, we sought to understand the effect of phosphorylation on homodimerization, using molecular dynamics simulation combined with binding energy analysis (Figure 5, E and F, and Supplemental Videos 1–5). As previously reported, C-terminal tail phosphorylation decreases PTEN dimer stability compared with WT (Figure 5F and Supplemental Videos 1 and 2) (13, 47, 48). Critically; however, phosphorylation at Thr366 alone completely destabilized dimer formation, an effect that, curiously, was annulled when both Thr366 and Ser370 were phosphorylated (Supplemental Videos 3 and 4). In line with these results, co-expression of GFP-PTEN with a PTEN mutant (T366A) promoted PTEN dimer formation compared with WT-PTEN (Figure 5G). Together, these data suggest phosphorylation of Thr366 is a critical regulator of PTEN dimerization.

### Treatment with a PI3Ki affects phosphorylation of PTEN residue Thr366.

Some of our PDX models that were resistant to PI3Kis displayed increased upregulation of PTEN. Therefore, we sought to understand if PTEN C-terminal tail phosphorylation was altered in breast cancer cell lines after exposure to the PI3Kis BYL719 (PI3K), BEZ235 (pan-PI3K/mTOR), or GDC-0941 (pan-PI3K). PI3K or AKT inhibition consistently upregulated the phosphorylation of Thr366 but not phosphorylation of Ser380, Thr382, and Thr383 (Figure 5H and Supplemental Figure 11B). Next, we analyzed PTEN phosphorylation upon exposure to PI3Ki over time. Interestingly, the PI3Ki and both pan-PI3Kis rapidly induced phosphorylation of Thr366, but only the pan-PI3Kis enhanced phosphorylation of Ser380, Thr382, and Thr383, suggesting multiple modes of regulation of PTEN C-terminal tail phosphorylation (Supplemental Figure 11, C–E). Phosphorylation of all C-terminal tail residues decreased over time and then was again upregulated at 24 hours. Similar results were observed in PDXO098 and PDXO479 tumors (Supplemental Figure 11F). Interestingly, Thr366 phosphorylation and USP10 expression were maintained at 24 hours in the BYL-resistant PDXO098 but not in the PI3Ki-sensitive PDXO479.

Next, we investigated if PI3K inhibition influenced PTEN dimerization. In line with upregulation of PTEN^Thr366^, PI3Ki treatment substantially decreased the ability of PTEN to form homodimers (Figure 5I). Taken together, these data suggest PTEN activity is inhibited after PI3K pathway downregulation.

### USP10 regulates GSK3β stability and phosphorylation of PTEN residue Thr366.

**Figure 6:**
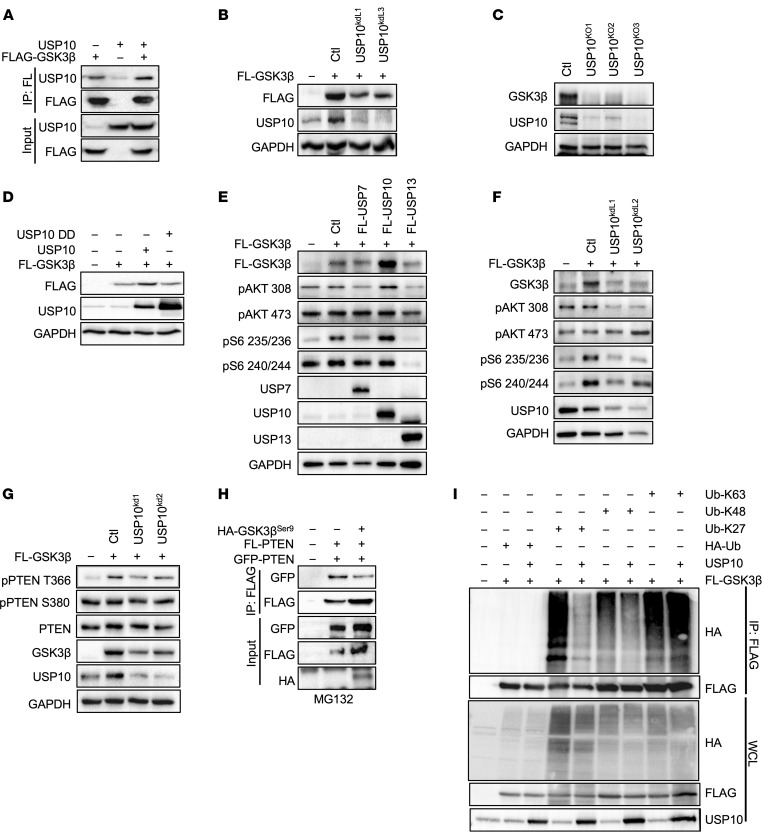
USP10 stabilizes GSK3β to regulate downstream PI3K signaling. (**A**) Co-IP assay from total lysate of HEK293T cells, transfected with FLAG-GSK3β and/or USP10. IP with anti-FLAG antibody. Western blot was probed with indicated antibodies. (**B**) Immunoblot analysis of HEK293T cells expressing FLAG-GSK3β with or without shRNA targeting USP10 and probed with the indicated antibodies. Ctl, control. (**C**) Immunoblot analysis of HEK293T cells or HEK293T USP10 CRISPR KO clones (USP10^KO1^, USP10^KO2^, USP10^KO3^). Whole-cell lysates were probed with the indicated antibodies. (**D**) Immunoblot analysis in HEK293T cells expressing FLAG-GSK3β, with or without FLAG-USP10 or FLAG-USP10DD. Whole-cell extracts were probed with the indicated antibodies. (**E**) Immunoblot analysis in HEK293T cells expressing FLAG-GSK3β and either FLAG-USP7, FLAG-USP10, or FLAG-USP13. Whole-cell extracts were probed with the indicated antibodies. (**F**) Immunoblot analysis in HEK293T cells expressing FLAG-GSK3β and either shRNA USP10^L1^ or USP10^L2^. Whole-cell extracts were probed with the indicated antibodies. (**G**) Immunoblot analysis in HEK293T cells expressing FLAG-GSK3β and either shRNA USP10^L1^ or USP10^L2^. Whole-cell extracts were probed with the indicated antibodies. (**H**) Co-IP assay from total lysate of HEK293T cells, transfected with GFP-PTEN, HA-PTEN, and HA-GSK3β^Ser9^. IP with anti- FLAG antibody. Western blot was probed with indicated antibodies. (**I**) HEK293T cells transfected with FLAG- GSK3β, HA-tagged ubiquitin, or its mutant isoforms K27, K48, or K63, in the presence or absence of CMV-USP10. Lysates were immunoprecipitated with anti-FLAG affinity resin, resolved by SDS-PAGE, and probed with indicated antibodies.

To further explore USP10-mediated regulation of PTEN, we asked if USP10 could form complexes with either the E3 ligase WWP1 or the 3 known kinases that target C-terminal tail phosphorylation [namely GSK3β(T366), ref. 45; CK2(S380/T382/T383), refs. 43 and 49; and PLK1(S385), ref. 50]. Interestingly, USP10 formed complexes with WWP1, PLK1, and GSK3β, but not CK2 (Figure 6A, and Supplemental Figure 12, A–C). Preliminary analyses indicated USP10 did not alter the protein stability of WWP1 or PLK1 (data not shown). However, KD of USP10 diminished the levels of ectopically expressed GSK3β (Figure 6B).

To validate the regulation of GSK3β by USP10, we analyzed GSK3β protein levels in USP10 KO cells. GSK3β protein levels were decreased in all 3 USP10 KO clones (Figure 6C). In contrast, ectopic expression of USP10, but not USP10DD, upregulated GSK3β levels (Figure 6D). These data show that USP10 regulates the stability of GSK3β.

A number of DUBs, including USP7 and USP13, have been demonstrated to regulate PTEN localization and stability (18, 19). Therefore, we analyzed if either of these DUBs also affected GSK3β protein levels, but only USP10 noticeably enhanced GSK3β stabilization (Figure 6E). Interestingly, the increase in GSK3β protein stability was associated with an increase in phosphorylation of AKT (Thr308) and S6 (Ser235/236) (Figure 6E). In contrast, downregulation of GSK3β mediated by USP10 KD led to downregulation of AKT and S6 phosphorylation (Figure 6F). As expected, GSK3β-mediated phosphorylation of the PTEN residue Thr366 was attenuated in cells with decreased expression of USP10 (Figure 6G). In accordance with the increase in Thr366 phosphorylation, ectopic expression of a hyperactive form of GSK3β (GSK3B^Ser9A^) decreased PTEN dimerization and upregulated overall PIP3 levels (Figure 6H and Supplemental Figure 12D).Taken together, these data suggest the effect of USP10 on PTEN stability, dimerization, and downstream AKT activation is facilitated by phosphorylation of PTEN mediated by GSK3β.

To study if USP10 deubiquitinates GSK3β, we cotransfected GSK3β, and USP10 or USP10DD, with an HA-tagged Ub construct. USP10 markedly decreased GSK3β ubiquitination, whereas USP10DD did not alter the ubiquitination status of GSK3β (Supplemental Figure 12E). Next, we used K27, K48, or K63 mutant ubiquitin constructs only capable of generating representative singular chain linkages (all other lysines are mutated to arginine) to test their individual effects on ubiquitination in cells that overexpress USP10. USP10 decreased the overall levels of K48- and K27-incorporated ubiquitin, but not K63, suggesting that USP10-mediated stabilization of GSK3β is likely due to decreased K48 ubiquitination (Figure 6I). Taken together, these results suggest USP10 deubiquitinates and stabilizes GSK3β, resulting in increased PTEN phosphorylation at PTEN residue Thr366 and PTEN inhibition.

### USP10 mediates PTEN K27-linked polyubiquitination.

PTEN C-terminal tail phosphorylation at Thr366 promotes ubiquitination (51). Recently, it has also been demonstrated that K27-linked ubiquitination by the E3 ligase WWP1 inhibits PTEN dimerization, membrane recruitment, and function (15). Therefore, we explored if the effect of USP10 on dimerization may be attributed to altered K27-linked ubiquitination. In line with our previous results demonstrating that USP10 decreases PTEN dimerization, only K27 ubiquitination chains were substantially upregulated in the presence of USP10 (Supplemental Figure 13A). Since USP10 enhances PTEN K27 ubiquitination rather than deubiquitinating PTEN, we speculated that USP10-GSK3β may also influence WWP1. IP of WWP1 and GSK3β demonstrated that GSK3β can form a complex with WWP1, resulting in WWP1 phosphorylation (Supplemental Figure 13, B and C). In addition, short-term treatment with BYL719 enhanced PTEN K27 ubiquitination (Supplemental Figure 13D).

Next, we sought to understand if the decreased dimerization mediated by GSK3β alters PTEN phosphatase activity. Indeed, PIP3 analysis indicated that co-expression of GSK3β^Ser9^ almost completely inhibited PTEN phosphatase activity but not in PTEN cells harboring a phosphate null mutation, T366A (Supplemental Figure 13E). These data indicate USP10-mediated regulation of GSK3β likely results in a dynamic interplay between PTEN K27 ubiquitination and C-terminal tail phosphorylation, as previously suggested (16). An effect induced after the addition of PI3Kis.

### USP10 is upregulated in breast cancer and contributes to PI3Ki resistance.

**Figure 7:**
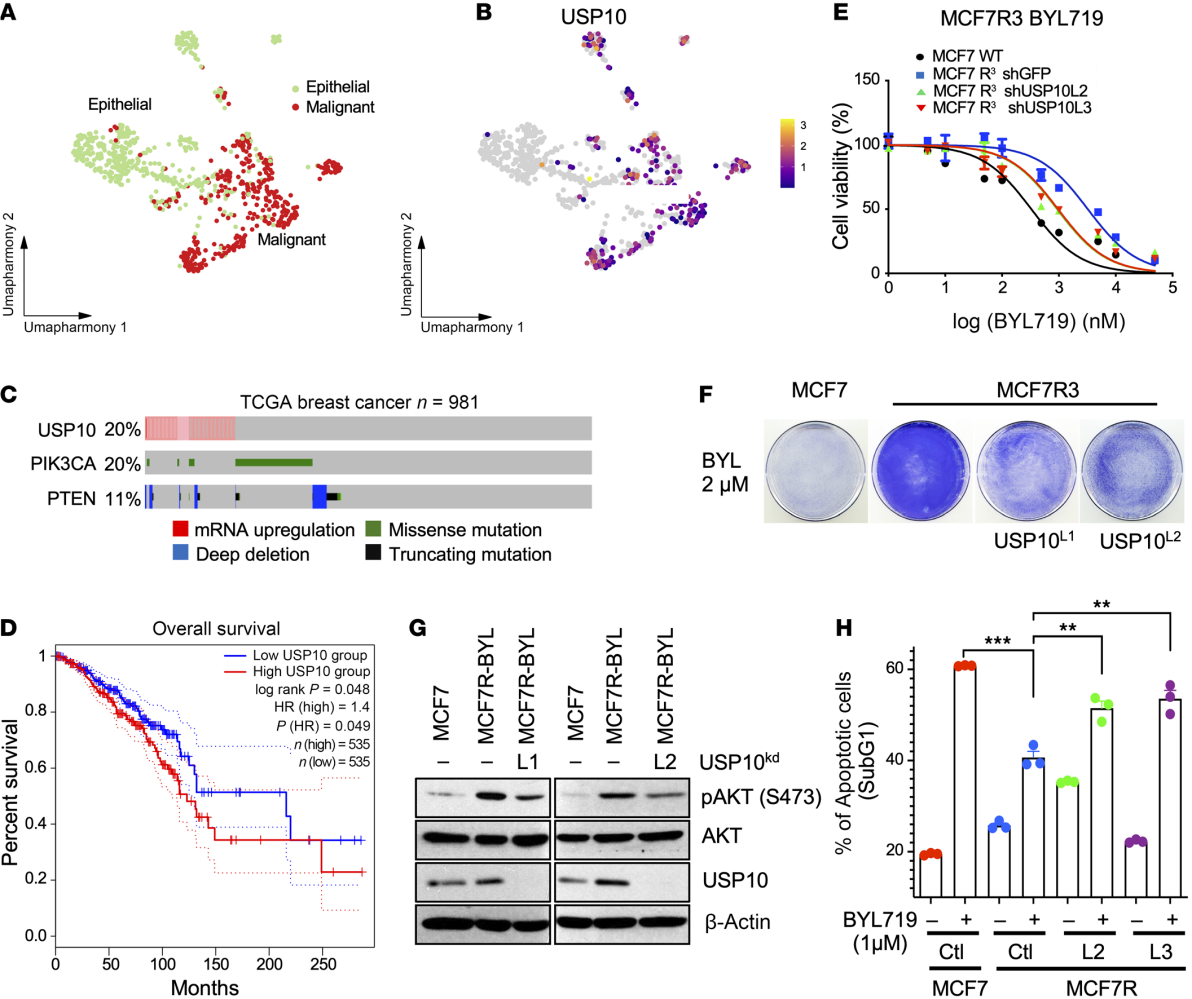
USP10 is upregulated in breast cancer and correlates with PI3Ki resistance. (A) A batch-corrected Uniform Manifold Approximation and Projection (UMAP), representing malignant and normal epithelial cells from patients with triple-negative breast cancer (53). The cells are colored to depict their respective cell-type annotations: epithelial (green) or malignant (red). (**B**) A UMAP illustrating the normalized expression of USP10 across all cells extrapolated from **A**. (**C**) Matrix heatmap generated using cBioportal showing genetic alterations in PIK3CA, PTEN, and upregulation of USP10 mRNA. (**D**) Kaplan-Meier curves from TCGA data extrapolated from the GEPIA2 genomics analysis and visualization platform showing probability of overall survival of patients with breast cancer with a higher copy number of USP10 is significantly less than those with a lower level of USP10 (*P* = 0.049). HR, hazard ratio. (**E**) MCF7 PI3Ki-resistant cells expressing shRNA USP10L2 or USP10 L3 or shGFP were treated with escalating doses of BYL719, as indicated, for 72 hours. Viability was assayed using CellTiter-Glo as described by the manufacturer. Data represent the mean of 5 replicates. (**F**) Colony formation assay of MCF7-WT or MCF7-PI3Ki–resistant clone R^3^ stably expressing shRNA USP10^L1^ or USP10^L2^ or a shRNA GFP control. Cells were treated with BYL719 (2 μM) for 21 days. (**G**) Immunoblot analysis of MCF7 or MCF7R3 cells expressing either shRNA USP10^L1^ or USP10^L3^. Whole-cell extracts were probed with the indicated antibodies. (**H**) Quantification of the sub-G1 population after treatment with BYL719 (1 μM); the mean SEM of 3 independent experiments is reported. Dunnett’s multiple comparison test was used to compare treated populations. **P < 0.01, ***P < 0.001.

USP10 acts as both an oncogene and tumor suppressor (52). We investigated if the oncogenic role of USP10 we identified is a relevant factor in human breast cancer and PI3Ki resistance. Similar to our spatial analyses, single-cell RNA profiling analysis of 6 patients with triple-negative breast cancer extrapolated from the report by Karaayvaz et al. (53) indicated that cells annotated as malignant, based on marker genes and inferred copy number aberrations, had significantly higher expression of USP10 compared with epithelial cells (Figure 7, A and B, and Supplemental Figure 14, A–C). USP10 expression was also associated with tumor aggressiveness (Supplemental Figure 14D). Moreover, in line with our results for PDX98 and PDX313 and our single-cell and spatial analyses, within The Cancer Genome Atlas (TCGA) database (TCGA cBioportal) 20% of patients with breast cancer had upregulation of USP10 mRNA levels (Figure 7C). Stratification of patients into 2 groups based on the expression of USP10 determined that patients with high expression of USP10 had significantly reduced overall survival (Figure 7D). Taken together, these results show USP10 is upregulated in a fraction of breast cancers and is linked to a more malignant phenotype.

To investigate if USP10-mediated inactivation of PTEN might contribute to PI3Ki resistance, we first examined if USP10 depletion by shRNAs could resensitize MCF7 or T47D PI3Ki-resistant cell lines to PI3Kis. Thus, we generated BYL719 and GDC0941 dose-response curves in the presence or absence of USP10 KD. We observed that USP10 KD decreased the BYL719 half-maximal growth inhibitory concentration (GI_50_) (50% cell death) by 3-fold and the GDC0941 GI_50_ by 4-fold (Figure 7E and Supplemental Figure 15A). Similar results were observed in USP10-KD, PI3Ki-resistant T47D cells (Supplemental Figure 15, B–E). We confirmed these observations in colony formation assays in BYL719-resistant MCF7 cells (Figure 7F). Likewise, CRISPR-mediated USP10 KO decreased the overall proliferative potential of MCF7 cells when treated with various PI3Kis (Supplemental Figure 15F). Furthermore, analysis of the intercellular responses in MCF7 PI3K-resistant cells to USP10 depletion showed a marked decrease in pAKT levels upon USP10 depletion (Figure 7G). USP10-depleted cells also exhibited an increase in the accumulation of cells in sub-G1 compared with their resistant counterparts after BYL719 treatment (Figure 7H), indicative of an increase in cell death. These data suggest USP10 plays a critical role in PI3Ki resistance in breast cancer and that loss of USP10 resensitizes resistant cells to PI3Kis.

### Spautin-1 resensitizes PI3Ki-resistant cells.

A number of nonspecific inhibitors against USP10 have been developed, including the potent autophagy inhibitor-1 (Spautin-1), which inhibits both USP10 and USP13 with an IC_50_ of 0.6–0.7 μM (54). To confirm the effect of Spautin-1 on USP10, we tested if Spautin-1 could inhibit the ability of the Ub-VS to bind to the catalytic cleft of USP10. Curiously coincubation with Spautin-1 had no effect on Ub-VS binding (data not shown), suggesting Spautin-1 does not act as a catalytic inhibitor of USP10. Therefore, we tested if USP10 is required for the antiproliferative effects of Spautin-1. As previously shown, Spautin-1 decreased the proliferative capacity of MCF7 cells in nutritionally deprived conditions (54) but not in cells depleted for USP10 (Supplemental Figure 15G) (54). Our observation that Spautin-1 was ineffective in MCF7 cells depleted for USP10 suggested USP10 is required for the antiproliferative effects of Spautin-1 and that Spautin-1 may function either as an indirect or allosteric inhibitor of USP10.

To confirm if the combination of Spautin-1 and PI3K inhibition exhibits synergistic activity in PI3Ki-resistant breast cancer cell lines, we treated each of the parental and resistant cell lines with increasing concentrations of Spautin-1 and BYL719 alone or in combination. As previously demonstrated (28), PI3K inhibition alone was relatively ineffective at inhibiting the proliferation of PI3Ki-resistant cells (Supplemental Figure 16A). However, we observed a synergistic interaction between Spautin-1 and BYL719 in suppressing cell proliferation as determined by a zero interaction potency (ZIP) synergy score of 2.613 (Supplemental Figure 16, A–C). We further confirmed these observations in colony formation assays in BYL719-resistant MCF7 cells treated with BYL719 alone, Spautin-1 alone, or the combination (Supplemental Figure 16D).

Next, we sought to validate this interaction in PDX-derived organoids (PDXOs) grown in short-term, 3-dimensional ex vivo cultures on a laminin-rich extracellular matrix. The antiproliferative activity of BYL719 and Spautin-1 alone or in combination was measured by PDXO metabolic activity. Importantly, Spautin-1 decreased the viability alone and further decreased the viability of PDXO350.2 when in combination with BYL719 (Supplemental Figure 16E). Furthermore, cotreatment with BYL719 and Spautin-1 downregulated the AKT pathway compared with either inhibitor alone (Supplemental Figure 16F). Taken together, these results suggest USP10 inhibition may sensitize PI3Ki-resistant tumors to PI3K pathway downregulation and tumor regression.

### GSK3β inhibition resensitized PI3Ki-resistant cells to PI3K inhibition.

**Figure 8:**
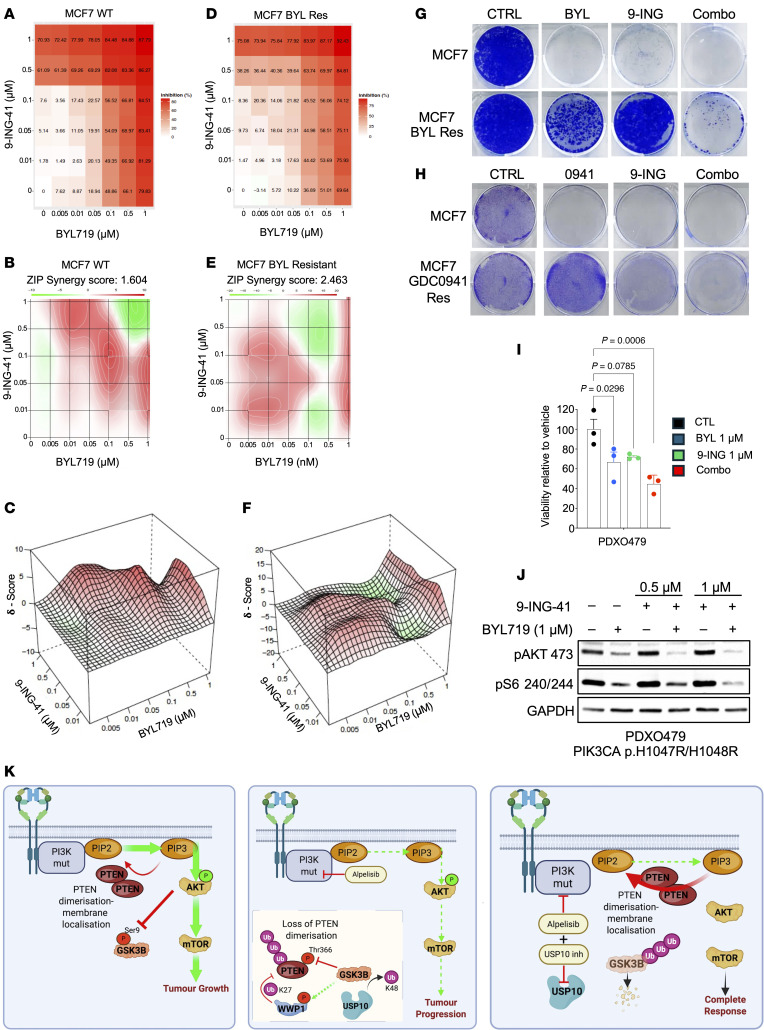
GSK3β inhibition resensitizes PI3Ki-resistant cells to PI3Kis. (**A**) A dose matrix of BYL719 with 9-ING-41 was created in parental MCF7 cells. Viability was assessed after 5 days. Percentage inhibition at each dose is presented. (**B** and **C**) Synergy analysis of BYL719 and 9-ING-41 in an MCF7 cell line showing the ZIP synergy score, as analyzed using SynergyFinder software. (**D**) A dose matrix of BYL719 with 9-ING-41 was created in MCF7 BYL719-resistant cells. Viability was assessed after 5 days. Percentage inhibition at each dose is presented. Res, resistant. (**E** and **F**) Synergy analysis of BYL719 and 9-ING-41 in an MCF7 BYL719-resistant cell line, showing the ZIP synergy score, as analyzed using SynergyFinder software. (**G**) Colony formation assay of MCF7 or MCF7 BYL719-resistant clones treated with DMSO, BYL719 (1 μM), 9-ING-41 (1 μM), or the combination (Combo) for 21 days. Ctrl, control. (**H**) Colony formation assay of MCF7 or MCF7 GDC0941-resistant clones treated with DMSO, GDC0941 (1 μM), 9-ING-41 (1 μM), or the combination for 21 days. (**I**) Viability assay of PDXO479 treated with BYL719 (1 μM), 9-ING-41 (1 μM), or the combination for 5 days. (**J**) Immunoblot analysis of PDXO479 treated in **I**. Whole-cell extracts were probed with the indicated antibodies. (**K**) Schematic overview of GSK3β-USP10 regulation of PTEN dimerization following PI3K inhibition (inh). Mut, mutation.

Since the inhibitory phosphorylation on GSK3β is lost after PI3K inhibition and GSK3β phosphorylates PTEN at Thr366, a site we demonstrate to hinder PTEN dimerization and consequently upregulates AKT signaling, we investigated whether chemical inhibition of GSK3β could amplify the impact of PI3Kis in suppressing the AKT pathway. As expected, treatment with the PI3Ki BYL719 potently decreased AKT phosphorylation and downstream S6 phosphorylation (Supplemental Figure 17A). However, the cotreatment of the GSK3β inhibitor 9-ING-41 noticeably downregulated the pathway, compared with PI3K monotherapy treatment (Supplemental Figure 17A). Importantly, the addition of 9-ING-41 also resensitized PI3Ki-resistant MCF7 cells to PI3K inhibition at clinically relevant concentrations, as determined by the downregulation of S6 phosphorylation (Supplemental Figure 17B). In concordance, we observed a synergistic interaction between 9-ING-41 and BYL719 in suppressing cell proliferation in WT and PI3Ki-resistant cell lines, as determined by ZIP synergy score (Figure 8, A–F). This interaction was further validated in PI3Ki-resistant cells and in 2 PI3Ki-sensitive PDXO models, in which cotreatment with a GSK3β inhibitor further diminished PI3K pathway activation, as determined by pAKT and downstream pS6 protein levels, leading to a greater reduction in proliferative capacity compared with BYL719 alone (Figure 8, G–J, and Supplemental Figure 18, A and B). Notably, the combination of the GSK3β inhibitor 9-ING-41 with the USP10 inhibitor Spautin-1 was antagonistic in MCF7 WT cells and their PI3Ki-resistant counterparts (Supplemental Figure 19, A–F). These results demonstrate that targeted inhibition of GSK3β amplifies the effectiveness of PI3Kis in suppressing the pathway and can potentially function in combination with PI3Kis to overcome PI3Ki-mediated feedback loops.

## Discussion

In this study, we have shown that the inhibition of PI3K/AKT signaling leads to aberrant phosphorylation of Thr366 on the C-terminal tail of PTEN, a residue we demonstrate to be critical for PTEN dimerization. This phosphorylation appears to form part of a negative feedback loop that regulates overall PIP3 levels in cells. Under such a scenario, AKT-mediated phosphorylation of GSK3β on the inhibitory Ser9 residue of GSK3β (8) limits the ability of GSK3β to phosphorylate its downstream substrates, including PTEN (Figure 8K). Upon selective PI3K/AKT inhibitor treatment, GSK3β phosphorylation on Ser9 is lost, activating the kinase. GSK3β phosphorylates T366 on PTEN, inhibiting PTEN. Here, we establish that these processes are potentially linked and demonstrate that PI3K inhibition directly leads to phosphorylation of Thr366 and inhibition of PTEN. This results in the maintenance of higher PIP3 levels and amplified downstream AKT signaling. These findings suggest a rationale for the limited therapeutic index of PI3K pathway inhibitors in the clinic and helps explain why, in dose-escalation studies, achieving effective target engagement with PI3K compounds consistently exceeds tolerable limits (4).

As part of our studies, we performed a deubiquitinating enzyme RNAi screen to identify DUBs that regulate AKT activity or downstream signaling, as represented by changes in S6 phosphorylation. The reasoning behind this was 2-fold. First, we wanted to understand the puzzling upregulation of PTEN in PDXOs resistant to PI3Kis. Second, a number of studies have demonstrated the role of E3 ligases affecting the PI3K pathway components negatively altering the activity of the PI3K pathway suggesting that targeted protein degraders, such as DUB inhibitors, may be used as a rational approach to target PI3K mutant cancers (55, 56). Notably, it has recently been demonstrated that PI3Kis enhance the degradation of the mutant p110α (H1047R) isoform, partially explaining the increased efficacy of these compounds over other PI3K pathway inhibitors in the clinic (25, 57).

Here, we identify the deubiquitinating enzyme USP10 as a inhibitor of PTEN dimerization. Specifically, USP10 deubiquitinates and stabilizes GSK3β, resulting in increased phosphorylation of Thr366 on PTEN, which interferes with protein dimerization and limits the catalytic activity of PTEN. To our knowledge, this is the only DUB that plays a role in GSK3β stability. In addition, we demonstrate that increased expression of USP10 upregulates K27-linked ubiquitination of PTEN, a mechanism associated with decreased PTEN dimerization. Recently, it was demonstrated that WWP1-mediated K27 ubiquitination of PTEN suppresses dimerization, plasma membrane recruitment, and tumor suppressive function (15). USP10 and GSK3β can form complexes with WWP1, with increased activation of GSK3β leading to WWP1 phosphorylation. Although we do not know the direct effect of GSK3β-mediated phosphorylation on WWP1 catalytic activity, this work prompts various inquiries regarding the enzymatic kinetics of PTEN concerning C-terminal tail phosphorylation, K27-linked ubiquitination, and PTEN dimerization. Moreover, whereas phosphorylation of Thr366 alone led to a reduction in dimerization, secondary phosphorylation at Ser370 completely annulled the effect of Thr366 phosphorylation on PTEN dimerization. Introducing yet another layer of complexity to the system. Here, we demonstrate that USP10 binds WWP1, GSK3β, PLK1, and PTEN, suggesting that USP10 might operate through multiple interdependent pathways to impede PTEN dimerization.

Deleterious mutations in the tumor-suppressor PTEN mimic activating mutations in the oncogene PIK3CA, both driving elevated intracellular levels of PIP3 and promoting oncogenic signaling. Curiously, recent sequencing studies and expression analyses, including the work presented here, have revealed distinct subsets of breast cancer with unexpectedly high PTEN expression levels and that correlate with upregulation of the PI3K signaling (58), thus suggesting a complex, yet unresolved, relationship between PTEN abundance and PI3K signaling activation. PTEN can adopt a closed, inactive conformation that impedes dimerization, further amplifying PIP3 levels and impacting downstream signaling. Notably, this closed conformation of PTEN reduces accessibility to E3 ligases, which target the protein for degradation, thereby enhancing its stability (16, 47). Since USP10 expression is upregulated in human breast cancer and correlates with PI3K activity in a large fraction of cells expressing heightened levels of PTEN, the elevated USP10 expression may help explain the meaning of this genetic selection in cancer and the relationship between PTEN upregulation and PI3K activity.

Genetic ablation of USP10 increased PTEN dimerization; therefore, it would be important to determine if USP10 loss would also enhance the formation of PTEN mutant heterodimers in which catalytically inactive mutations of PTEN inhibit WT PTEN, limiting their ability to dephosphorylate PIP3 (14). If so, this mechanism may elucidate the inconsistency observed with USP10 in the PI3K pathway, wherein USP10 has been demonstrated to downregulate PI3K signaling (31). This point requires additional validation if USP10 or GSK3β inhibitors are to be used in the treatment of PI3K-mutant breast cancers. Nevertheless, we have conclusively demonstrated that genetic KO of USP10 or enzymatic inhibition of USP10 using a ubiquitin-binding variant decreases PI3K pathway activation in *PIK3CA*-mutant breast cancers.

The incorporation of comprehensive experimental investigations with mechanistic and clinical analyses has delineated a varied molecular landscape of PI3K resistance. Here, we identify a compensatory mechanism resulting in PI3Ki resistance that may explain the low response rate observed in the clinic. Furthermore, we describe a therapeutically actionable target, USP10, inhibitors to which may be used in the near term to circumvent PI3K resistance in cancer types harboring *PIK3CA* mutations.

## Methods

### Sex as a biological variant.

Only female mice were used in this study, reflecting the clinical relevance of breast cancer as a disease predominantly affecting female humans. This choice was guided by the need to model the hormonal and physiological environment most representative of human breast cancer, thereby enhancing the translational applicability of the findings.

### Statistics.

Data were analyzed using Prism, version 10.4.1 (GraphPad Software). Individual statistical analyses and methodology are detailed in the corresponding figure legends for figures presenting statistical comparisons and include 2-tailed *t* test and Dunnett’s multiple comparison test.

### Study approval.

Human breast cancer samples were obtained from the Pathology Department at the University Hospital Würzburg (Würzburg, Germany). Informed consent was obtained from all patients. Experiments agreed with the principles set out in the World Medical Association (WMA) Declaration of Helsinki and the Department of Health and Human Services Belmont Report. Use of samples was approved under University Hospital Würzburg Ethics Approval 17/01/2006. For PDX generation, we obtained fresh tumor samples from the Vall d′Hebron University Hospital and following the institutional guidelines. Informed written patient consent, approved by the Ethics Committee for Clinical Research and Animal Research of Vall d’Hebron Hospital [no. PR(AG)130/2015], was obtained for the use of these patient samples. All animal procedures were approved by the Ethics Committee of Animal Research of the Vall d′Hebron Institute of Oncology and by the Catalan Government (no. FUE-2020-01541918) and conformed to the principles of the WMA Declaration of Helsinki, the Department of Health and Human Services Belmont Report, and following the European Union’s animal care directive (no. 2010/63/EU).

### Data availability.

All data reported in this article will be shared upon reasonable request. RNA-Seq data generated in this study have been deposited in the National Center for Biotechnology Information Gene Expression Omnibus under accession number GSE302500. Values for all data points in the graphs are provided in the Supporting Data Values file. All remaining materials and methods are explained in Supplemental Methods in the Supplemental Data File.

## Author contributions

NK, SCEW, CMW, LM, MP, JLCR, CC, M Elkabets, MA, TK, M Eccles, AG, TW, JAN, NW, VV, AC, NV, S Bridgeman, S Bassi, AS, MH, PN, AR, MED, MR, JG, NP, OH, JC, SSS, NM, SJ, JR, DK, VS, and PJAE participated in experimental design, implementation, and interpretation of data. MA performed the molecular modeling. AG performed the bioinformatics analyses. MH, PN, and AR performed the scRNA analysis. JAN and SJ helped supervise experiments. JR was a medical consultant for the project. DK, VS, and PJAE conceived the project and contributed to interpretation of the results. PJAE and NK wrote the article.

## Funding support

- Curtin Research fellowship, grant CRF130006, to MA.
- Raine Medical Research Foundation, Raine Priming grant, to MA.
- Australian government, Pawsey Supercomputing Center, National Computational Merit Allocation Scheme for computational resources (pa6/pawsey0196, pawsey0361).
- Harry Perkins Institute of Medical Research, MACA Cancer 200 Ride funds, to TW and JAN.
- Agency for Management of University and Research Grants to LM and VS.
- European Social Fund, grants 2019FI_B_01199 and 2021 SGR 01510, to LM and VS.
- Curtin University start-up fund, Cancer Council Western Australia, grant CCWA 2022/1165, to PJAE.
- WA Near-Miss Awards, WANMA2021/7, to PJAE.
- Worldwide Cancer Research Grant (24-0136) to PJAE.
- Oncology One to DK and PJAE.

## Supplementary Material

Supplemental data

Unedited blot and gel images

Supporting data values

## Version 1 09/24/2025

In-Press Preview

## Version 2 11/17/2025

Electronic publication
