# Supplementary material for: USP10/GSK3β-mediated inhibition of PTEN drives resistance to PI3K inhibitors in breast cancer
Source: J Clin Invest. 2025 Sep 23;135(22):e180927. doi: 10.1172/JCI180927 (PMC12618074; doi:10.1172/JCI180927)
Supplement: Supplemental data [file jci-135-180927-s157.pdf]

Supplemental information for

Kumari N, et al. USP10/GSK3β-mediated inhibition of PTEN drives resistance to PI3K inhibitors in breast cancer.

This supplemental information contains:

Supplemental figures: 19

Supplemental Methods

Supplementary Figures

A

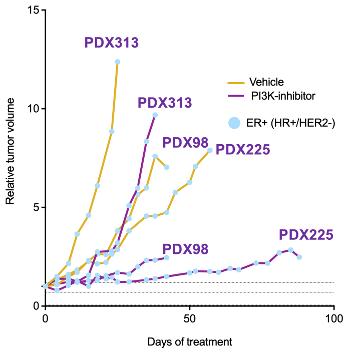

B

PDX	98	131	313	287	39	4	191	225	251	244
Subtype	ER+	ER+	ER+	ER+	ER+	ER+	ER+	ER+	ER+	ER+
PIK3CA	-	-	-	+	-	-	-	-	+	-
AKT	-	-	Mis	-	-	-	-	-	-	-
PTEN	-	-	-	-	-	-	-	-	-	Del
FGFR1	-	-	-	-	-	-	Amp	-	-	-

Supplementary Figure 1. PI3K inhibitor monotherapy in PI3K mutant breast cancers. (A) Spaghetti plot demonstrating relative tumour volume change in PDX313, PDX98, and PDX225 breast cancer patient derived xenografts treated with BYL719 75mg/kg (purple) or vehicle control (brown) for indicated time points. Blue symbols represent patient derived xenograft molecular subtype. (**B**) Table highlighting PDXO subtypes and relevant PI3K pathway mutations.

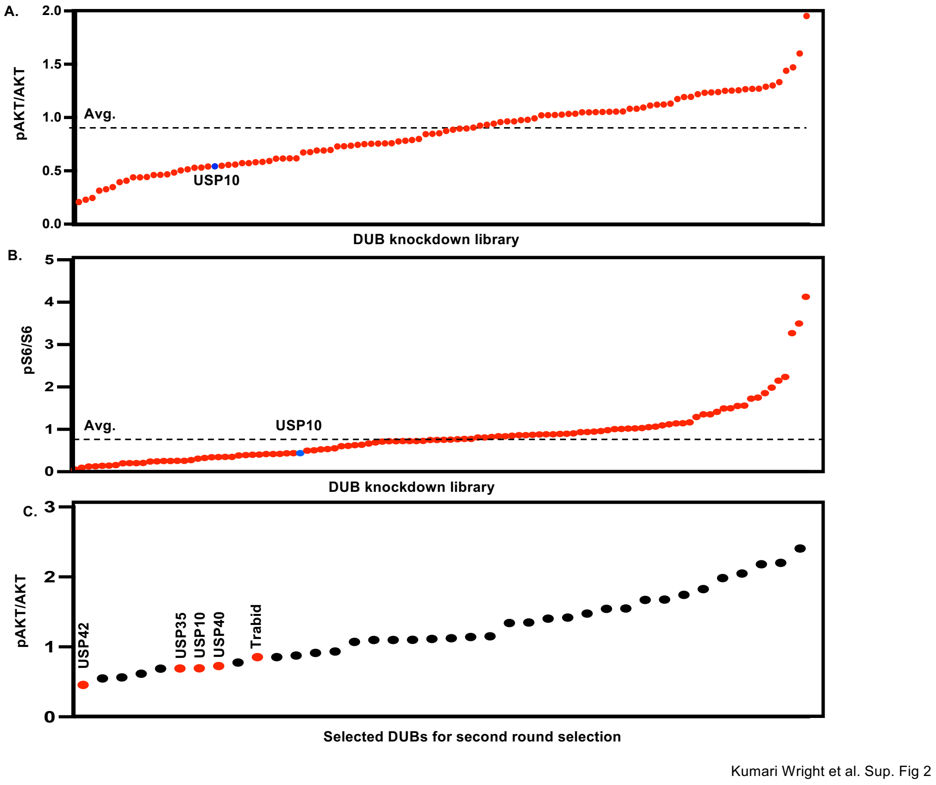

Supplementary Figure 2. DUB shRNA screen analysing pAKT and pS6. (A) Graph representing relative pAKT/AKT values obtained from the DUB shRNA screen done in duplicate. Images were quantified by Image J and average of both screens were plotted. (**B**) Graph representing relative pS6/S6 values obtained from the DUB shRNA screen done in duplicate. Images were quantified by Image J and average of both screens were plotted. (**C**) Graph representing relative pAKT/AKT values obtained from second round selection of DUB shRNA screen. Images were quantified by Image J and average of both screens were plotted.

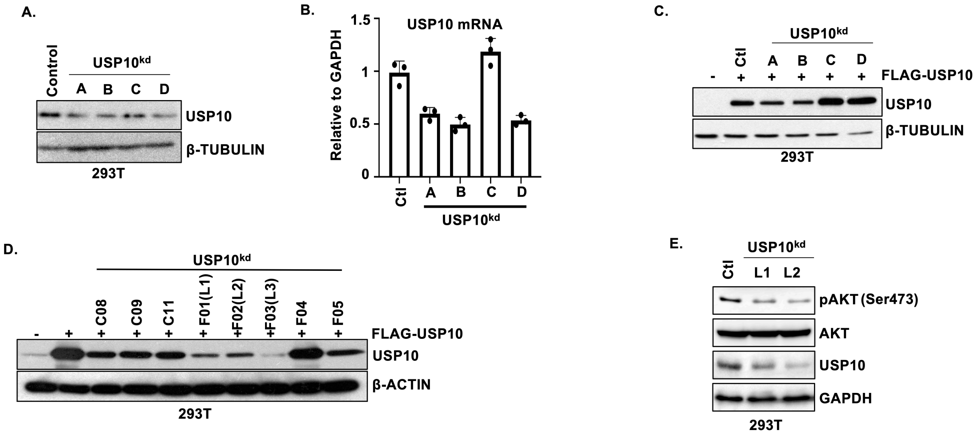

Supplementary Figure 3. USP10 regulates PI3K signalling. (A) Immunoblot analysis of HEK293T cells expressing shRNA vectors A, B, C, and D targeting USP10 from the DUB pool in the shRNA library and probed with the indicated antibodies. (**B**) qRT-PCR of *USP10* in HEK293T cells transfected as indicated. *GAPDH* is used as an internal control. (**C**) Immunoblot analysis of HEK293T cells transfected with FLAG-tagged USP10 and shRNA vectors A, B, C, and D targeting USP10 from the DUB pool and probed with the indicated antibodies. (**D**) Immunoblot analysis of HEK293T cells transfected with FLAG-tagged USP10 and various pLKO1-shRNA vectors targeting USP10 and probed with the indicated antibodies. pLKO1 constructs L1, L2, and L3 was used for all subsequent experiments. (**E**) Immunoblot analysis of HEK293T cells stably expressing shRNA vectors USP10-L1 and USP10-L2 and probed with the indicated antibodies.

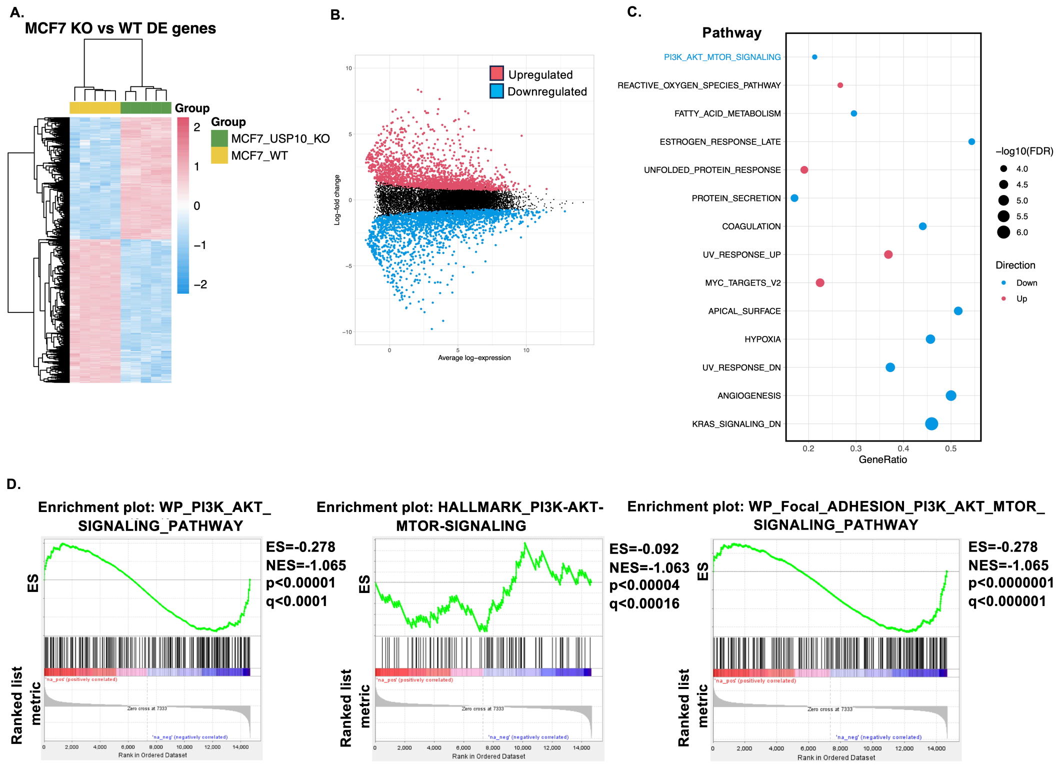

Supplementary Figure 4. USP10 deficiency upregulates PI3K signalling in MCF7 breast cancer cells.

(**A**) RNA sequencing (RNA-seq) analysing the indicated MCF7 cells or MCF7 USP10^{KO} cells and relative fold changes in gene expression (false discovery rate [FDR] < 5%) were visualized in a heatmap (*n* = 5). (**B**) Volcano plot of the DEGs. Expression of each gene is plotted as fold change (>1.5) of expression ratio concerning controls; upregulated and downregulated genes are represented by red and blue dots, respectively. (**C**) Enrichment analysis of genes significantly different (FDR <5%) between MCF7 cells and MCF7 USP10^{KO} cells from RNA-seq results. Ingenuity pathway analysis of pathways activated or inhibited in USP10^{KO} cells is shown. (**D**) Gene set enrichment analysis of (WP_Focal_ADHESION_PI3K_AKT_MTOR_SIGNALING_PATHWAY , WP_PI3K_AKT_SIGNAKING_PATHWAY, HALLMARK_PI3K_AKT_MTOR_SIGNALING) gene set signatures, extrapolated from the RNASeq analysis of MCF7 or MCF7-USP10KO1 cells (n=5). Enrichment scores (ESs), normalized enrichment scores (NESs), P values, and FDR are reported.

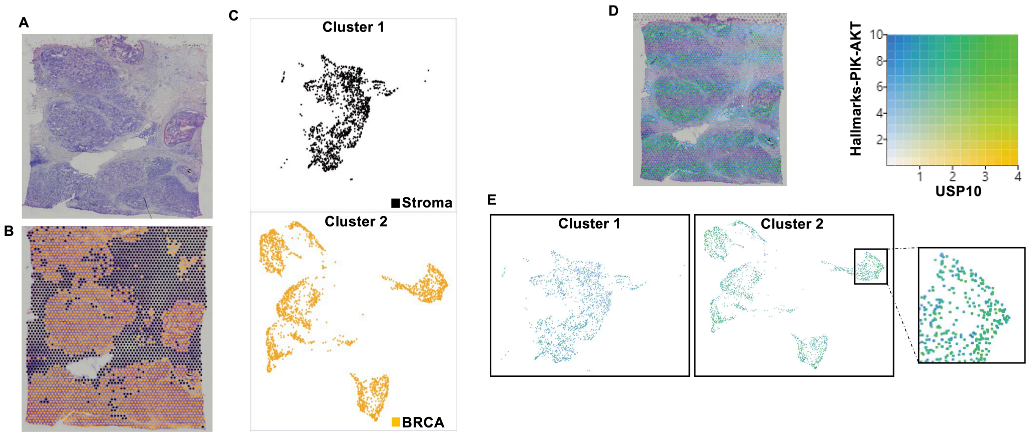

Supplementary Figure 5. USP10 expression correlates with PI3K upregulation in breast cancer.

(**A**) Overview image of human breast cancer FFPE sample used for spatial transcriptomic analysis (https://www.10xgenomics.com/datasets/human-breast-cancer-block-a-section-2-1-standard-1-1-0). Data visualized using the 10xGenomics Loupe browser (v8.0). (**B**) K means = 2 clustering of human breast cancer FFPE sample used to segment tumour (BRCA, orange) and stromal tissue (Stroma, black). Data visualized using the 10xGenomics Loupe browser (v8.0). (**C**) UMAP analysis of cell clustering of the 2 identified clusters showing a wide range of genetically defined subclones/differential gene expression of BRCA. Data visualized using the 10xGenomics Loupe browser (v8.0). (**D**) Correlation analysis of USP10 and genes of the PIK-AKT-Hallmark gene set on the breast cancer tissue section, derived from S5A. USP10 only expression areas are represented in yellow, PIK-AKT Hallmark gene set (HALLMARK_PI3K_AKT_MTOR_SIGNALING, M5923) only are marked in blue, co-expression is visualized in green. (**E**) Detailed insight into the occurring co-expression of USP10 and the PIK-AKT Hallmark gene set is a UMAP (c.) representation used. Zoom in shows the predominant co-expression of USP10 and PIK-AKT-Hallmark genes in BRCA. Data visualized using the 10xGenomics Loupe browser (v8.0).

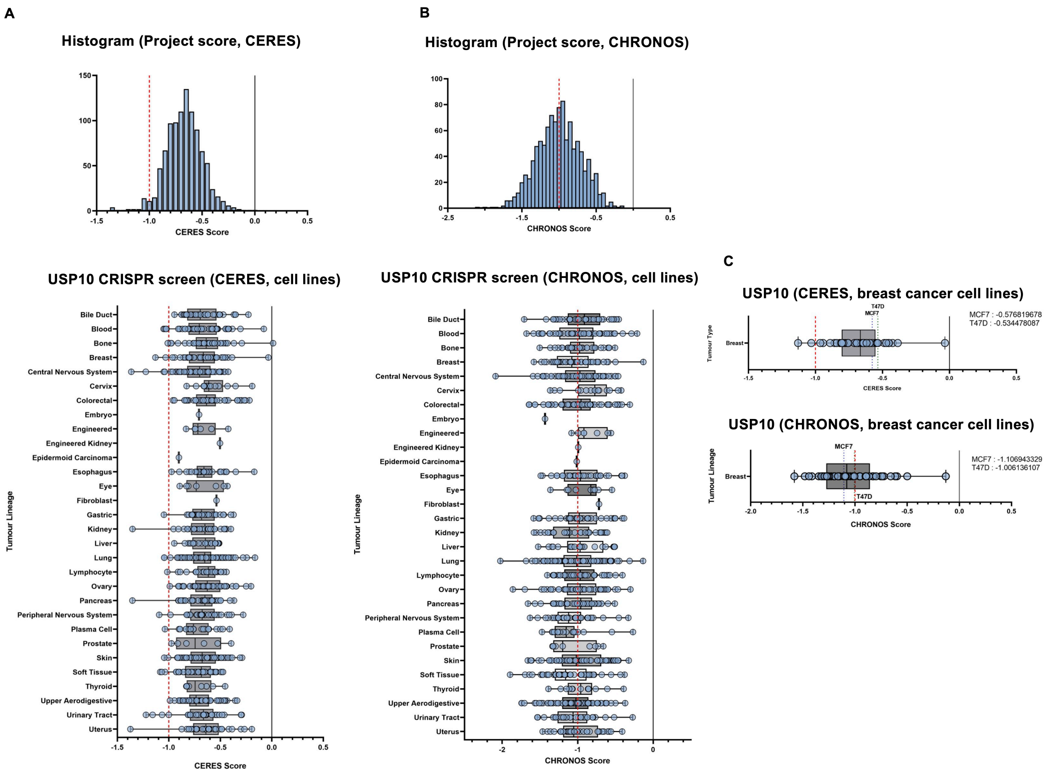

Supplementary Figure 6. CERES analysis of USP10 in cancer cell lines

(**A-C**) CERES (**A**) and CHRONOS (**B**) scores of USP10 from genome-wide CRISPR–Cas9 essentiality screens across 769 human cancer cell lines. The raw data were downloaded from DepMap (https://depmap.org/portal/). CERES and CHRONOS scores between 0 and -1 represent the median effects of nonessential genes and common core essential genes, respectively. The lower CERES or CHRONOS score indicates the higher cancer dependency of the specific gene with less than 0.5 classified as an essential gene. (**C**) CERES and CHRONOS scores of USP10 in breast cancer cell lines.

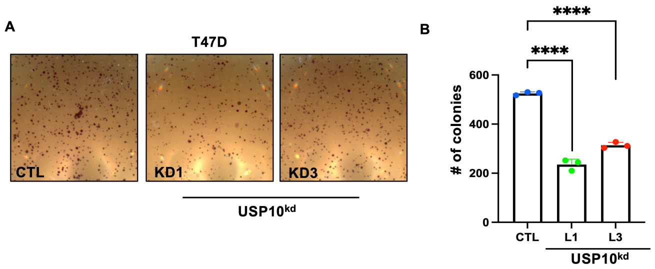

Supplementary Figure 7. USP10 depletion decreases proliferation (A-B) Representative images of 3D soft agar growth pictures (**A**) of T47D cells stably expressing shRNA vectors USP10L1 or USP10 L3 and quantification of colony numbers (**B**). Experiments are representative of three independent experiments. ****P ≤0.001 as determined by Student’s t-test.

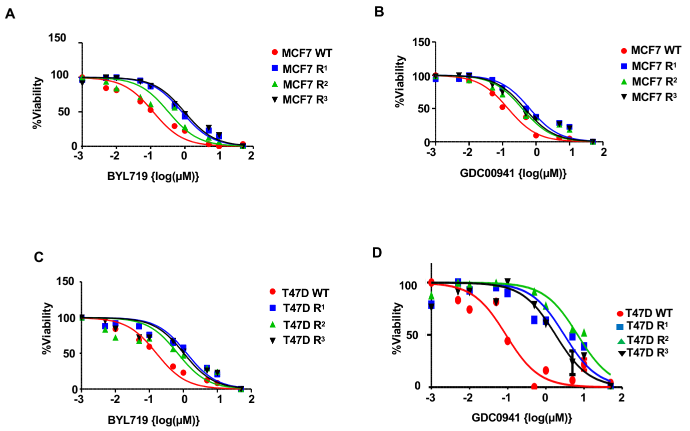

Supplementary Figure 8. Evaluation of MCF7 or T47D PI3K inhibitor resistant cell lines (A-D) Cells were treated with escalating doses of BYL719 or GDC0941, as indicated, for 72 hours. Viability was assayed using Cell-Titre Glo as described by the manufacturer. Data represent the mean of 5 replicates.

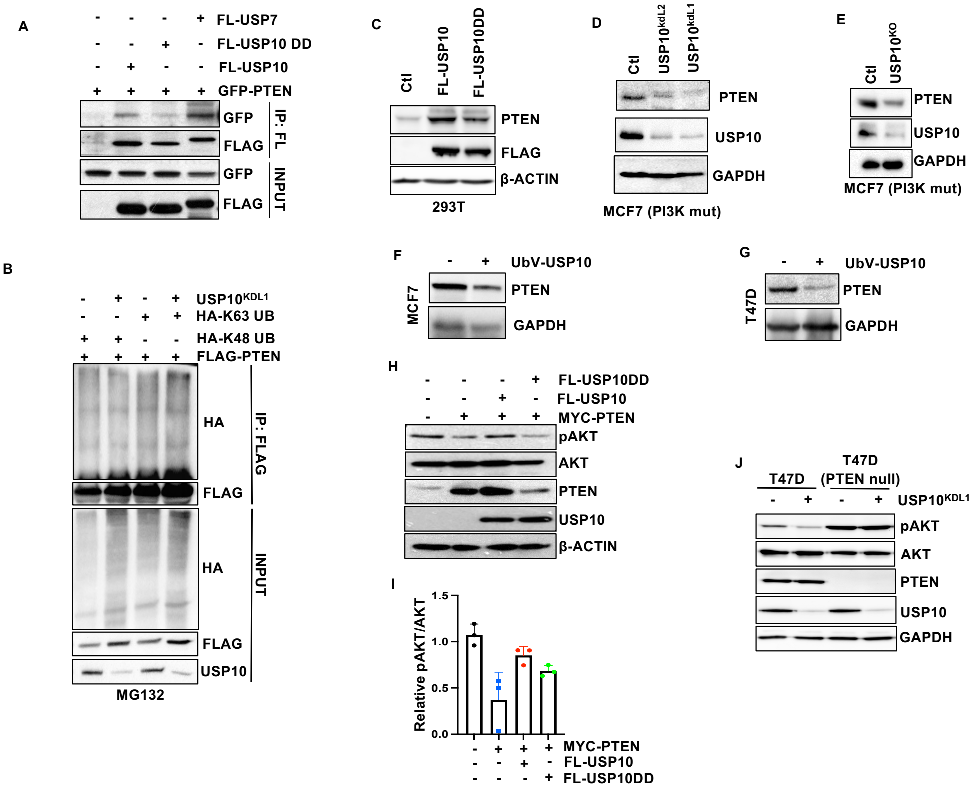

Supplementary Figure 9. USP10 does not affect K48 or K63 chain formation on PTEN **(A)** HEK293T cells were transfected as indicated. 48 hours later cells were lysed and immunoprecipitated with an anti-FLAG affinity resin. Immunoprecipitated lysates and whole cell extracts were probed with the indicated antibodies. (**B**) HEK293T cells transfected with FLAG-PTEN, HA-tagged ubiquitin K48 or K63 in the presence or absence of shRNA USP10KDL1. 72 hrs after transfection cells were treated with MG132 (5 µM) for 6 hrs. Lysates were immunoprecipitated with anti-FLAG affinity resin, resolved by SDS-PAGE and probed with indicated antibodies. (**C**) HEK293T cells were transfected as indicated. Whole cell extracts were probed with the indicated antibodies. (**D**) Immunoblot analysis of MCF7 cells stably expressing shRNA vectors L1 and L2 targeting USP10 and probed with the indicated antibodies. **(E**) Immunoblot analysis of MCF7 cells or MCF7 USP10 CRISPR knockout cells (USP10^{KO1}). Whole cell lysates were probed with the indicated antibodies. (**F-G**) MCF7 (**F**) or T47D (**G**) cells expressing UbV-USP10 or control. 24 hours after transfection lysates were probed with indicated antibodies. (**H**) HEK293T cells were transfected as indicated. Whole cell extracts were probed with the indicated antibodies. (**I**) Quantification of 3 independent experiments from (H). (**J**) Immunoblot analysis of T47D or T47D (PTEN null) cells stably expressing USP10 shRNA vector L1 and probed with the indicated antibodies.

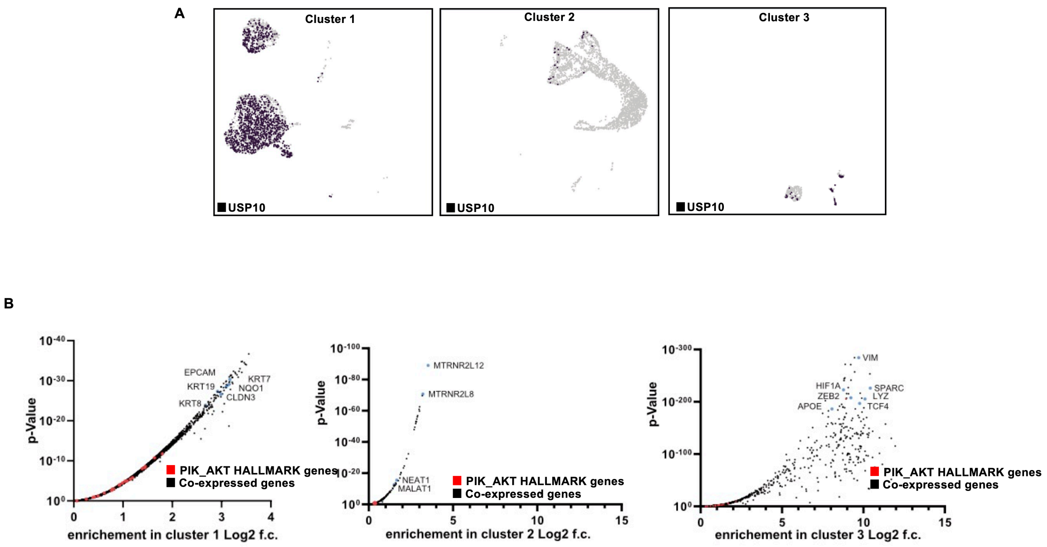

Supplementary Figure 10. USP10 correlates with PI3K activation in PTEN overexpressing cells.

(A) K means = 3 clustering of human invasive ductal carcinoma single cell sequencing data set (https://www.10xgenomics.com/datasets/7-5-k-sorted-cells-from-human-invasive-ductal-carcinoma-3-v-3-1-3-1-standard-6-0-0). USP10 expression in the different clusters is shown (black). Cluster 1 = cancer, cluster 2 = unidentified/mixed tissue, cluster 3 = stroma. **(B)** Volcano plots of upregulated genes in the identified clusters 1 to 3. Highlighted are cancer marker genes (cluster 1: EPCAM, KRT 7, 8, 19, NQO1 and CLDN3, respectively). Genes expressed in USP10 high cells were extracted by barcode filtering (1-4), followed by gene set analysis using the Loupe browser. Identified genes were presented with log2FC and p-Value, respectively. Volcano representation was visualized using GraphPad Prism 8. Highlighted in red are genes of the PIK-AKT-Hallmark gene set (HALLMARK_PI3K_AKT_MTOR_SIGNALING, M5923).

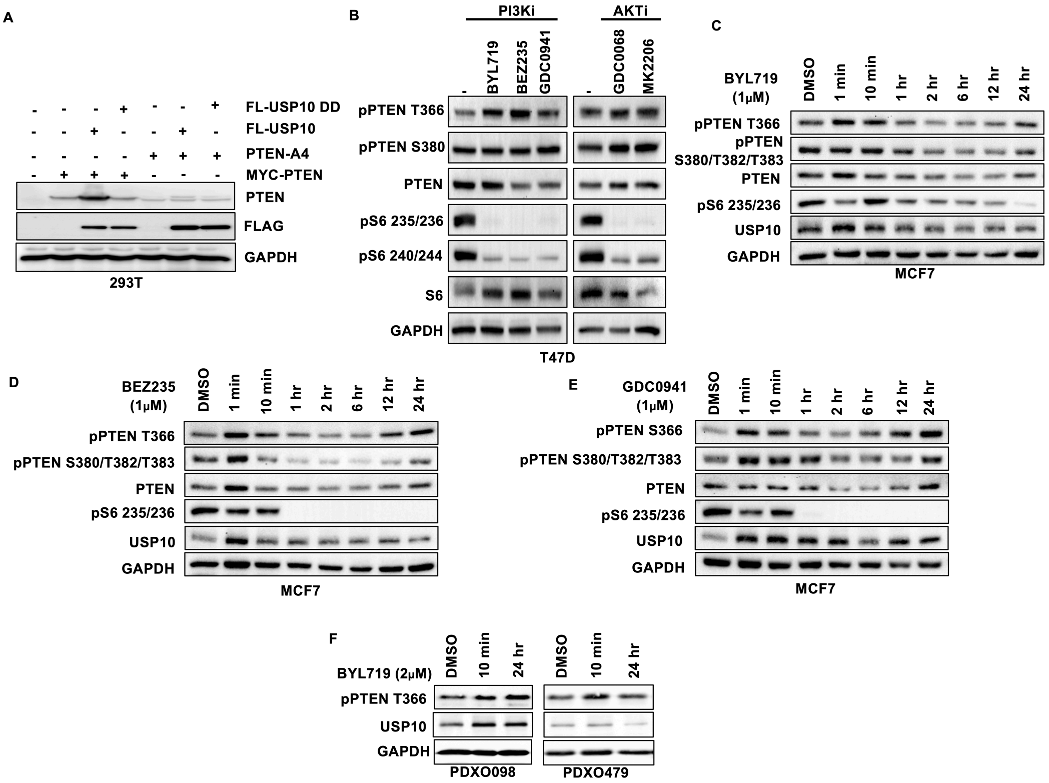

Supplementary Figure 11. PI3K inhibition alters PTEN tail phosphorylation (A) HEK293T cells were transfected as indicated. Whole cell extracts were probed with the indicated antibodies. (**B**) T47D cells treated for 24 hours with 1 μM of indicated PI3K inhibitors (PI3Ki) or AKT inhibitors (AKTi). Whole cell lysates were collected and probed with indicated antibodies. (**C**) MCF7 cells treated with BYL719 (1 μM) for the indicated time points. Lysates were collected and probed with the indicated antibodies. (**D**) MCF7 cells treated with BEZ235 (1 μM) for the indicated time points. Lysates were collected and probed with the indicated antibodies. (**E**) MCF7 cells treated with GDC0941 (1 μM) for the indicated time points. Lysates were collected and probed with the indicated antibodies. (**F**) PDXO098 tumours or PDXO479 tumours were treated with BYL719 (2 μM) for the indicated time points. Tumour lysates were collected and probed with the indicated antibodies.

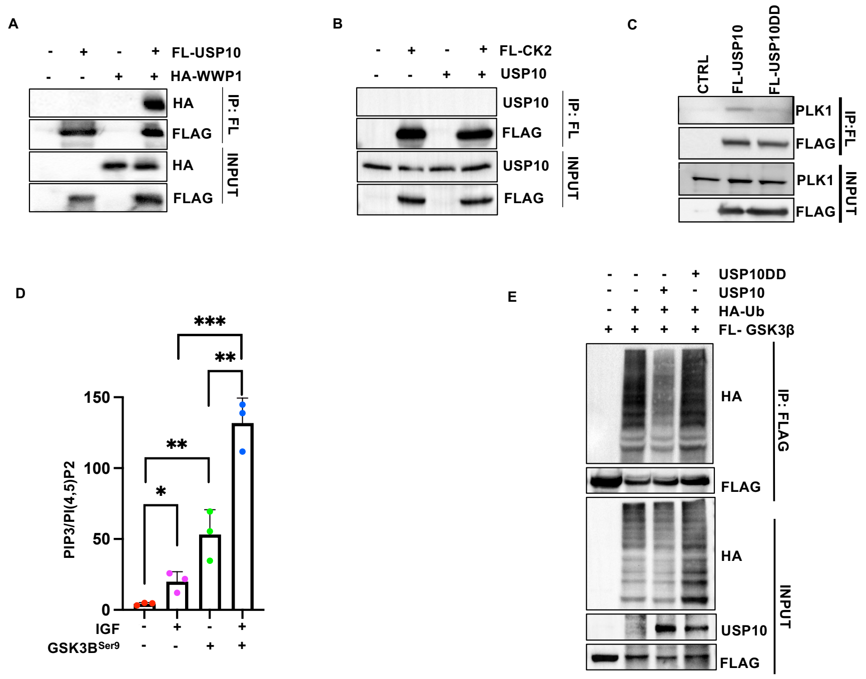

Supplementary Figure 12. USP10 deubiquitinates GSK3β (A-C) HEK293T cells were transfected as indicated. 48 hours later cells were lysed and immunoprecipitated with an anti-FLAG affinity resin. Immunoprecipitated lysates and whole cell extracts were probed with the indicated antibodies. (**D**) T47D cells were transfected as indicated. 48 hours later phospholipids were isolated from cells treated with DMSO or IGF (300 ng/ml) for 1 hr and relative PIP3 and PI(4,5)P2 levels were quantified by ELISA. Experiments are representative of three independent experiments. * P ≤0.05, **P ≤0.01, ***P ≤0.001 as determined by Student's t-test. (**E**) HEK293T cells transfected with FLAG-GSK3β, HA-tagged ubiquitin and CMV-USP10 or CMV-USP10DD. Lysates were immunoprecipitated with anti-FLAG affinity resin, resolved by SDS-PAGE and probed with indicated antibodies.

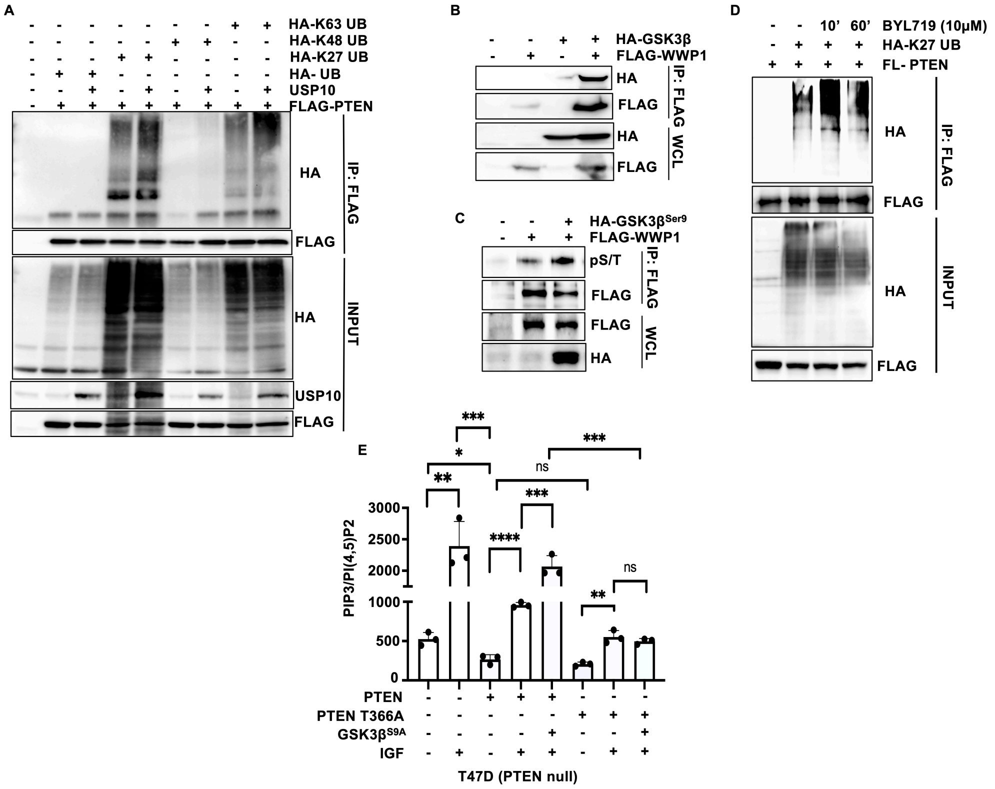

Supplementary Figure 13. USP10 regulates PTEN K27 ubiquitination potentially through WWP1. (**A**) HEK293T cells transfected with FLAG-PTEN, HA-tagged ubiquitin or its mutant isoforms K27, K48, K63 with or without CMV-USP10. Lysates were immunoprecipitated with anti-FLAG affinity resin, resolved by SDS-PAGE and probed with indicated antibodies. (**B**) HEK293T cells were transfected as indicated. Lysates were immunoprecipitated with anti-FLAG affinity resin, resolved by SDS-PAGE and probed with indicated antibodies. (**C**) MCF7 cells were transfected as indicated. Lysates were immunoprecipitated with anti-FLAG affinity resin, resolved by SDS-PAGE and probed with a phospho Serine/Threonine antibody, FLAG, or HA. (**D**) MCF7 cells transfected with FLAG-PTEN and HA-tagged K27 ubiquitin. After 48 hours cells were treated with 10 μM BYL719 as indicated. Lysates were immunoprecipitated with anti-FLAG antibody. Western blot was probed with indicated antibodies. (**E**) T47D-PTEN null cells were transfected as indicated. 48 hours later phospholipids were isolated from cells treated with DMSO or IGF (300 ng/ml) for 1 hr and relative PIP3 and PI(4,5)P2 levels were quantified by ELISA. Experiments are representative of three independent experiments. * P ≤0.05, **P ≤0.01, ***P ≤0.001, ****P ≤0.0001 as determined by Student's t-test.

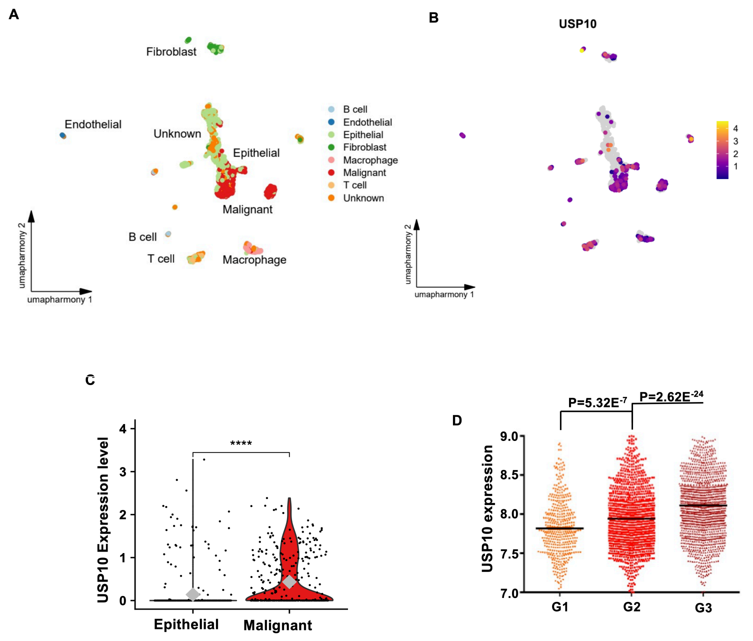

Supplementary Figure 14. USP10 is upregulated in malignant breast cancer tissues (A) A batch-corrected Uniform Manifold Approximation and Projection (UMAP), representing 1256 cells from triple-negative breast cancer (TNBC) patients. The cells were coloured to depict their respective cell type annotations. (**B**) A UMAP illustrating the normalized expression of USP10 across all cells. (**C**) A violin plot showing USP10 expression across epithelial and malignant cells extrapolated from figures A and B. The p-value for comparing USP10 expression was calculated using Wilcoxon test (**** = < 0.0001). Grey diamond represents the mean of each population. (**D**) Differential USP10 gene expression in different grades of breast cancer. Here, G2, G3 and G4 represents grade 2, grade 3, and grade 4 respectively. We conducted one-way ANOVA followed by posthoc tests using Bonferroni method, to calculate the corrected P-values for the differentially expressed genes in different grades of breast cancer.

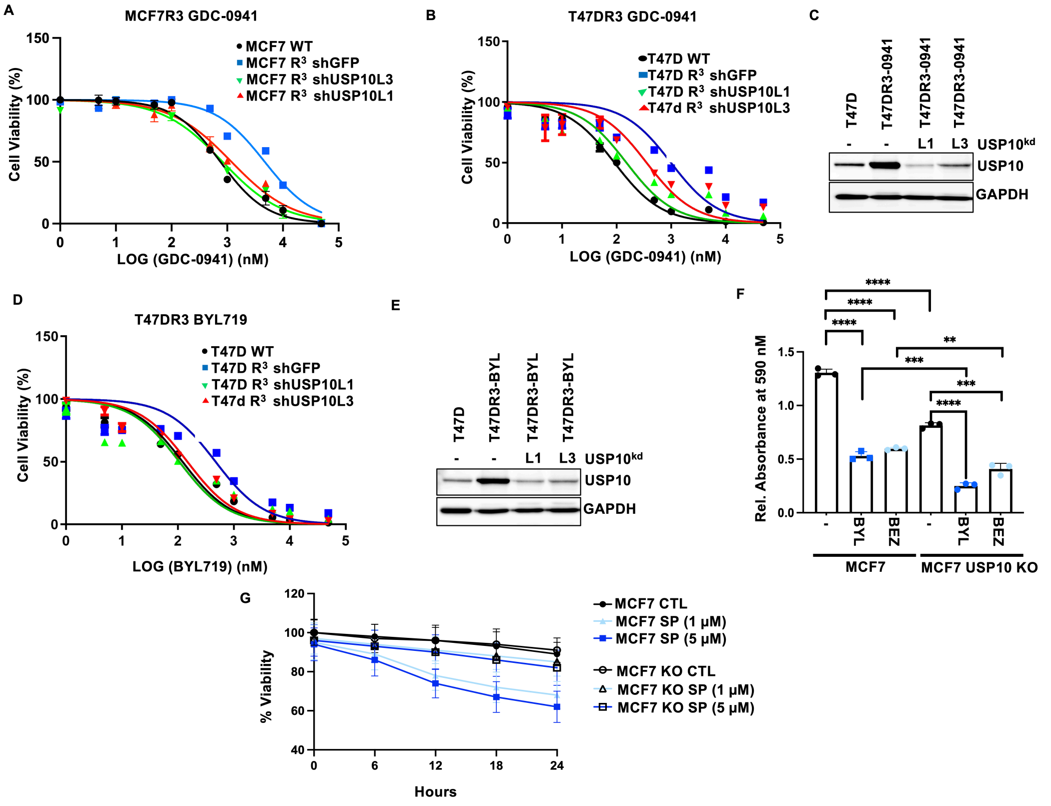

Supplementary Figure 15. USP10 depletion re-sensitizes PI3K inhibitor resistant cells to PI3K inhibitors

(**A**) MCF7 PI3K inhibitor resistant cells expressing shRNA USP10L1 or USP10 L3 or shGFP were treated with escalating doses of GDC0941, as indicated, for 72 hours. Viability was assayed using Cell-Titre Glo as described by the manufacturer. Data represent the mean of 5 replicates. (**B**) T47D PI3K inhibitor resistant cells expressing shRNA USP10L1 or USP10 L3 or shGFP were treated with escalating doses of GDC0941, as indicated, for 72 hours. Viability was assayed using Cell-Titre Glo as described by the manufacturer. Data represent the mean of 5 replicates. (**C**) Representative cell lysates probed as indicated from B. (**D**) T47D PI3K inhibitor resistant cells expressing shRNA USP10L1 or USP10 L3 or shGFP were treated with escalating doses of BYL-719, as indicated, for 72 hours. Viability was assayed using Cell-Titre Glo as described by the manufacturer. Data represent the mean of 5 replicates. (**E**) Representative cell lysates probed as indicated from D. (**F**) Quantification of colony formation assay of MCF7 cells or MCF7 USP10 knockout cells treated with either DMSO, 1 µM BYL719, or 1 µM BEZ235 for 10 days cultured in medium containing 10% FBS. The experiment was performed in triplicate $^{**}P \leq 0.01$, $^{***}P \leq 0.001$, $^{****}P \leq 0.0001$ as determined by Student's t-test. (**G**) Cell proliferation assay of MCF7 or MCF7 USP10 KO cells treated

with either DMSO or Spautin (1μM or 5μM) for indicated time points. Results are from three independent experiments.

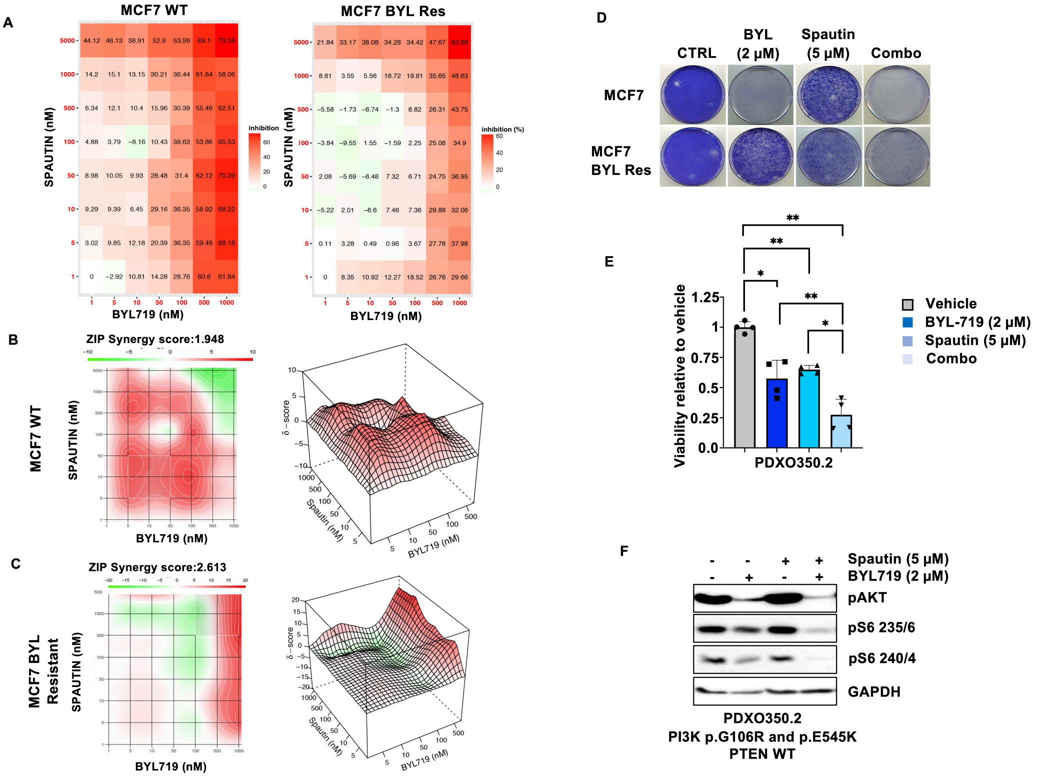

Supplementary Figure 16. Spautin-1 resensitises PI3K inhibitor resistant models to PI3K inhibition. (**A**) A dose matrix of BYL719 with Spautin-1 was created in parental and MCF7 BYL719 resistant cells. Viability was assessed after 5 days. Percent inhibition at each dose is presented. Synergy analysis of BYL719 and Spautin-1 in MCF7 cell line (**B**) and MCF7 BYL Res (**C**), showing the ZIP synergy score, as analysed using SynergyFinder software. (**D**) Colony formation assay of MCF7WT or MCF7-PI3K inhibitor resistant clone R3 stably treated with BYL719 (2 μM), Spautin-1 (5 μM), or in combination for 21 days. (**E**) Viability assay of PDXO350.2 treated with BYL719 (2 μM), Spautin-1 (5 μM) or the combination for 5 days. p = two tailed t test, *P<0.05, ** P<0.01. (**F**) Immunoblot analysis of PDX350.2 treated in D. Whole cell extracts were probed with the indicated antibodies.

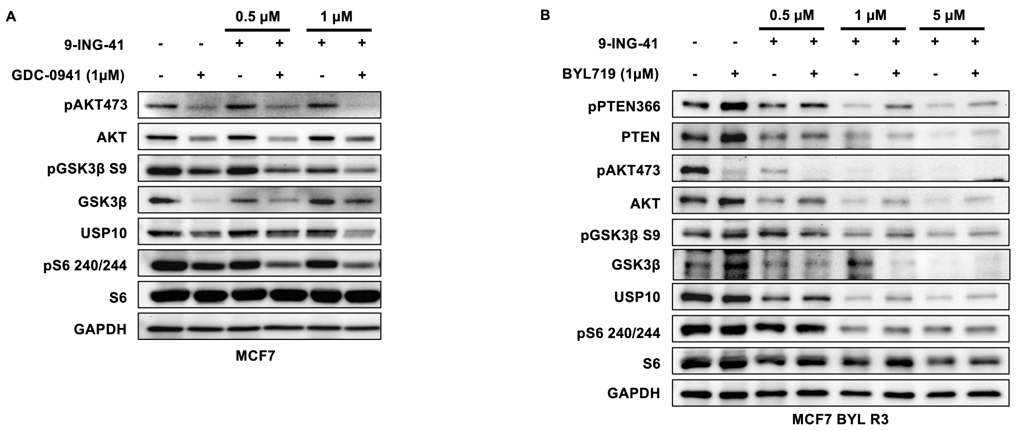

Supplementary Figure 17. GSK3β inhibition further downregulates PI3K signalling in PI3K inhibitor resistant cells (A) MCF7 cells treated as indicated for 48 hours. Treated lysates were resolved by SDS-Page and probed with indicated antibodies. (**B**) MCF7 BYL719 resistant cells treated as indicated for 48 hours. Treated lysates were resolved by SDS-Page and probed with indicated antibodies.

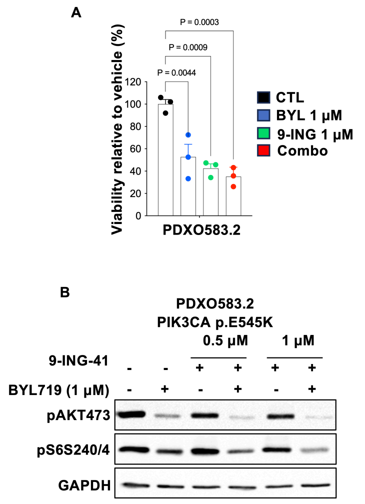

Supplementary Figure 18. GSK3β inhibition enhances PI3K inhibition (**A**) Viability assay of PDXO583.2 treated with BYL719 (1 µM), 9-ING-41 (1 µM) or the combination for 5 days. (**B**) Immunoblot analysis of PDXO583.2 treated in A. Whole cell extracts were probed with the indicated antibodies. Experiments were performed in triplicate; P values were calculated using Tukey's test.

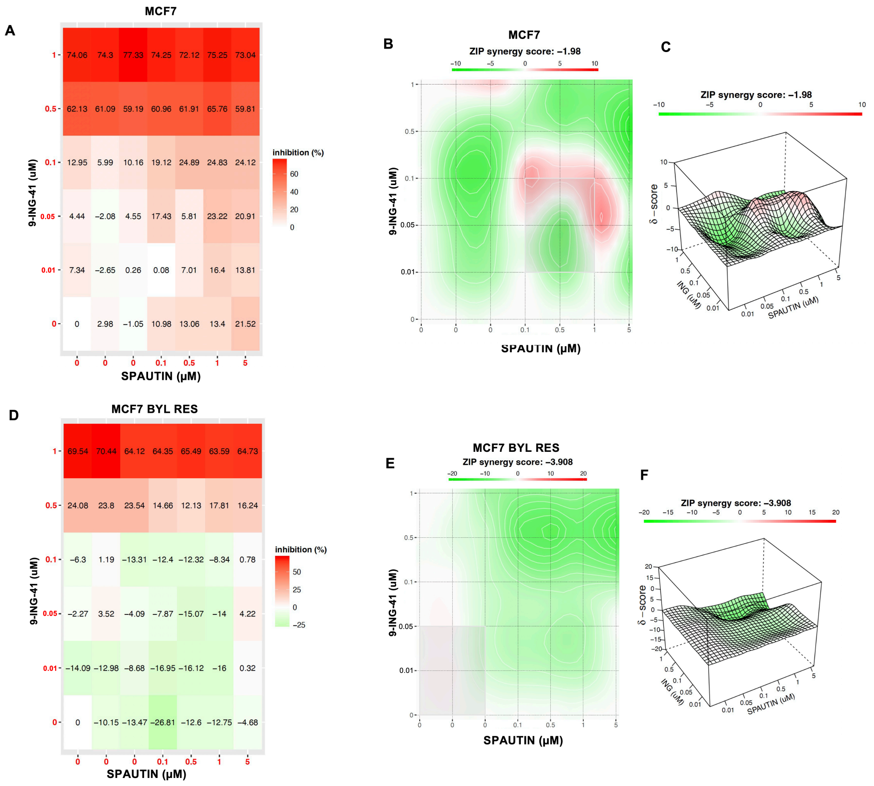

Supplementary Figure 19. GSK3β inhibition is antagonistic with USP10 inhibition (A-F) A dose matrix of 9-ING-41 with Spautin-1 was created in parental (**A**) and MCF7 BYL719 resistant cells (**D**). Viability was assessed after 5 days. Percent inhibition at each dose is presented. Synergy analysis of BYL719 and Spautin-1 in MCF7 cell line (**B-C**) and MCF7 BYL Res (**E-F**), showing the ZIP synergy score, as analysed using SynergyFinder software.

Supplemental Methods

Molecular modelling

The PTEN sequence was obtained from UniProt (accession number P60484). To generate a full-length PTEN model, the sequence was submitted to the I-TASSER server (1). Five models were obtained, with the top scoring of these used in the subsequent steps. Prior to dimeric assembly, the structure was N-terminal acetylated using the Protein Preparation Wizard in Maestro (Schrodinger), then manually reviewed and adjusted to remove erroneous *cis* amides with the aid of the Protein Reports tool in Maestro. The adjusted structure was then subject to energy minimisation using Prime (Schrodinger).

To assist in generating a PTEN dimer structure, the Protein Data Bank was searched to identify structures of proteins carrying PTEN C2 domain folds (PFAM accession number PF10409) crystallised in a dimeric configuration. One structure was identified, that of the *Ciona intestinalis* voltage sensor-containing phosphatase (Ci-VSP) (PDB 3V0D); (2) the dimeric configuration in this structure is strongly reminiscent of the PTEN dimer structure predicted using small angle X-ray structure (3). Two copies of the full-length PTEN model were aligned to each monomer of Ci-VSP. Residues within 6.0 Å of the dimer interface were subject to side-chain resampling using Prime, followed by energy minimisation of these residues, finally followed by energy minimisation of the complete dimer.

Phosphorylated PTEN structures were generated by introducing the relevant mutations at the relevant sites in both chains using Maestro. Residues within 6.0 Å of the newly created phosphosites were then subject to side-chain resampling and energy minimisation using Prime.

Systems were parameterised in AmberTools 14 (4) using the AMBER *ff14SB* forcefield, (5) which also includes parameters for phosphorylated amino acids.(6, 7) The generated topology was converted to GROMACS format using *acpype*, (8) with subsequent preparation and simulation performed using GROMACS 2018.3 (9).

Structures were solvated in TIP3P water (10) in a dodecahedral box with a minimum distance of 10 Å from complex to box edge. The system was charge-neutralised by the addition of sodium and chloride ions, with NaCl added to a concentration of 0.1 M. The resulting system was energy-minimised, then equilibrated in NVT and NPT ensembles for 0.1 ns each with harmonic position restraints on heavy atoms at 1,000 kJ/(mol nm^2). Production simulations were performed without harmonic position restraints for 50 ns with a target temperature of 310 K and pressure of 1 atm. Temperature coupling employed the use of the Berendsen Thermostat

with velocity rescaling (*V-rescale*).(11) Pressure coupling employed the use of the Berendsen barostat (12). Long-range electrostatic interactions were calculated using the Particle Mesh Ewald method (13). All covalent bonds were constrained using the linear constraints solver (LINCS) (14). Atomic coordinates, velocities and energies were saved every 10 ps. Time steps of 2 fs were employed throughout. Up to five simulations were performed for each complex, with the three most stable, as judged by catalytic domain heavy atom RMSD over time, selected for binding energy calculations.

Binding energy calculations were performed using the molecular mechanics-generalised Born/surface area (MM-GB/SA) approach, implemented by the *MMPBSA.py* tool of AmberTools (15). The Onufriev-Bashford-Case GB model (*igb* = 5) was used to calculate polar-desolvation energy (16). The non-polar desolvation energy was estimated by determining changes in solvent-accessible surface area using the linear combinations of pairwise overlaps (LCPO) method (17).

Quantitative Real Time Polymerase Chain Reaction

Cells were collected, washed twice in PBS and subsequently lysed for RNA extraction according to the Thermo Fisher Scientific GeneJET RNA purification kit protocol (#KO731). Transcriptor First Strand cDNA Synthesis protocol (#0 4379012 001) was used for the conversion of cDNA from mRNA. In order to assess the relative amounts of mRNA of USP10, the Applied Biosystems Power SYBR Green PCR Master Mix (#4367659) was used along with the appropriate primers. Reactions were carried out on an ABI 7500 FAST instrument. Relative mRNA values are calculated by the ΔΔCt method. GAPDH as the housekeeping gene was used as a control. Primers for qRT-PCR were designed and purchased from IDT. The following QRT primers were used: USP10-5"-GAT CCT GTA GCC ATA AAG ATT GC -3", 5"-GGT TGC AAC GAC ACT GGT T -3"; GAPDH-5"- CAT ACC AGG AAA TGA GCT TGA C-3", 5"-AAC AGC GAC ACC CAC TCC TC -3"

Cell culture, transfection, and immunoblotting

HEK293T and breast cancer cell lines MCF7 and T47D were cultured in 10 cm dishes in Dulbecco’s Modified Eagle medium (Gibco) or RPMI 1640 with glutaMAX medium (Gibco) supplemented with 10% FBS and 1% Penicillin/Streptomycin at 37 ºC and 5% CO2 atmosphere. To ensure against genetic drift cell lines were used for a maximum of 15 passages upon receipt from ATCC. Transient transfections were carried out using the calcium phosphate transfection methods. The cells were seeded (1.2×10^6/ 10 cm plate) one day before transfection.

20 μg of shRNA vectors, 1 μg of CMV-GFP, 10 μg of overexpression constructs, 2.5M CaCl2 (50 μl), 1x HBS (950 μl) were incubated for 16 hours then washed with 1X PBS and replaced with fresh media. Cells were lysed using RIPA buffer (50 mM Tris-HCl pH 7.4, 150 mM NaCl, 1% Nonidet P-40, 0.1% SDS and 0.5% sodium deoxycholate) supplemented with Protease inhibitor cocktail (Complete EDTA-free tablet, Roche) and phosphatase inhibitors (50 mM sodium fluoride, 10 mM b-glycerophosphate, 1 mM magnesium chloride and 1 mM sodium orthovanadate) at different timings depending on the nature of experiment. For ectopic expression, cells were lysed after 48 hours whereas it was 72 hours for shRNA knockdown. For Insulin Growth factor stimulation (IGF stimulation) cells were serum starved for 12 hours Cells were then stimulated for different time points using 300 ng of IGF-1/1ml of serum free media. Cells were immediately lysed after stimulation for required time periods. Whole cell extracts were separated by SDS-Polyacrylamide gel electrophoresis (PAGE, 10% gel) and transferred to PVDF membranes using the wet transfer method. Membranes were blocked using bovine serum albumin 5% in PBS-Tween (1%) over night at 4°C. Specific primary antibodies (1:1000) against protein of interest and secondary antibodies like ECL-anti-rabbit and ECL-anti-mouse (1:10,000) conjugated to HRP were used for immunoblotting. Pierce ECL WB substrate (Thermos Sc.) or Amersham ECL Prime Western Blotting Detection Reagent (Cytiva) were used for detection. Band intensities were quantified using Image J.

Immunoprecipitation

Optimized ELB buffer containing 250 mM NACL, 0.5% Nonidet p-40, 50 mM HEPES, Ph 7.3 supplemented with protease inhibitor was used for cell lysis. FLAG-conjugated beads, Protein A or Protein G beads (GE Healthcare) were used based on the nature of protein being immunoprecipitated. Approximately 500 μg-1mg of lysate was utilised for overnight incubation (4 °C) with the desired antibody. Next day, beads were washed 5 times in lysis buffer. Beads were boiled in 2X SDS- sample buffer (with 10% β-mercaptoethanol) and run on a polyacrylamide gel, along with the corresponding whole cell lysate.

Transfection of RNA

HEK 293T cells were sub-cultured the day prior to performing the transfections to achieve the number of cultures required at a confluency of 60-80%. Just prior to setting up the transfection reaction the cells had their media removed via vacuum aspiration and were washed once with PBS before being resupplied with fresh DMEM. The desired amounts of purified DNA/RNA

products were prepared in a 1.5 ml tube alongside separate tubes containing Lipofectamine™ 2000 Transfection Reagent at a ratio of 1:3 per ug of nucleic acid. To each tube, 200 µL of Opti-MEM™ I Reduced Serum Medium was then added before the lipofectamine containing solution was added to the respective nucleic acid tubes with pipetting to mix and introduce aeration before being left for 20- 30 mins. 600 µL of fresh DMEM was then added to each tube before the entire reaction volume was placed on cells in a drop-wise fashion. The cells were left to incubate at 37 °C in a humidified incubator with 5% CO2 before being washed twice in PBS and supplied with fresh medium 4-6 hrs post-transfection. The cells were finally harvested at 24 hrs post-transfection.

Expression plasmids and antibodies

The expression plasmids pCDNA3-GFP-PTEN, pCMV- FLAG WT-PTEN, MYC-PTEN, PCI-neo FLAG-HAUSP, FLAG-USP13, pRK-HA-ubiquitin and all ubiquitin mutant subvariants were purchased from Addgene. pCMV-FLAG-USP10 and pCMV-USP10 was purchased from the MRC unit at the University of Dundee. The catalytically inactive USP10 was made using site-directed mutagenesis kit (Agilent) and confirmed by DNA sequencing. Previously verified short-hairpin sequences against USP10 were cloned into pRetroSuper and confirmed by DNA sequencing. Sequences are described in Brummelkamp et al. (18). pLenti-shGFP and pLenti-USP10 constructs were previously generated in our lab. shUSP10-L1: CGA CAA GCT CTT GGA GAT AAA. shUSP10-L2: CGA CAA GCT CTT GGA GAT AAA. shUSP10-L3: CCT ATG TGG AAA CTA AGT ATT. USP10 CRISPR guide RNA cloned into pLentiCRISPR v2 was purchased from GenScript. The following USP10 guide RNA sequences were used G1: CAGGAGTCTGTAAATATATG, G2: CGGCGGCTACCTGCGGGCTG (Used in all derived clones), G3: CATGGCCCTCCACAGCCCGC, G4: CTTACCTCAACTGAAGATCG, G5: GACTCCTCGATCTTCAGTTG, G6: GTGCAGCTTCCTCCATACAG. Specific primary antibodies against pAKT (S473; #9271) and (S308; #9275), pS6 (235/236; #4858), pS6 (240/244; #2215), S6 (#2217), β-Tubulin (#2146), GAPDH (#2118), AKT (#9272), pPTEN S380 (#9549), PTEN (#9559), GFP (#2956), (Cell Signaling) , PTEN (Santa-Cruz; sc-7974), USP10 (Bethyl; A300-900A), FLAG (Sigma; F7425), HA (Y11, Santa-Cruz, sc-57592), pPTEN T366 (Abcam-ab109454) and Beta-Actin (Santa Cruz, sc-47778). All primary antibodies were used at 1:1000 dilution. Secondary antibodies IgG-anti-rabbit and IgG-anti-

mouse conjugated to HRP were purchased from Cell Signalling or Amersham.

Cell Viability, Coomassie staining, and dose matrix analysis.

Cells were seeded in 96 well plates (500 cells/well). Drugs were added next day and assayed using CellTiter-Glo (Promega) according to manufacturer protocols after three to five days. Growth curves performed twice with 5 replicates each. For dose-matrix analysis growth curves were performed in triplicate and combined data was analysed using synergyfinder.fimm.fi. For Coomassie staining, cells were seeded in 10 cm culture dish (50000 cells). Cells were treated with BYL719 (2 µM) or Spautin-1 (5 µM) every three days until end point was reached. Once cells reached confluency cells were washed with phosphate-buffered saline (PBS), fixed in 3:1 Methanol:Acetic acid for 10 minutes then stained with Commassie solution prepared in 25% methanol for 1 hour. Afterwards cells were washed in water three times and air-dried.

Lentiviral expression and CRISPR clone generation

To produce stable T47D, MCF7 and resistant cell lines, cells were transfected with pLenti-GFP, shUSP10#L1, shUSP10#L2, and shUSP10#L3 (Figure 3D) along with lentiviral packaging constructs (pCMV-VSVG, pMDLg-RRE and pRSV-REV). The media was collected 24 hours post transfection and passed through a 0.45µm filter, prior to use. T47D and MCF7 cells were infected with the supernatants in the presence of Polybrene (0.01%). The cells were selected and maintained in Puromycin (1.5 µg/10 ml) containing media until parental cell line was no longer viable. Similarly, pLentiCRISPR v2 containing desired USP10 guide RNA were co-transfected with the above mentioned lentiviral packaging constructs. Viral media was incubated on desired cell line with Polybrene (0.01%). After 48 cells were selected with puromycin and single clones were selected by serial dilution. Positive clones were validated by western blot.

UbVS assay

HEK293T WT cells were lysed in 1% v/v NP-40, 10% v/v glycerol, 2% v/v $NaVO_3$ supplemented with Complete Protease Inhibitor Cocktail (Roche 11697498001). Lysates were incubated for 1 hr at 4°C with rotation, then centrifuged to clarify the lysate, and the supernatant collected and stored at -20°C. Total protein concentration was determined using Pierce Micro BCA Protein Assay Kit (ThermoFisher 23235). 0.5 µg/µl cell lysates, supplemented with 1mM DTT were incubated with 50 µM UbV10 compound or equivalent volume of DMSO vehicle

control and incubated at 23 °C for 15 min with vigorous shaking. After which 0.2 µM UbVS or equivalent volume of lysis buffer vehicle control was added. The samples were further incubated at 23 °C for 5 min with vigorous shaking. Reactions were immediately quenched with 5 µl (per 20 µl reaction) of lithium dodecyl sulfate 4x buffer (ThermoFisher B0007) with 10% 2-Mercaptoethanol. Samples were vortexed vigorously followed by incubation at 95 °C for 5 min. 20 µl of reaction was loaded onto Bolt™ Bis-Tris Plus Mini Protein Gels, 4-12% (Thermo NW04120BOX) and electrophoresed at 150 V for approximately 6 hours in 1x MES buffer, with electrophoresis tank kept at 4°C throughout. Proteins were transferred to PVDF membrane overnight, as above, using 150 mA at 4°C. Membranes were blocked and probed for USP10 as per above immunoblotting methods.

Generation of PDXs

Generation of PDX models was previously described in Palafox et al. (19). In short, tumour pieces of 30–60 mm^3 obtained from breast cancer patient primary tumours or metastatic lesions at time of biopsy were immediately implanted into the mammary fat pad (surgery samples) or the lower flank (metastatic samples) of 6-week-old female NOD.Cg-PrkdcscidIl2rgtm1Wjl/SzJ mice. Mice were continuously supplemented with 1 µmol/L 17β-estradiol (Sigma-Aldrich) in their drinking water. Upon tumour growth of the engrafted tumours, a tumour piece was implanted into the lower flanks of new recipient mice for the model perpetuation. For molecular characterisation of PDXs flash-frozen and formalin-fixed paraffin embedded (FFPE) samples were taken. Staining of estrogen receptor (ER), progesterone receptor (PR) and human epidermal growth factor receptor 2 (HER2) were undertaken following the protocol provided by Ventana Medical Systems, Inc as described in (19).

Sex as a biological variant

Only female mice were used in this study, reflecting the clinical relevance of breast cancer as a predominantly female disease. This choice was guided by the need to model the hormonal and physiological environment most representative of human breast cancer, thereby enhancing the translational applicability of the findings.

In vivo experiments

To assess the sensitivity to various targeted therapies, each patient-derived xenograft (PDX) was subcutaneously implanted into six-week-old female athymic nude HsdCpb:NMRI-Foxn1nu mice (M. musculus, Janvier) or NOD.Cg-PrkdcscidIl2rgtm1Wjl/SzJ mice (M. musculus, Charles Rives). The mice received 1 µmol/L 17β-estradiol (Sigma-Aldrich) in their drinking water and were housed at 18–23 °C with 40–60% humidity, following a 12-hour light/12-hour dark cycle. Upon xenograft growth, tumor-bearing mice were randomly assigned to treatment groups, with tumors ranging from 100 to 300 mm3 for drug efficacy experiments. BYL719 was administered via oral gavage once daily, six days a week, at a dose of 35 mg/kg, dissolved in 0.5% methyl cellulose-distilled water. GDC-0032 was administered via oral gavage once daily, six days a week, at a dose of 25 mg/kg, dissolved in 0.5% methyl cellulose and 0.2% Tween 80 in distilled water. Tumor growth was measured bi-weekly, blinded to the treatment effect, with caliper measurements starting on the first day of treatment and continuing for 35 days. Mouse weights were recorded twice weekly. Tumor volume was calculated using the ellipsoid formula: V = (length × width^2) × (π/6). Mice were euthanized when tumors reached 1500 mm3 or in the case of severe weight loss, following institutional guidelines. All efficacy experiments included an untreated control arm with a percentage change in tumor growth exceeding 20% from the initial volume. Antitumor activity was determined by comparing the tumor volume on the last day of treatment to its baseline (day 1): % tumor volume change = (V35days – Vbaseline)/Vbaseline × 100. The antitumor response was classified based on the relative change in tumor volume upon treatment, similar to the Response Evaluation Criteria in Solid Tumors (RECIST) and labeled as mRECIST. Complete response (CR) was defined as the best response ≤ -95%; partial response (PR) as -95% < best response ≤ -30%; stable disease (SD) as -30% < best response ≤ +20%; and progressive disease (PD) as the best response > +20%. Models showing preclinical benefit from BYL719 (SD, PR, and CR) were categorized as BYL719-sensitive. At the end of the experiment, animals were euthanized using CO2 inhalation, and tumor volumes were plotted as mean values ± SEM.

Synergy test

Cells were plated in 96-well formats and exposed to escalating doses of test drugs (BYL719: 0–10 µM ; Spautin: 0–2 µM), with a growth period of four days. Cell proliferation in various treatment groups was quantified as a percentage relative to control (DMSO-treated) cells, calculating growth inhibition accordingly. The Synergyfinder website was employed for computing ZIP Synergy scores.

Generation of clean PCR product for IVT

The genes encoding for the UBVs were amplified from DNA vectors via PCR. The primers sequences are as follows: T7-FLAG-UBV10-sense: TAATACGACTCACTATAGGGG ATACCATGGATTACAAGGATGATGATGATAAGGATTACAAGGATGATGATGATA AGCATATTTTCGTGGAAACACC and UbV10-as: ATGGTCTAGAAAGCTTCATAAATTCTTG GCAAG . A 300bp product was obtained for USP10 UbV. PCR product was pooled for each gene and purified via the MACHEREY-NAGEL NucleoSpin® TriPrep, Mini kit for RNA, DNA, and protein purification, following the manufacture's instructions to obtain a concentration of at least 160 ng/µL.

In-vitro transcription (IVT)

The T7 mScript™ Standard mRNA Production System by CELLSCRIPT was utilised for the production of capped and tailed mRNA for transfection into eukaryotic cell lines. The manufacture's guidelines were followed with some exceptions detailed here. The Clean DNA template was diluted to 160 ng/µL in RNase-Free water before performing the standard mScript T7 IVT reaction as described in the procedure provided by CELLSCRIPT; however, the incubation period was extended to 1 hr at 37°C to achieve a greater yield of final product. The IVT reaction was followed by a DNase1 treatment for 20 minutes at 37°C to remove the template DNA. The remaining RNA was then purified using the MACHEREY-NAGEL NucleoSpin® RNA Clean-up kit as per manufacture's instructions.

Prior to the synthesis of capped RNA the purified product was taken up to a final volume of 72 µL with RNase-Free water before being heat denatured for 5 mins at 65°C and then placed on ice as reagents were prepared for the capping reaction. The capping reaction was performed as per the instructions provided by CELLSCRIPT, while notably the incubation period was increased to 2 hrs as recommended for RNA products <730 nucleotides in length to aid in 2'-O-methylation.

The capped RNA proceeded directly into the 3'-Poly(A)-Tailing. This reaction was set up as per the provided instructions while yet again the incubation period was extended for up to 3 hrs. The now capped and tailed mRNA was again purified as previously mentioned utilising the MACHEREY-NAGEL NucleoSpin® RNA Clean-up kit.

1 µL of product was taken following the capping of the RNA as well as from the final purified product and was run on an agarose gel containing 1% bleach to observe the quality of the RNA.

Immunohistochemistry (IHC) and automated detection

TMAs were prepared as previously described (20). In brief, paraffin moulds were cast using an Arraymold Kit (IHC World, Kit D, IW-115, core diameter 2 mm, 36 cores. Paraffin-embedded sections of human BRCA samples were processed into patient-matched transformed and non-transformed TMAs cut into 2 μm thick consecutive sections with a microtome (Leica). Before staining, slides were de-paraffinized and rehydrated using the following protocol: 3 × 5 min in xylene, 2 × 2 min in EtOH (100%), 2 × 2 min in EtOH (95%), 2 × 2 min in EtOH (70%), 2 min in EtOH (50%) and 5 min in H2O. After deparaffinisation and rehydration, antigen retrieval was performed with 10 mM sodium citrate buffer (pH 6.0) in a microwave oven at 800 W, 650 W and 360 W for 5 min, respectively. The samples were permeabilised with TBS 0.1 % Triton X-100 for 10 min and the endogenous Peroxidase were blocked with TBS containing 3 % H_2O_2 for 10 min. Finally, the samples were blocked for 1 h at room temperature with 10 % goat-serum, 1.5 % BSA in TBS. The human samples were stained with anti-PTEN (PTG) and anti-USP10 (HPA). Slides were developed with the SuperBoost™ HRP coupled secondary antibodies and with SignalStain® DAB Substrate Kit and counterstained with Hematoxylin (Sigma H3136). Slides were scanned in 40x resolution using a Olympus VS100 slide scanner and analysed using QuPath (version 0.5.0). (https://qupath.github.io/). Similar results were observed by trained pathologist (JG).

PTEN	Proteintech Europe / PTGlab	10047-1-AP	AB_2174343
USP10	Sigma/ HPA	hpa006731	AB_1080495
SignalStainR DAB Substrate Kit	Cell Signaling	8059 S	
SuperBoost™ Goat anti-Mouse Poly HRP	Thermo Fisher Scientific	B40961	
SuperBoost™ Goat anti-Rabbit Poly HRP	Thermo Fisher Scientific	B40962	

Soft agar assay for colony formation:

Bottom layer of agar (0.6ml) (0.6% agar in culture medium with10% FCS) was allowed

to solidify in a 6- well plate. Cell suspension (0.5ml) containing 4×10^4 cells in 0.36% agar in culture medium with 10% FCS (middle layer) was poured over the bottom layer. The middle layer was allowed to solidify for 30 minutes at room temperature before 0.5 ml of culture media (upper layer) was poured on top of middle layer. Plates were incubated for 2-3 weeks at 37°C with 5% CO_2 atmosphere. Plates were stained with 20 μL of MTT reagent (1mg/ml) for 4 hours. Colonies which contained at least 50 cells were counted. The colony numbers were counted using image J software.

Generation of PI3Ki resistant cells:

PI3K inhibitor resistance MCF7 and T47D cells were generated by maintaining the cells in media containing each of three different PI3K-inhibitors (BYL719, GDC068, and GDC0941). The resistant cells generated were named as R1, R2 and R3 based on the following description. Briefly, the concentration of drug used for generating R1 resistant cells was doubled after each three days starting from 50nM to 1000nM. R2 were maintained in constant drug concentration of 500nM. After 3 months, the drug concentration of R2 was changed to 1μM and these cells were named as R3.

PIP_3/PI(4,5)P2 Quantification

Cells were plated at a density of 1.3 × 10^7 cells per 10 cm dish. Following treatment, the medium was aspirated, and 5 ml of ice-cold 0.5 M TCA was promptly added. The cells were then scraped, transferred to a 15 ml tube on ice, and centrifuged at 3,000 rpm for 7 min at 4 C. The resulting pellet was resuspended in 3 ml of 5% tricarboxylic acid/1 mM EDTA, vortexed, and centrifuged at 3,000 rpm for 5 min. The supernatant was discarded, and this washing step was repeated once more. Subsequently, neutral lipids were extracted by adding 3 ml of MeOH:CHCl3 (2:1) and vortexing continuously for 10 min at room temperature. The extracts were centrifuged at 3,000 rpm for 5 min, the supernatant was discarded, and this extraction step was repeated. Acidic lipids were then extracted by adding 2.25 ml MeOH:CHCl3:12 M HCl (80:40:1) with continuous vortexing over 25 min at room temperature. The resulting extracts were centrifuged at 3,000 rpm for 5 min, and the supernatant was transferred to a new 15 ml tube. To the supernatant, 0.75 ml of CHCl3 and 1.35 ml of 0.1 M HCl were added, followed by vortexing and centrifugation at 3,000 rpm for 5 min to separate the organic and aqueous phases. The organic (lower) phase was collected; 1.45 ml were transferred into a new vial for PIP3 measurement, and 0.05 ml were transferred into another vial for PI(4,5)P2

measurement. All samples were dried in a vacuum dryer for 1 hr. PIP3 samples were resuspended in 120 ml of PBS-Tween+3% Protein Stabilizer (provided by the Echelon kit), while PI(4,5)P2 samples were re-suspended in 120 ml of PBS+0.25% Protein Stabilizer. The samples were sonicated in an ice-water bath for 10 min, vortexed, and spun down before being added to the ELISA. All experiments were conducted at least three times, each in biological triplicate. Once phospholipids were isolated from cells, PIP3 and PI(4,5)P2 levels were measured using ELISA kits (Echelon, K-2500s and K4500) according to the manufacturer's instructions. T47D PTEN null cells was a kind gift from Ioannis Sanidas.

PDX-organoids (PDXO) ex vivo cultures

PDX-derived cells were isolated from PDX through combination of mechanic disruption and enzymatic disaggregation (21). Briefly, PDX tumors not bigger that 500mm3 were freshly collected in DMEM/F12/HEPES (Gibco) after surgery resection, minced using sterile scalpels and dissociated for a 60 min in DMEM/F12/HEPES (Gibco), 1mg/ml collagenase (Roche), 100 u/ml, hyaluronidase (Sigma-Aldrich), 5% BSA (Sigma-Aldrich), 5 μg/ml Insulin and 50 μg/ml gentamycin (Gibco). This was followed by further dissociation using trypsin (Gibco), dispase 5 mg/ml (StemCell technologies) and DNase 1mg/ml (Sigma-Aldrich). Red blood cell lysis was done by washing the cell pellet with 1X Red Blood Cell (RBC) Lysis Buffer containing ammonium chloride (Invitrogen). Then, cells were resuspended in RPMI 1640 with GlutaMAX medium (Gibco) supplemented with 2% of heat inactivated fetal bovine serum (Gibco), 10 μM ROCK inhibitor (Sigma-Aldrich), 1X B-27 Supplement (Invitrogen), 3 μg/ml EGF (Peprotech), 5 μg/ml Insulin (Roche), 1 μg/ml Hydrocortisone (Sigma), Gentamycin (Gibco), 15 μg/ml Fungizone (Gibco)(22). To test drug antiproliferative responses and for immunoblotting analysis, cells were seeded on top of collagen-enriched matrix Corning® Matrigel® growth factor reduced (GFR) basement membrane matrix (Corning, INC) at $2x10^5$ cells/ml in 8 well-chamber slides (NUNC) or 10^6 cells/ml in 6 well plates (BD Biosciences), respectively. For antiproliferative analysis, PDXO were treated 24h after seeding for 7 days with vehicle (DMSO), 2 μM of BYL719, 5 μM of Spautin-1 or the combination and cultured at 37 °C in 5% of CO2. Duplicates were performed for each experimental condition. Medium and treatments were refreshed every 2–3 days. Matrigel® was melted in PBS-EDTA 1mM on ice for 20 minutes and plate readings were performed using Cell Titer Glo® Luminescent Cell Viability Assay (Promega). For immunoblotting analysis, cells were treated for 24 h. Matrigel® was melted in PBS-EDTA 1mM on ice for 20 min, the

spheroids were collected into a conical tube and centrifuged at 450 *g* for 5 min at 4 °C. Pellets were stored at −20 °C until protein lysates were prepared for immunoblotting analysis (see above).

TCGA data analysis

Correlation of USP10 with tumour grade was extracted from breast cancer microarray gene expression database (n=3,992) curated previously (23). Kaplan-Meier curves from TCGA data extrapolated from the Gepia2: Genomics Expression Profiling Interactive Analysis (http://gepia2.cancer-pku.cn)

RNA-Seq

MCF7 WT and MCF7 USP10 KO cells were cultured as above in 10 cm dishes, RNA was extracted from five concurrent passages of cells using Bioline Isolate II RNA extraction kit (BIO-52072) as per manufacturer instructions. RNA quality and quantity was confirmed using ThermoFisher Nanodrop 1000. 3 µg of RNA, per sample, was prepared in RNA stabilised tubes and sent to Azenta for RNA sequencing. RNA-seq data generated in this study have been deposited in the NCBI Gene Expression Omnibus (GEO) under accession number GSE302500.

RNASeq analysis

Prior to alignment, adaptor sequences were trimmed via trim galore (v0.6.7) using default parameters for paired-end reads. All samples were then aligned to the hg38 build of the human genome using Rsubread (v2.10.5) (24). In all cases, at least 97% of fragments were successfully mapped to the genome. Fragments overlapping genes were then summarized using Rsubread's featureCounts function. Genes were identified using RefSeq annotation to the hg38 genome. Differential expression analyses were then carried out using the limma (v3.52.4) (25)and edgeR (v3.38.4) (26) software packages.

Prior to analysis expression based filtering was performed using edgeR's filterByExpr function with default parameters. A total of 14,670 genes remained for downstream analysis. Sample composition was then normalized using the TMM method (27).

Following filtering and normalization, the data was transformed to $\log_2$-counts per million (CPM), and the correlation between samples from the same collection passage estimated using limma's duplicate Correlation function. Differential expression between the UPS10 knock-out (KO) and wild type (WT) groups was then assessed relative to a fold-change threshold of 1.5 using linear models, incorporating the correlation estimate, and robust

empirical bayes moderated t-statistics with a trended prior variance (treat limma-trend pipeline) (28, 29)The Benjamini and Hochberg method was applied to control the false discovery rate (FDR) below 5%. Pathway analyses of the Hallmark gene sets supplied by the Molecular Signatures Databases were carried out using GSEA (v4.3.2) (30)

Spatial Transcriptomic Data

The analysis of public available data was conducted as previously described (31). In brief, analysis of public available Spatial Transcriptomic data was done as outlined by the manufacturer (10XGenomics; https://www.10xgenomics.com/datasets?query=&page=1&configure%5BhitsPerPage%5D=50&configure%5BmaxValuesPerFacet%5D=1000). To this end we used the GUI-based data analysis tool Loupe (v8) to retrieve public available datasets and to visualize and extract transcriptomic data from Visium and Visium HD slides. K means were used to discriminate between clusters of cells expressing similar genes and sharing comparable transcriptomic features. The decision for the extent of clustering was taken by consulting the H&E section of the tissue analysed and the clustering of tissue parts with similar features (e.g. all murine crypts in one cluster). Transcriptomic data was extracted as .csv and analysed using GraphPad.

Expression and purification of recombinant UbV10

UbV10 was cloned into a pOPINB vector (Addgene) via In-Fusion cloning (Takara Bio). The UbV10 insert sequence was designed from Zhang et al.(32). This vector was transformed into *Escherichia coli* BL21 (DE3) cells (Novagen) and cultured at 37 °C in 2xYT medium until optical density at 600 nm reached 0.8. Cultures were cooled to 18 °C before inducing with 0.3 mM isopropyl ß-D-1-thiogalactopyranoside (IPTG) and incubating overnight. Cultures were centrifuged at 5,000 *g* for 10 min and pellets were resuspended in lysis buffer (50 mM HEPES pH 8.0, 300 mM NaCl) supplemented with Protease inhibitor cocktail (Complete EDTA-free tablet, Roche). Resuspended pellets were lysed with a sonicator and centrifuged at 40,000 *g* for 30 min at 4 °C.

To purify UbV10, lysate was applied to HisPur nickel resin (Thermo Sc.) and then washed with lysis buffer with 20 mM imidazole before eluting with lysis buffer with 300 mM imidazole. His-tag was cleaved with His-3C PreScission Protease during overnight dialysis back into lysis buffer. His-3C was captured by applying dialysed protein back over nickel resin and collecting the flowthrough. UbV10 was finally purified by size-exclusion chromatography using a

HiLoad 16/600 Superdex 75 pg column (Cytiva) in 20 mM HEPES pH 8.0, 200 mM NaCl, 1 mM TCEP. Pure fractions were pooled, concentrated and flash-frozen in liquid nitrogen.

UbVS probe synthesis

Ubiquitin 1-75 MesNa was generated according to Wilkinson et al.(33) UbVS probe was generated according to Gladkova et al. (34). The probe was purified by cation exchange chromatography by diluting 1:50 in 50 mM sodium acetate pH 4.5 and loading on a Resource S 6 mL column (Cytiva). The probe was eluted with a linear gradient of 55 column volumes 0-35% using 50 mM sodium acetate without and with 1 M NaCl respectively. Correct UbVS mass was verified by intact mass spectrometry to differentiate unreacted ubiquitin MesNa from UbVS. Correct fractions were pooled and dialysed in storage buffer (20 mM HEPES pH 6.5, 50 mM sodium acetate, 75 mM NaCl) before concentrating and flash-freezing in liquid nitrogen.

Single-cell RNA sequencing data acquisition and analysis

Karaayvas et al.'s single-cell RNA sequencing data of triple-negative breast cancer patients, along with cell type annotations, were acquired from weizmann.ac.il (35, 36). Data preprocessing involved the exclusion of cells exhibiting a high percentage of mitochondrial genes (> 20%), a high percentage of ribosomal genes (>25%), and a low number of detected genes (< 50 genes). Subsequently, the data were normalized, log-transformed, and scaled. The top 2000 variable features were selected for principal component analysis (PCA). To address patient-derived batch effects, batch correction was performed using harmony. Cell type annotations were obtained from weizmann.ac.il, where they relied on canonical markers as well as inferred Copy Number Aberrations (CNAs). The expression of USP10 across all cell types was visualized using a violin plot, and statistical significance was determined using the Wilcoxon test. Data processing and batch correction were executed utilizing Seurat (Version 5.0.0) (37). Visualization was facilitated by the scCustomize and SCpubr R packages. The analysis was conducted using R version 4.3.2.

References

1. Yang J, and Zhang Y. I-TASSER server: new development for protein structure and function predictions. *Nucleic Acids Res.* 2015;43(W1):W174-W81.
2. Liu L, Kohout SC, Xu Q, Muller S, Kimberlin CR, Isacoff EY, et al. A glutamate switch controls voltage-sensitive phosphatase function. *Nat Struct Mol Biol.* 2012;19(6):633-41.
3. Heinrich F, Chakravarthy S, Nanda H, Papa A, Pandolfi Pier P, Ross Alonzo H, et al. The PTEN Tumor Suppressor Forms Homodimers in Solution. *Structure.* 2015;23(10):1952-7.
4. Romelia S-F, A. CD, and C. WR. An overview of the Amber biomolecular simulation package. *Wiley Interdisciplinary Reviews: Computational Molecular Science.* 2013;3(2):198-210.
5. Maier JA, Martinez C, Kasavajhala K, Wickstrom L, Hauser KE, and Simmerling C. ff14SB: Improving the Accuracy of Protein Side Chain and Backbone Parameters from ff99SB. *Journal of Chemical Theory and Computation.* 2015;11(8):3696-713.
6. Homeyer N, Horn AHC, Lanig H, and Sticht H. AMBER force-field parameters for phosphorylated amino acids in different protonation states: phosphoserine, phosphothreonine, phosphotyrosine, and phosphohistidine. *Journal of Molecular Modeling.* 2006;12(3):281-9.
7. Steinbrecher T, Latzer J, and Case DA. Revised AMBER parameters for bioorganic phosphates. *Journal of chemical theory and computation.* 2012;8(11):4405-12.
8. Sousa da Silva AW, and Vranken WF. ACPYPE - AnteChamber PYthon Parser interfacE. *BMC Research Notes.* 2012;5(1):367.
9. Abraham MJ, Murtola T, Schulz R, Páll S, Smith JC, Hess B, et al. GROMACS: High performance molecular simulations through multi-level parallelism from laptops to supercomputers. *SoftwareX.* 2015;1-2:19-25.
10. Jorgensen WL, Chandrasekhar J, and Madura JD. Comparison of simple potential functions for simulating liquid water. *The Journal of Chemical Physics.* 1983;79(2):926-35.
11. Bussi G, Donadio D, and Parrinello M. Canonical sampling through velocity rescaling. *The Journal of Chemical Physics.* 2007;126(1):014101.
12. Berendsen HJC, Postma JPM, Gunsteren WFv, DiNola A, and Haak JR. Molecular dynamics with coupling to an external bath. *The Journal of Chemical Physics.* 1984;81(8):3684-90.
13. Essmann U, Perera L, Berkowitz ML, Darden T, Lee H, and Pedersen LG. A smooth particle mesh Ewald method. *The Journal of Chemical Physics.* 1995;103(19):8577-93.
14. Hess B. P-LINCS: A Parallel Linear Constraint Solver for Molecular Simulation. *Journal of Chemical Theory and Computation.* 2008;4(1):116-22.
15. Miller BR, McGee TD, Swails JM, Homeyer N, Gohlke H, and Roitberg AE. MMPBSA.py: An Efficient Program for End-State Free Energy Calculations. *Journal of Chemical Theory and Computation.* 2012;8(9):3314-21.

16. Onufriev A, Bashford D, and Case DA. Exploring protein native states and large-scale conformational changes with a modified generalized born model. *Proteins: Structure, Function, and Bioinformatics.* 2004;55(2):383-94.
17. Weiser J, Shenkin PS, and Still WC. Approximate atomic surfaces from linear combinations of pairwise overlaps (LCPO). *Journal of Computational Chemistry.* 1999;20(2):217-30.
18. Brummelkamp TR, Nijman SM, Dirac AM, and Bernards R. Loss of the cylindromatosis tumour suppressor inhibits apoptosis by activating NF-kappaB. *Nature.* 2003;424(6950):797-801.
19. Palafox M, Monserrat L, Bellet M, Villacampa G, Gonzalez-Perez A, Oliveira M, et al. High p16 expression and heterozygous RB1 loss are biomarkers for CDK4/6 inhibitor resistance in ER(+) breast cancer. *Nat Commun.* 2022;13(1):5258.
20. Novak R, Abu Ahmad Y, Timaner M, Bitman-Lotan E, Oknin-Vaisman A, Horwitz R, et al. RNF4 similar to RGMb similar to BMP6 axis required for osteogenic differentiation and cancer cell survival. *Cell Death & Disease.* 2022;13(9).
21. Bruna A, Rueda OM, Greenwood W, Batra AS, Callari M, Batra RN, et al. A Biobank of Breast Cancer Explants with Preserved Intra-tumor Heterogeneity to Screen Anticancer Compounds. *Cell.* 2016;167(1):260-74 e22.
22. Driehuis E, Kretzschmar K, and Clevers H. Establishment of patient-derived cancer organoids for drug-screening applications. *Nat Protoc.* 2020;15(10):3380-409.
23. Tan TZ, Miow QH, Miki Y, Noda T, Mori S, Huang RY, et al. Epithelial-mesenchymal transition spectrum quantification and its efficacy in deciphering survival and drug responses of cancer patients. *EMBO Mol Med.* 2014;6(10):1279-93.
24. Liao Y, Smyth GK, and Shi W. The R package Rsubread is easier, faster, cheaper and better for alignment and quantification of RNA sequencing reads. *Nucleic Acids Res.* 2019;47(8):e47.
25. Ritchie ME, Phipson B, Wu D, Hu Y, Law CW, Shi W, et al. limma powers differential expression analyses for RNA-sequencing and microarray studies. *Nucleic Acids Res.* 2015;43(7):e47.
26. Robinson MD, McCarthy DJ, and Smyth GK. edgeR: a Bioconductor package for differential expression analysis of digital gene expression data. *Bioinformatics.* 2010;26(1):139-40.
27. Robinson MD, and Oshlack A. A scaling normalization method for differential expression analysis of RNA-seq data. *Genome Biol.* 2010;11(3):R25.
28. Phipson B, Lee S, Majewski IJ, Alexander WS, and Smyth GK. Robust Hyperparameter Estimation Protects against Hypervariable Genes and Improves Power to Detect Differential Expression. *Ann Appl Stat.* 2016;10(2):946-63.
29. McCarthy DJ, and Smyth GK. Testing significance relative to a fold-change threshold is a TREAT. *Bioinformatics.* 2009;25(6):765-71.
30. Subramanian A, Tamayo P, Mootha VK, Mukherjee S, Ebert BL, Gillette MA, et al. Gene set enrichment analysis: a knowledge-based approach for interpreting genome-wide expression profiles. *Proc Natl Acad Sci U S A.* 2005;102(43):15545-50.
31. Reissland M, Hartmann O, Tauch S, Bugter JM, Prieto-Garcia C, Schulte C, et al. USP10 drives cancer stemness and enables super-competitor signalling in colorectal cancer. *Oncogene.* 2024.
32. Zhang W, Sartori MA, Makhnevych T, Federowicz KE, Dong X, Liu L, et al. Generation and Validation of Intracellular Ubiquitin Variant Inhibitors for USP7 and USP10. *J Mol Biol.* 2017.

33. Wilkinson KD, Gan-Erdene T, and Kolli N. Derivitization of the C-terminus of ubiquitin and ubiquitin-like proteins using intein chemistry: methods and uses. *Methods Enzymol.* 2005;399:37-51.
34. Gladkova C, Maslen SL, Skehel JM, and Komander D. Mechanism of parkin activation by PINK1. *Nature.* 2018;559(7714):410-4.
35. Karaayvaz M, Cristea S, Gillespie SM, Patel AP, Mylvaganam R, Luo CC, et al. Unravelling subclonal heterogeneity and aggressive disease states in TNBC through single-cell RNA-seq. *Nat Commun.* 2018;9(1):3588.
36. Gavish A, Tyler M, Greenwald AC, Hoefflin R, Simkin D, Tschernichovsky R, et al. Hallmarks of transcriptional intratumour heterogeneity across a thousand tumours. *Nature.* 2023;618(7965):598-606.
37. Hao Y, Stuart T, Kowalski MH, Choudhary S, Hoffman P, Hartman A, et al. Dictionary learning for integrative, multimodal and scalable single-cell analysis. *Nat Biotechnol.* 2024;42(2):293-304.
