# Supplementary material for: USP10/GSK3β-mediated inhibition of PTEN drives resistance to PI3K inhibitors in breast cancer
Source: J Clin Invest. 2025 Sep 23;135(22):e180927. doi: 10.1172/JCI180927 (PMC12618074; doi:10.1172/JCI180927)

Full unedited gel for Figure 1B

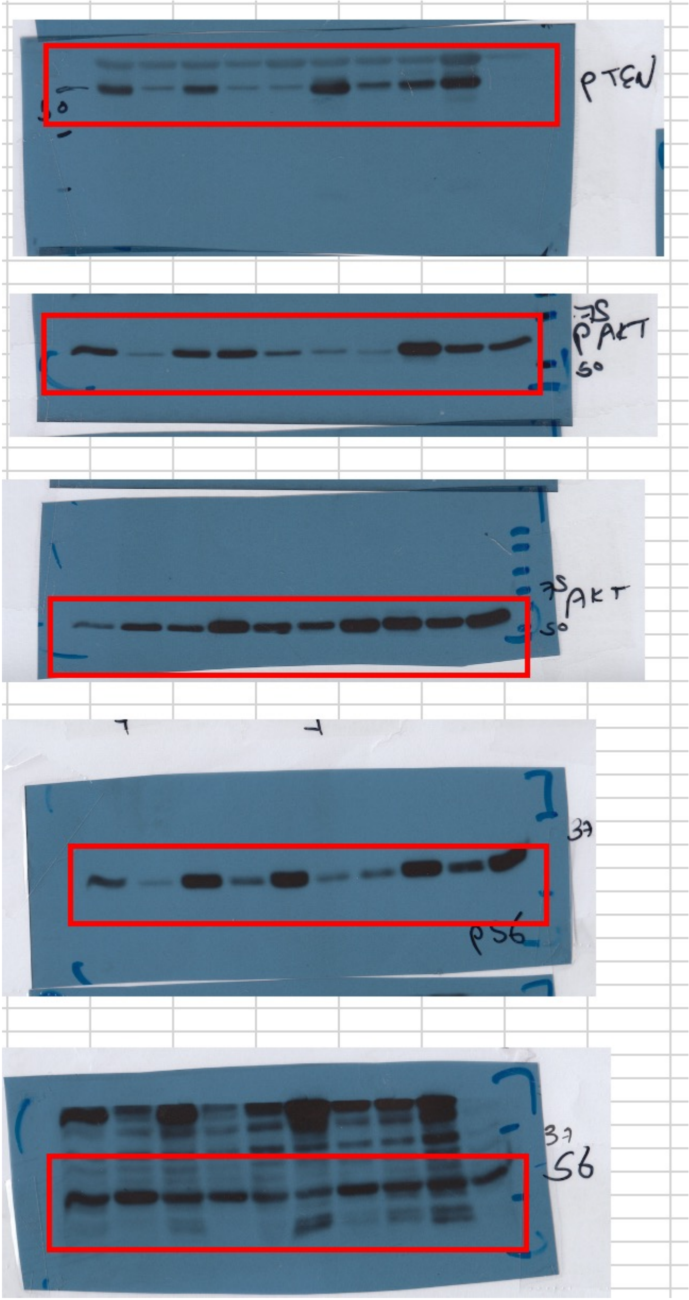

Full unedited gel for Figure 2A

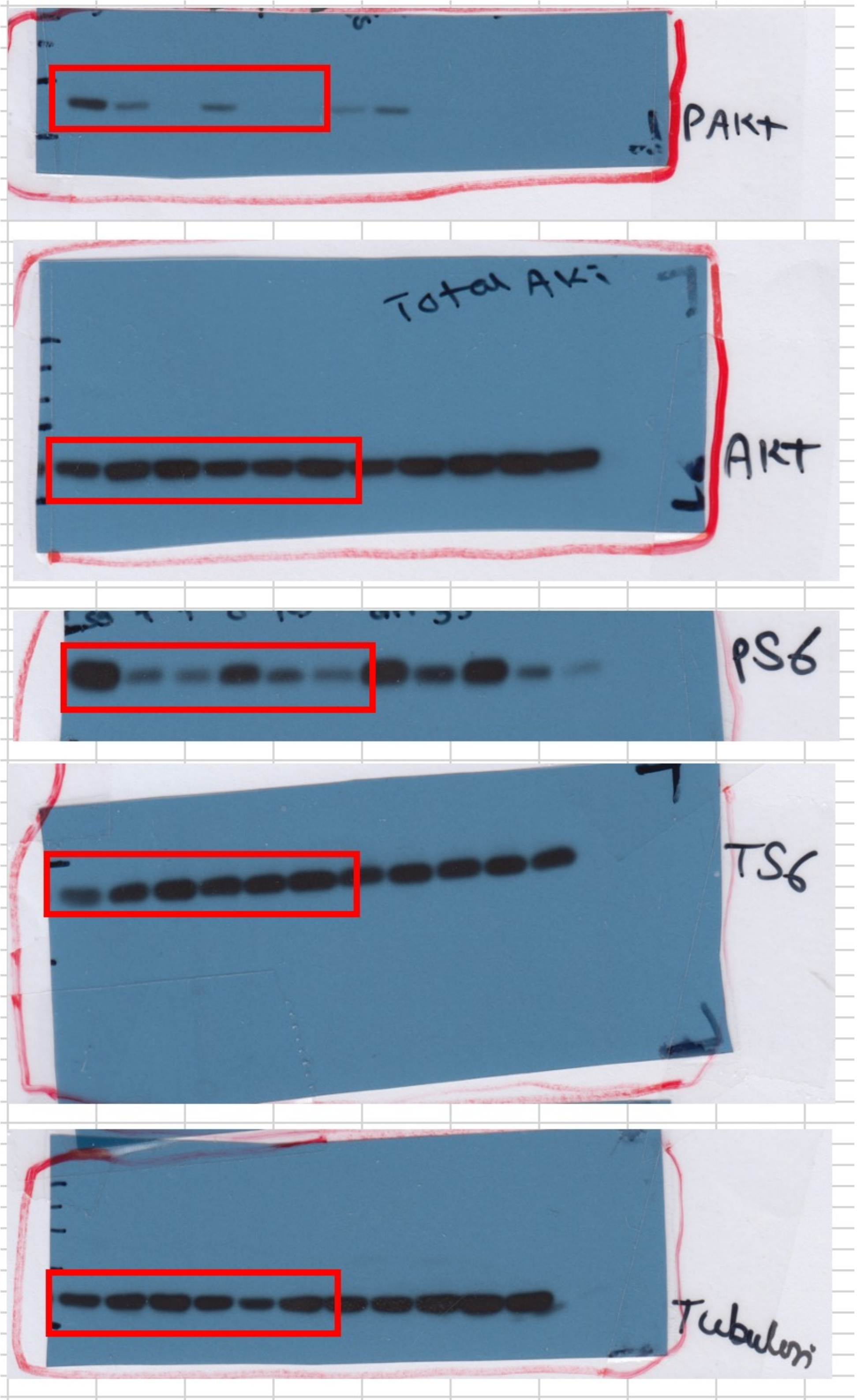

Full unedited gel for Figure 2B

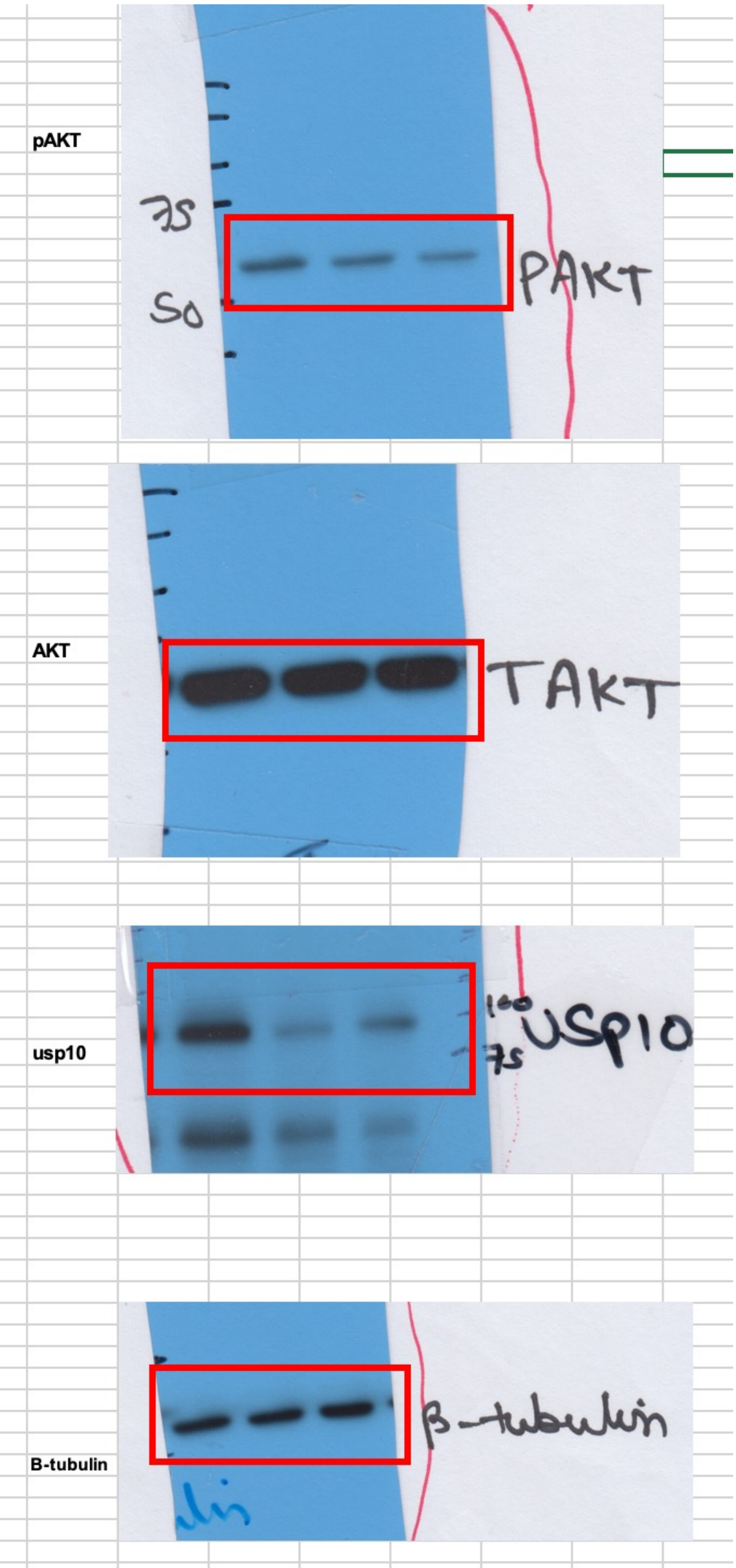

Full unedited gel for Figure 2C

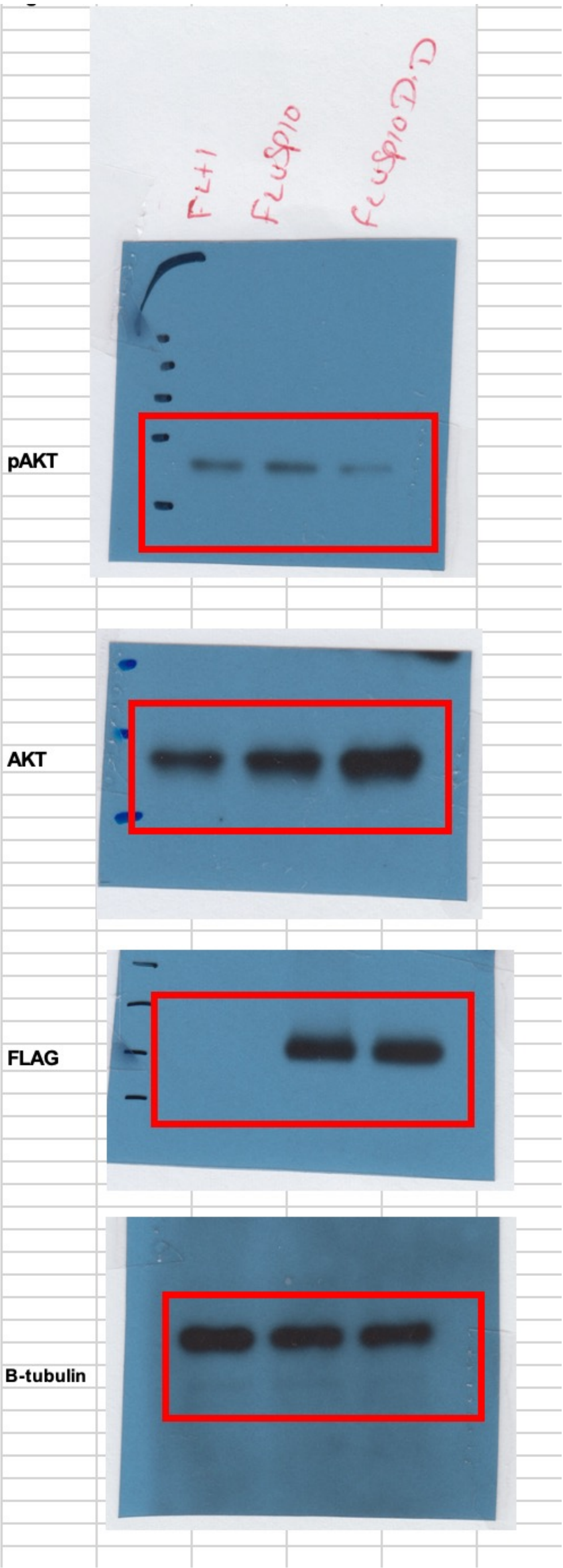

Full unedited gel for Figure 2D

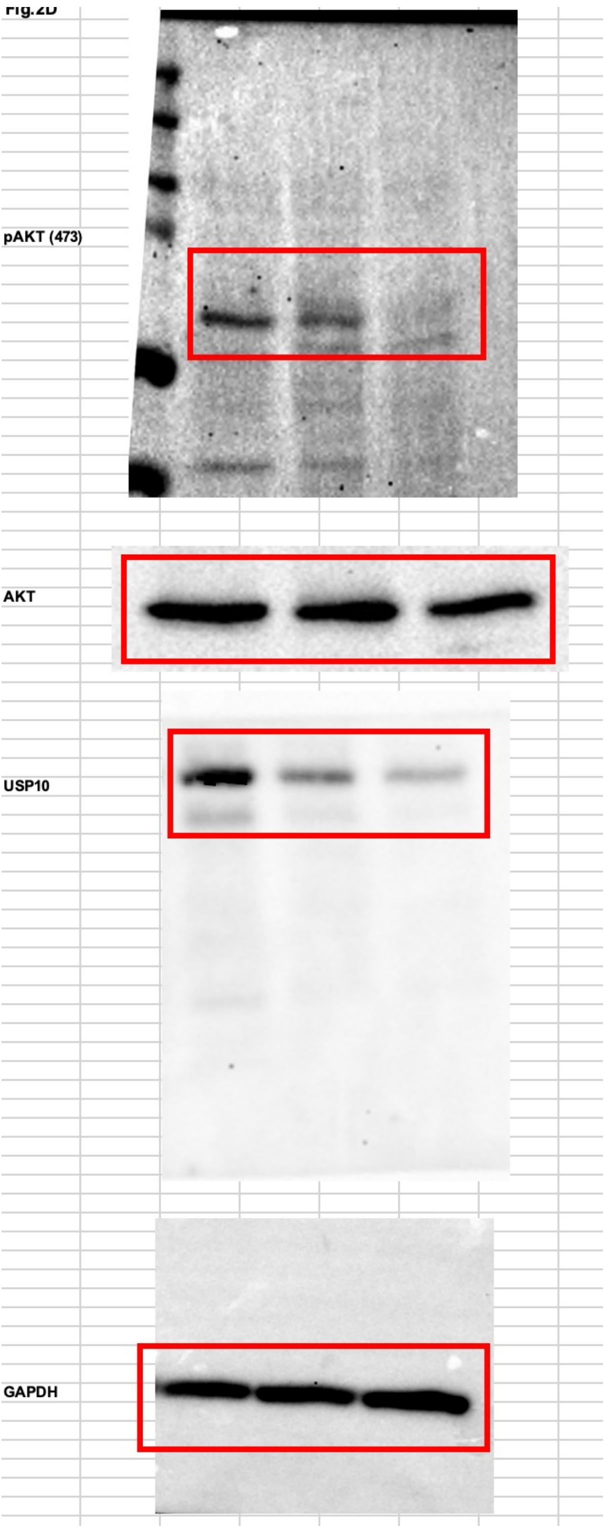

Full unedited gel for Figure 2E

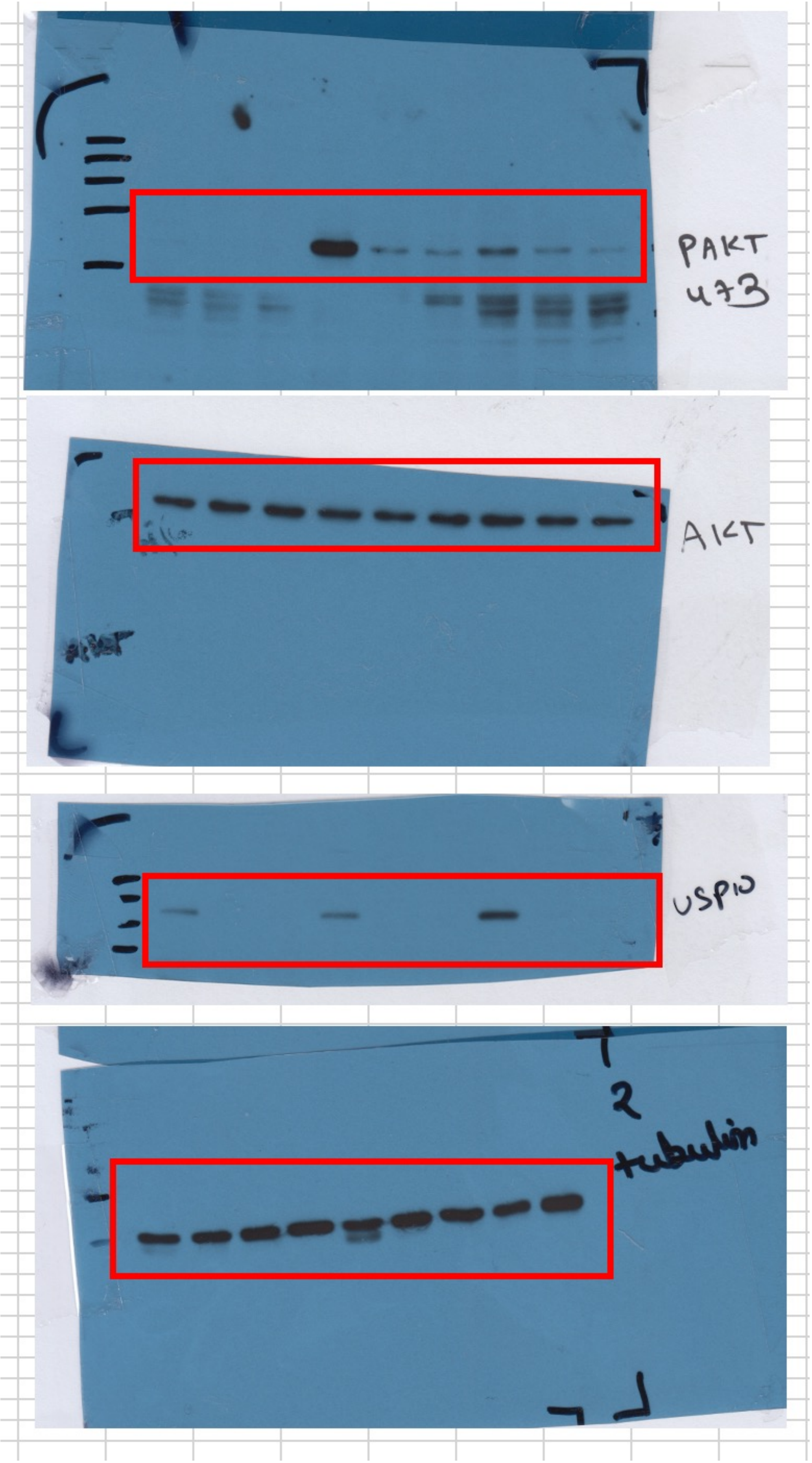

Full unedited gel for Figure 2F

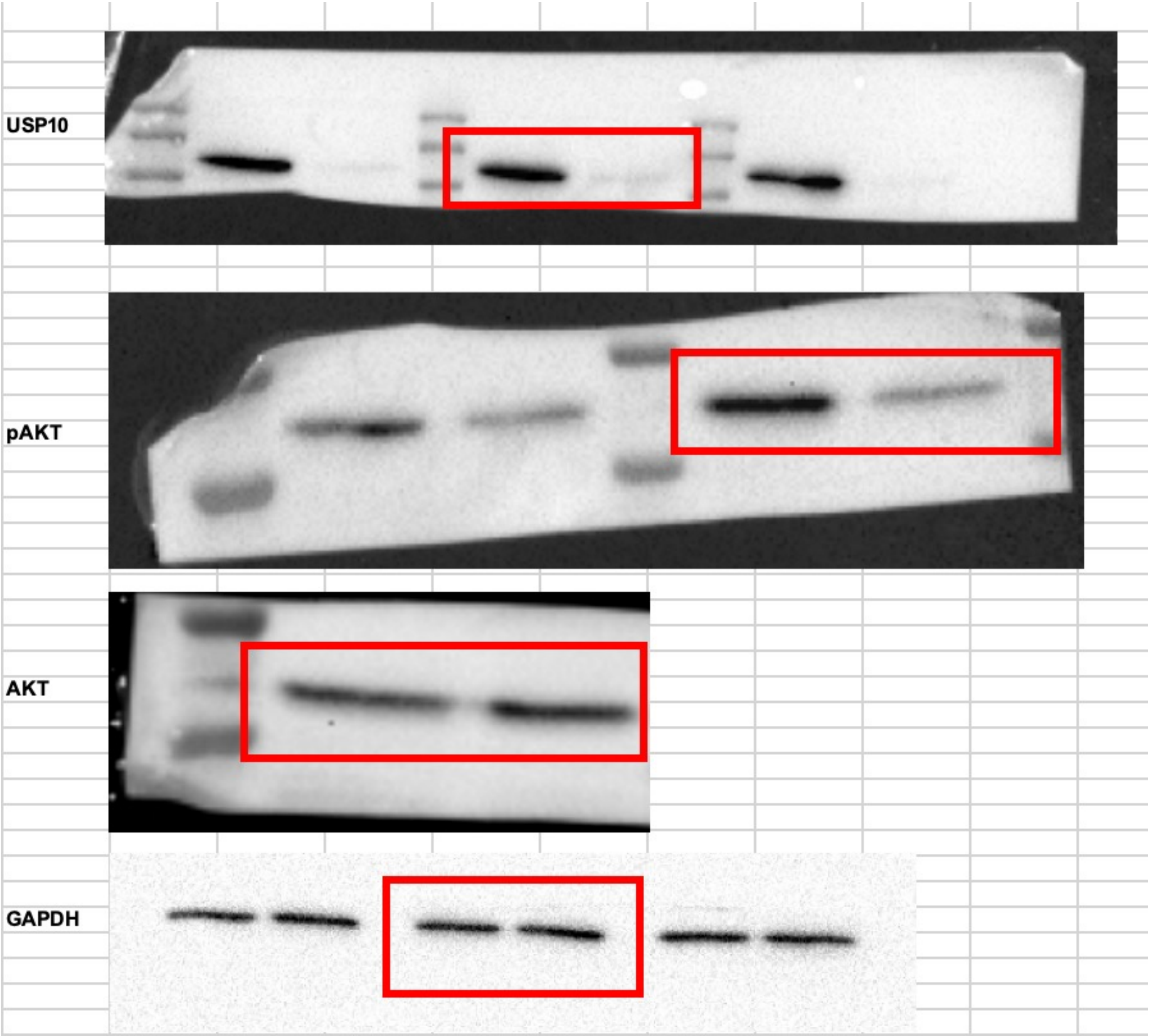

Full unedited gel for Figure 2G

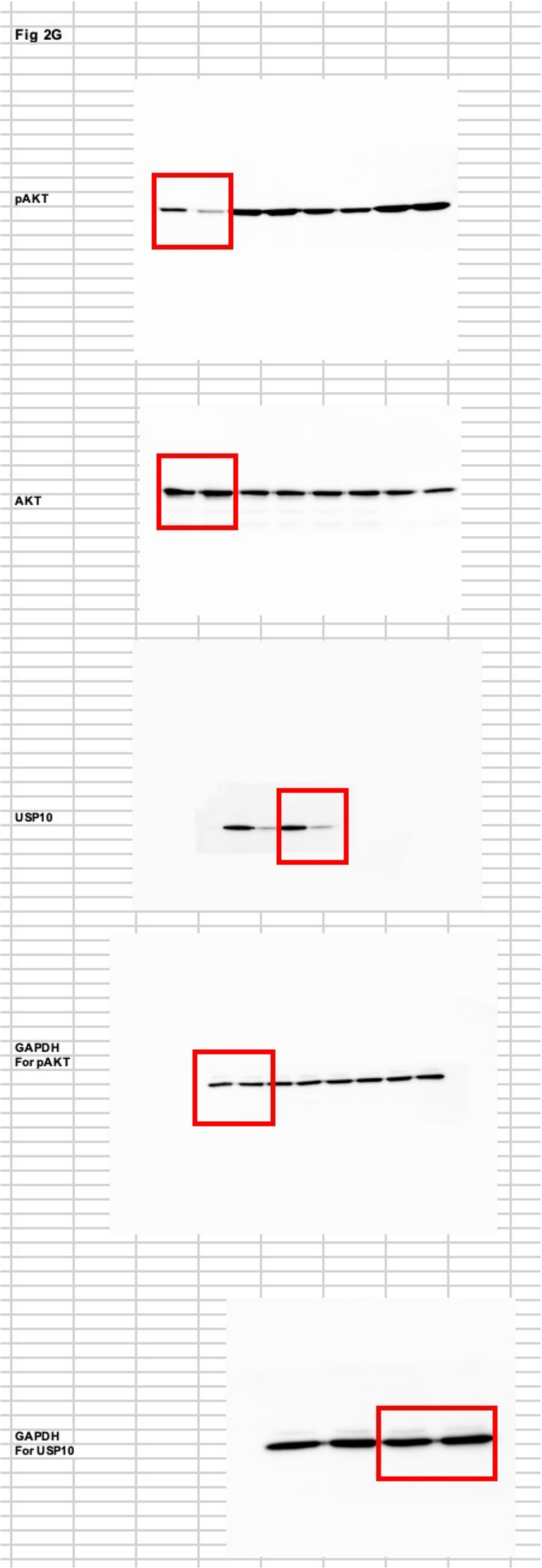

Full unedited gel for Figure 2M

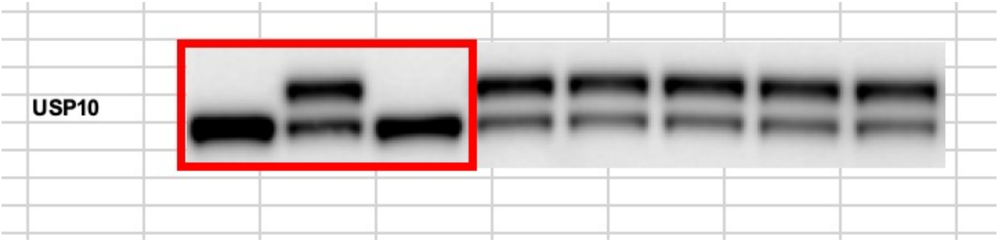

Full unedited gel for Figure [illegible]

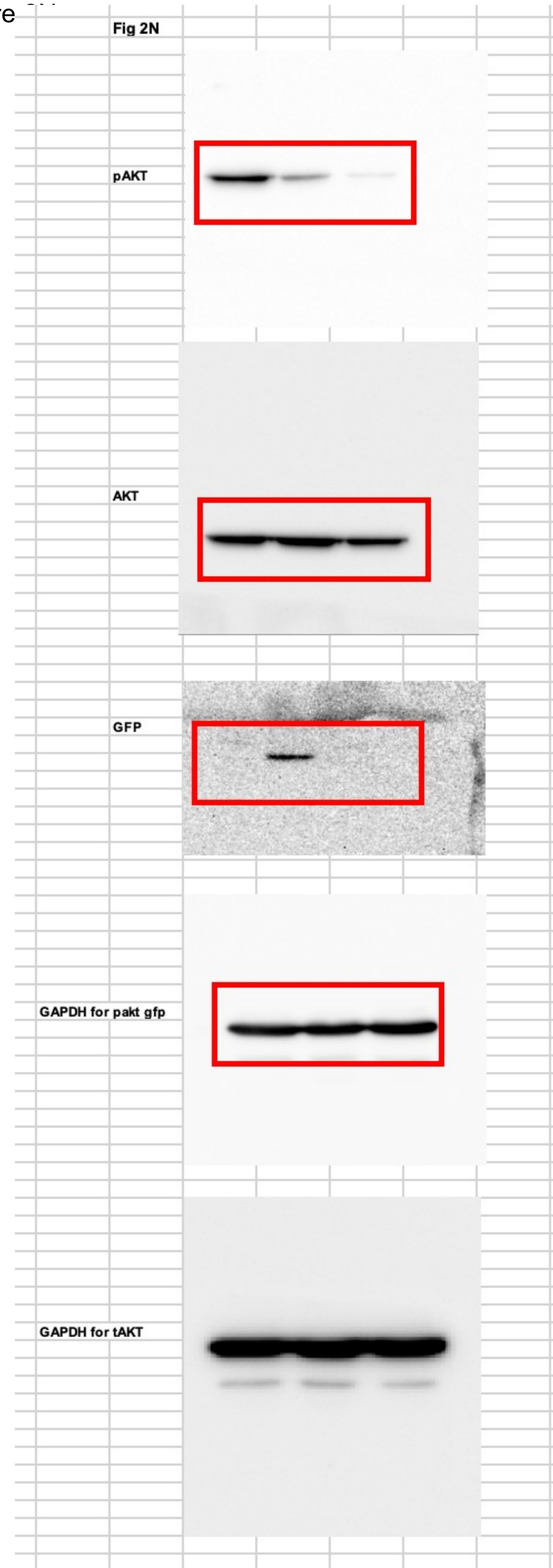

Full unedited gel for Figure 3A

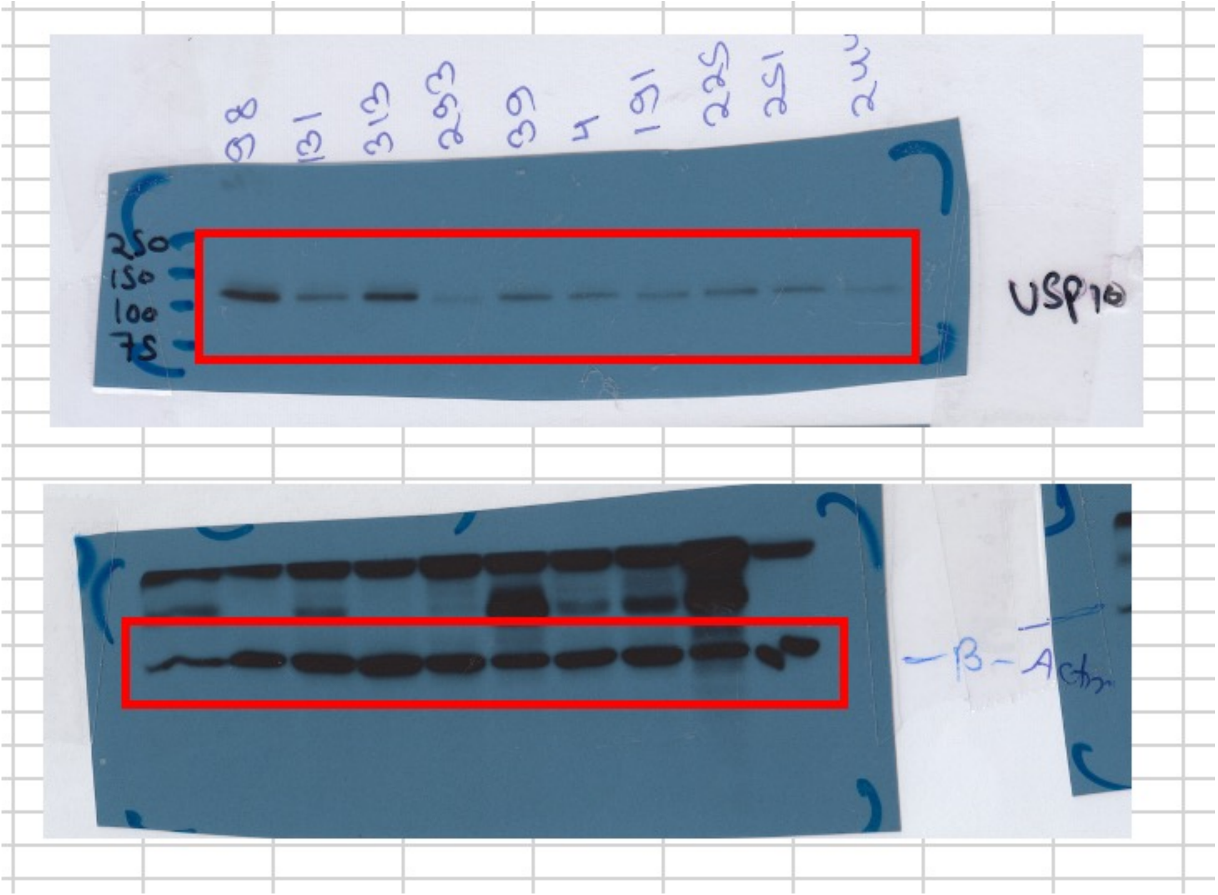

Full unedited gel for Figure 3C

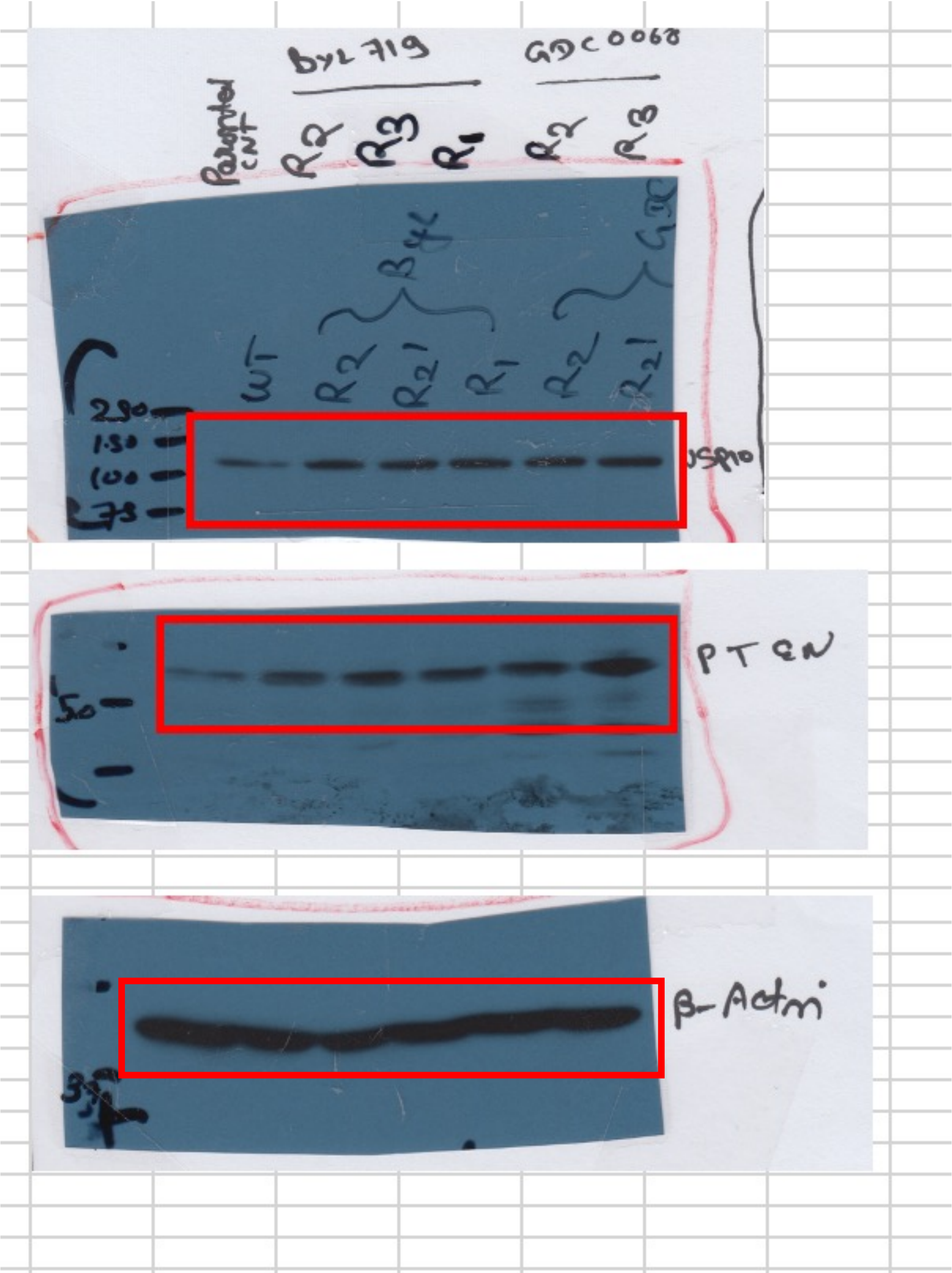

Full unedited gel for Figure 3D

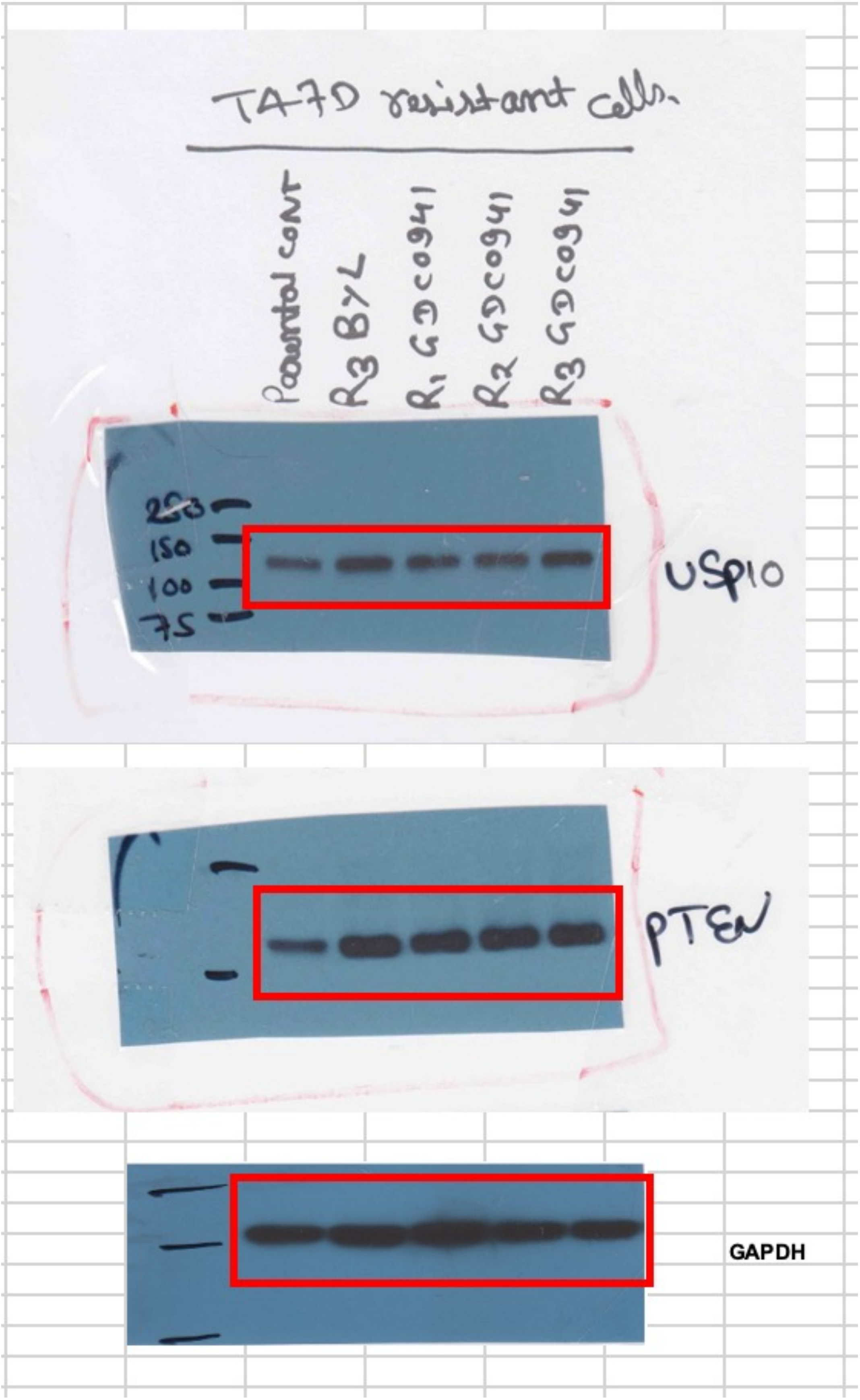

Full unedited gel for Figure 3E

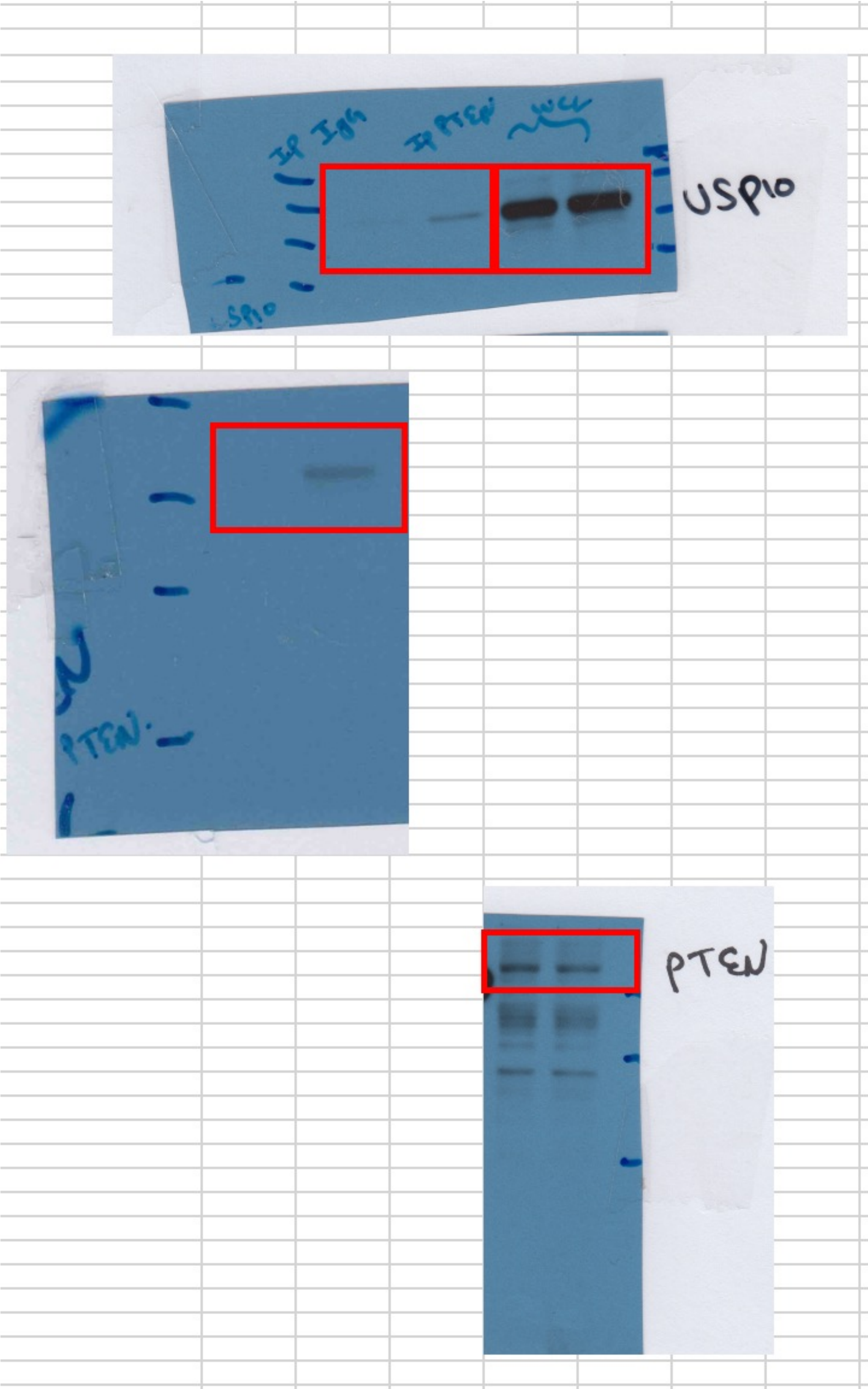

Full unedited gel for Figure 3F

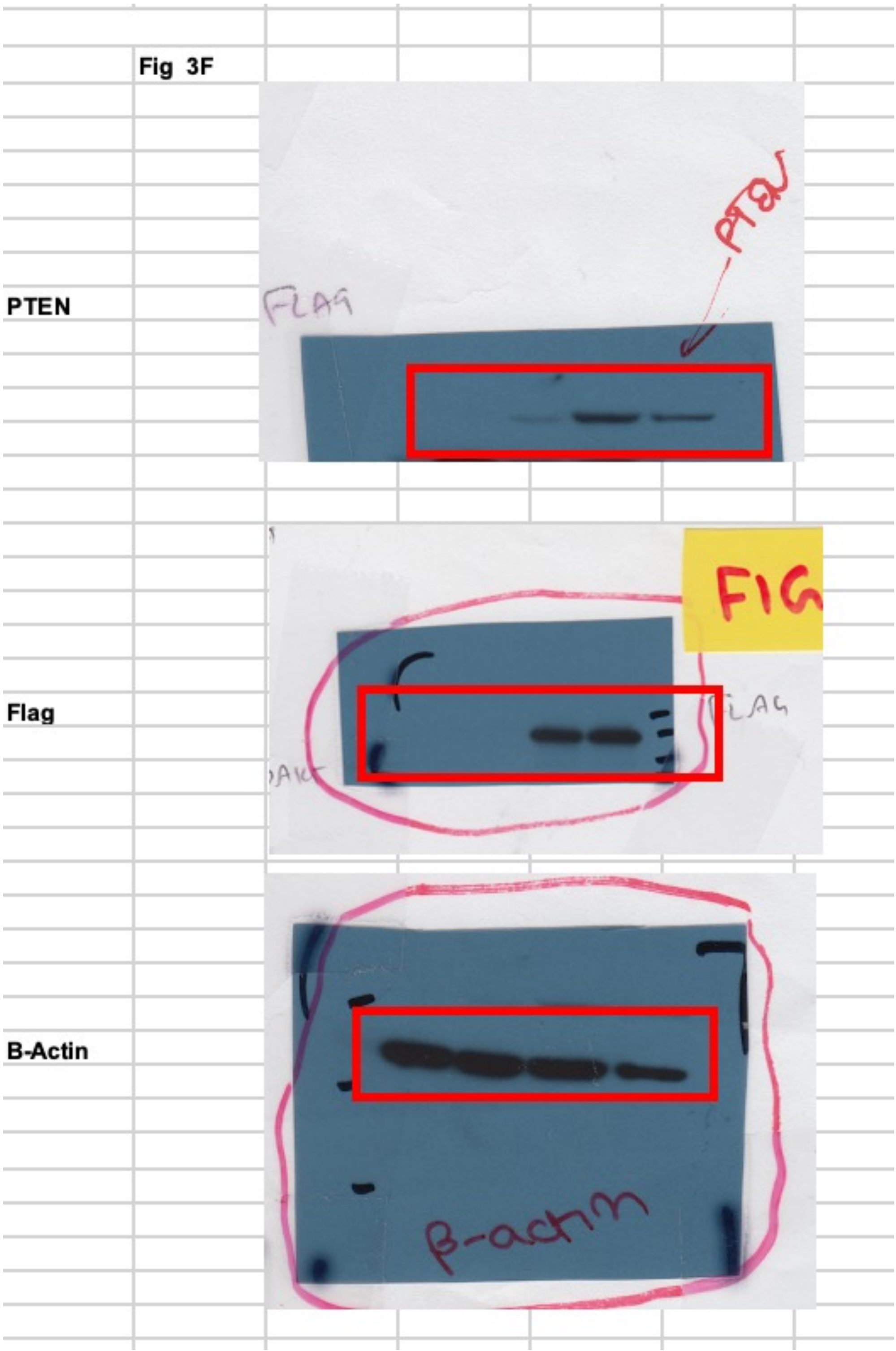

Full unedited gel for Figure 3G

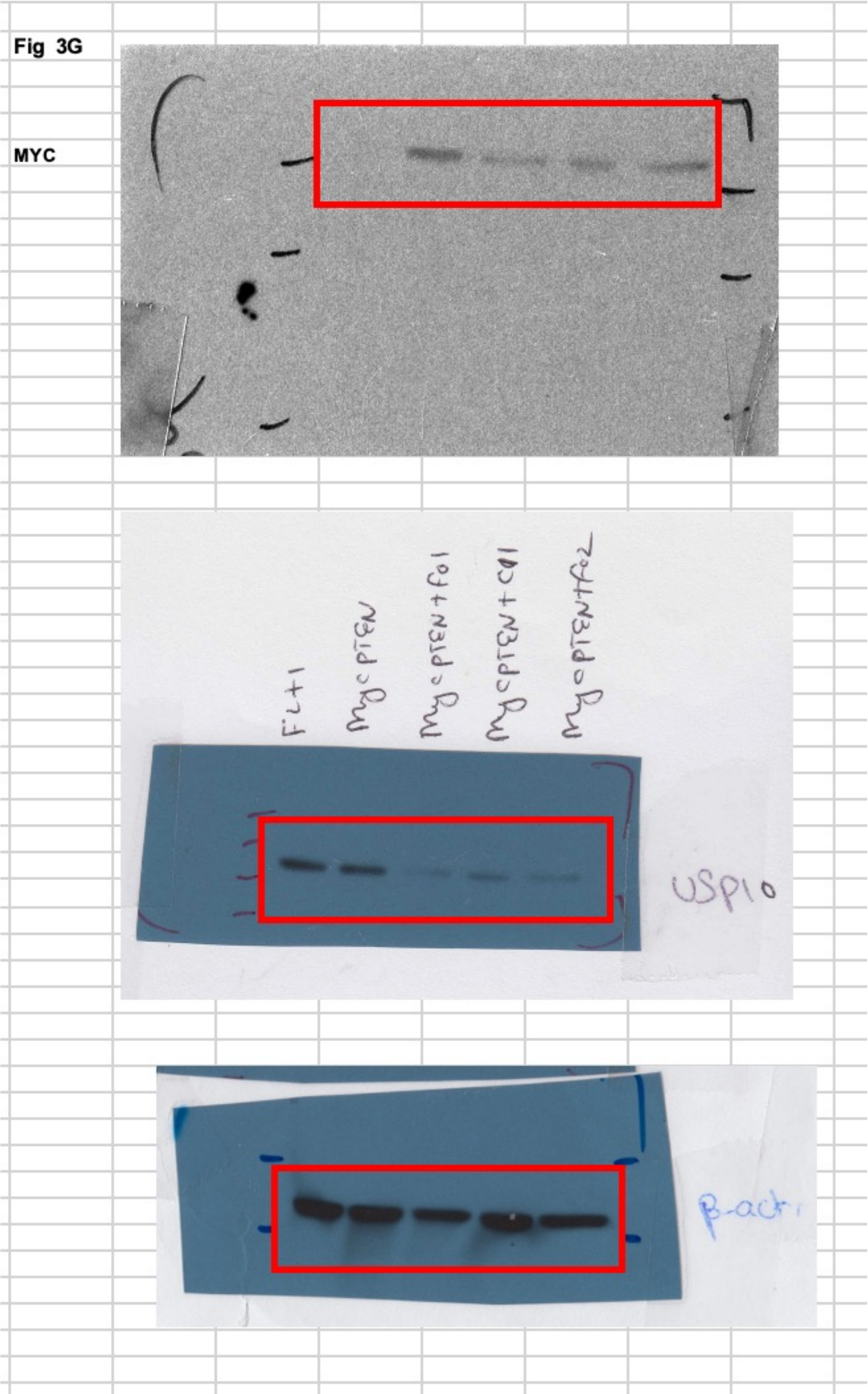

Full unedited gel for Figure 3H

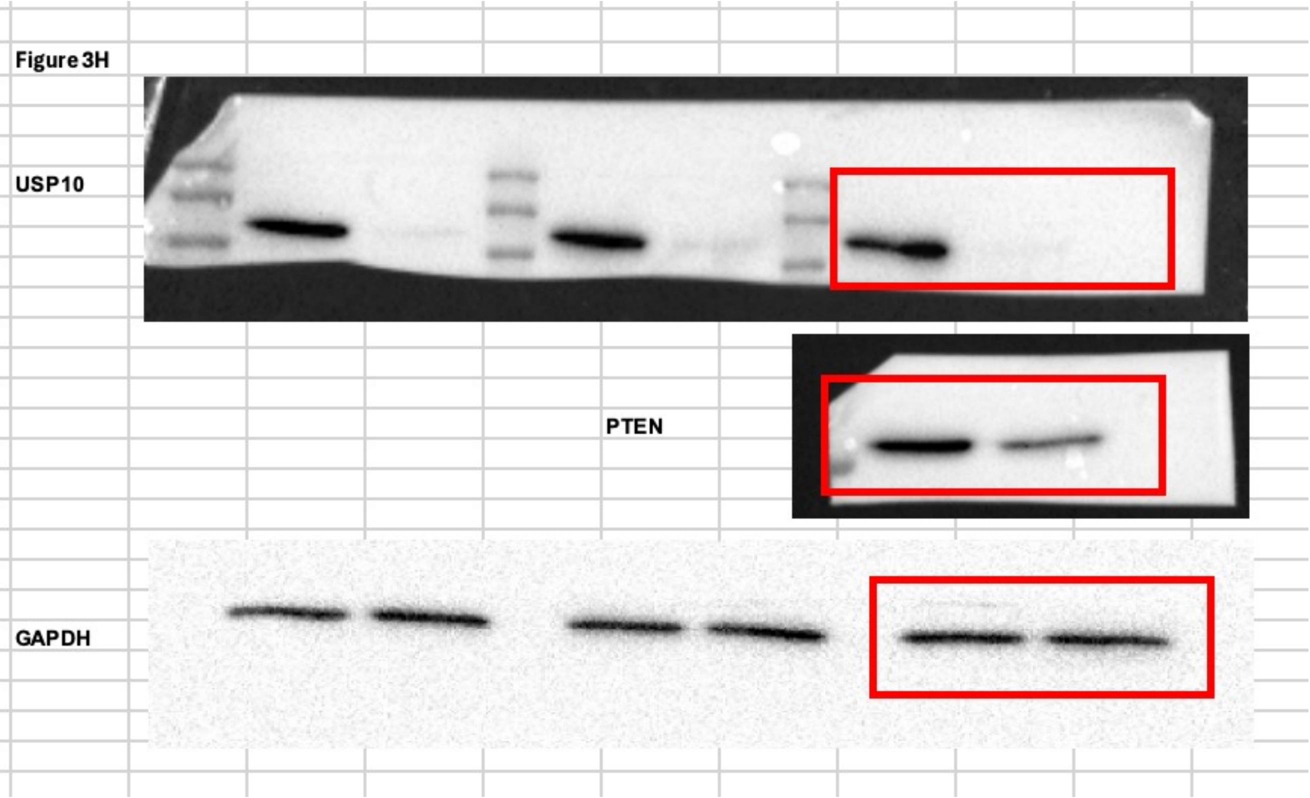

Full unedited gel for Figure 3L

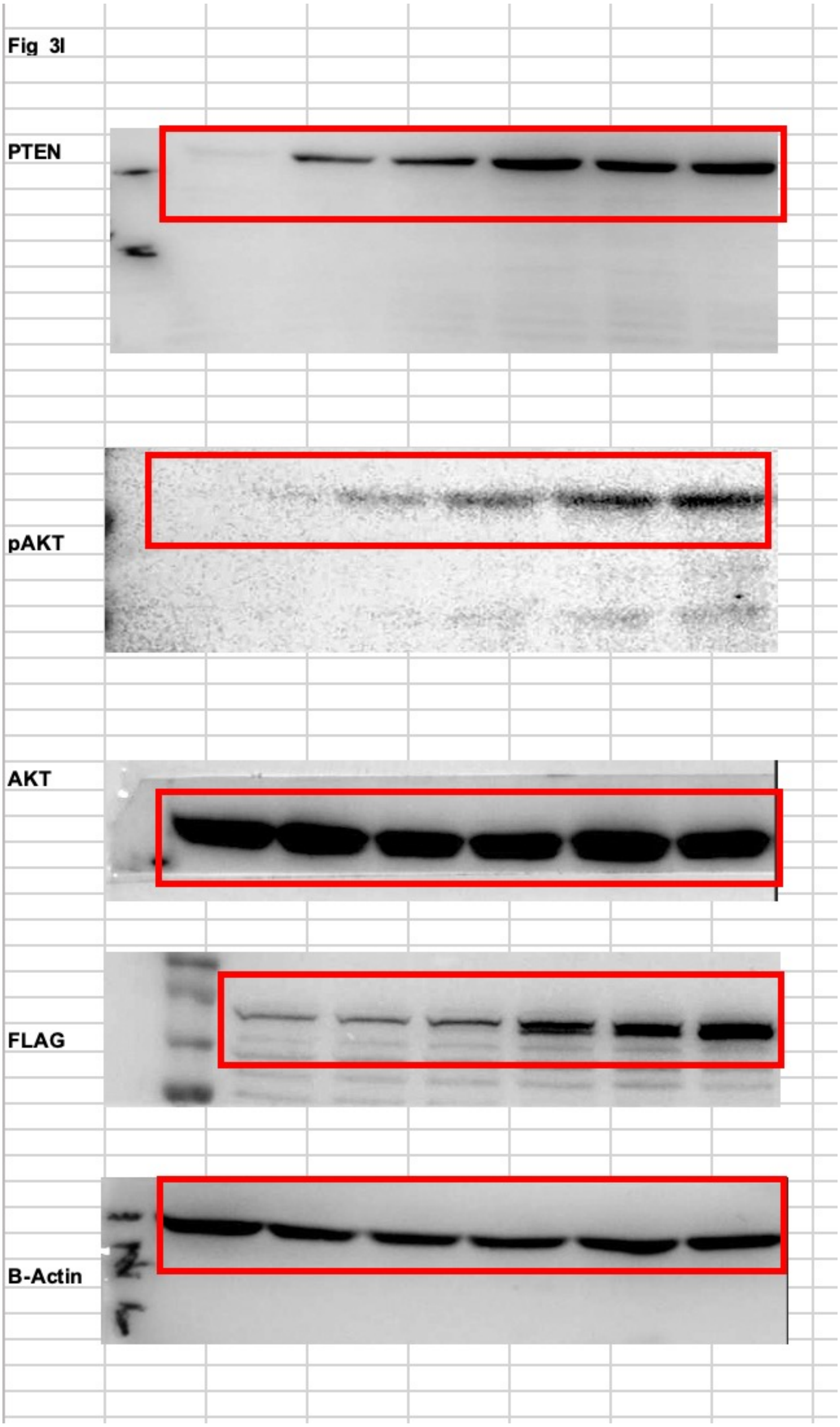

Full unedited gel for Figure 5B

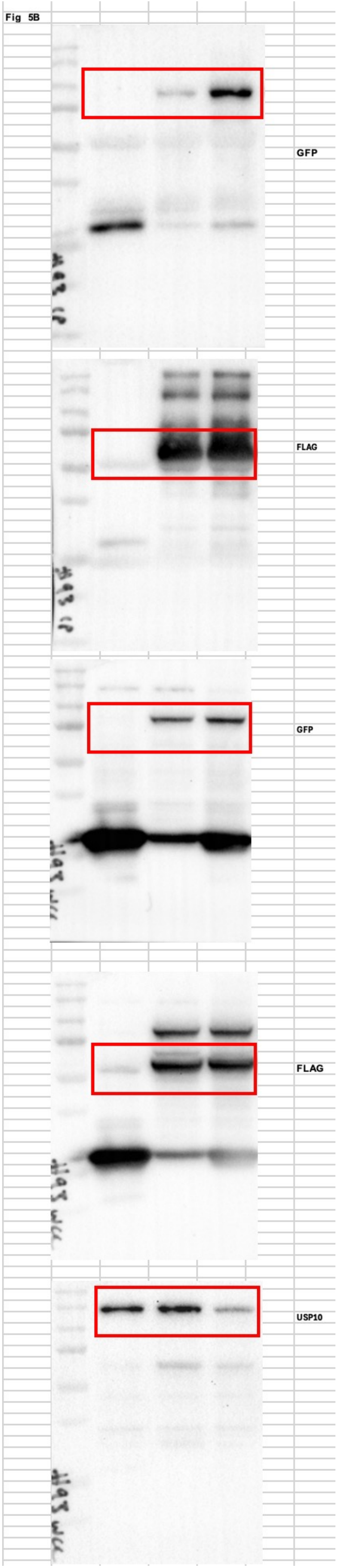

Full unedited gel for Figure 5C

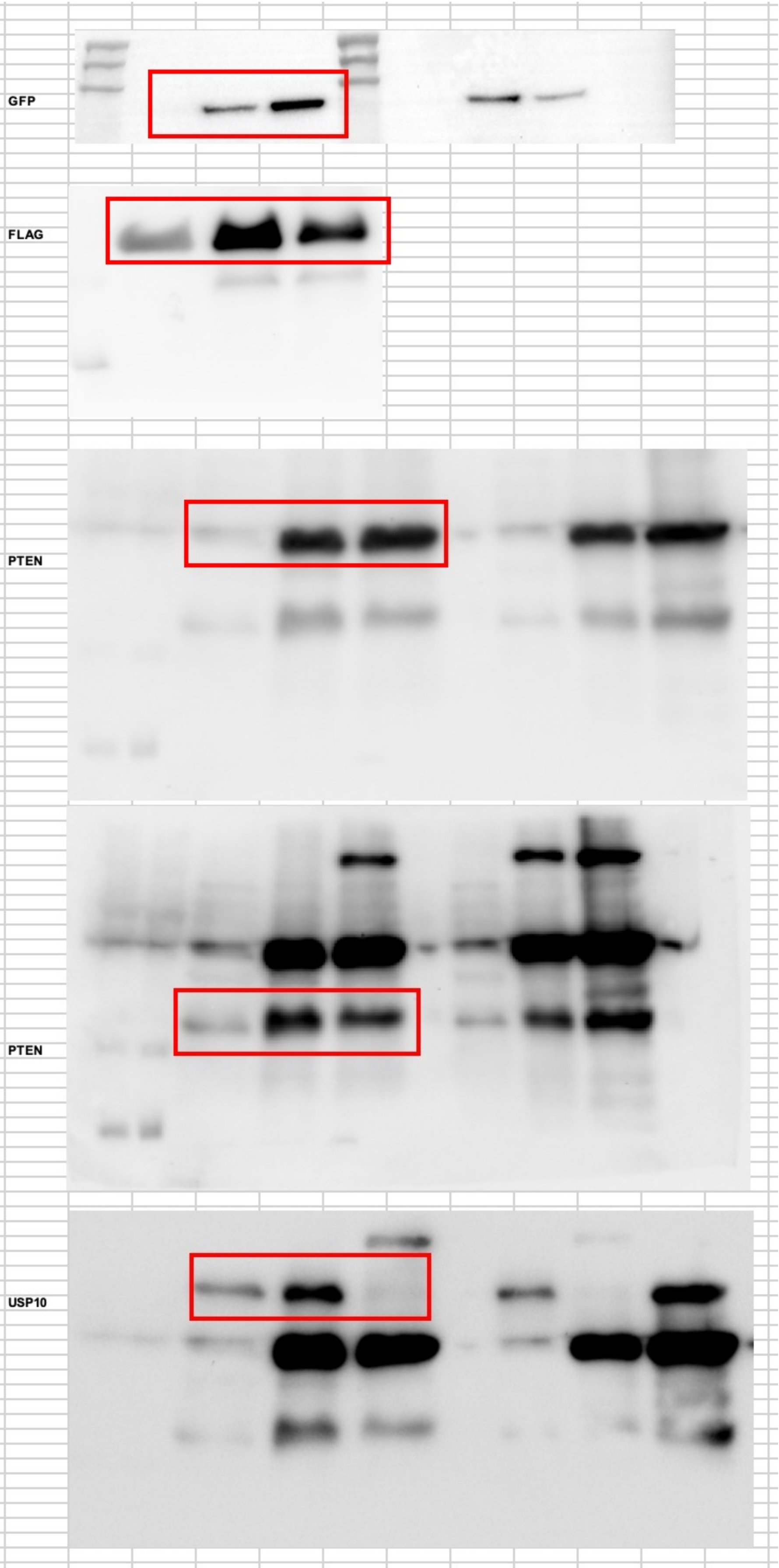

Full unedited gel for Figure 5D

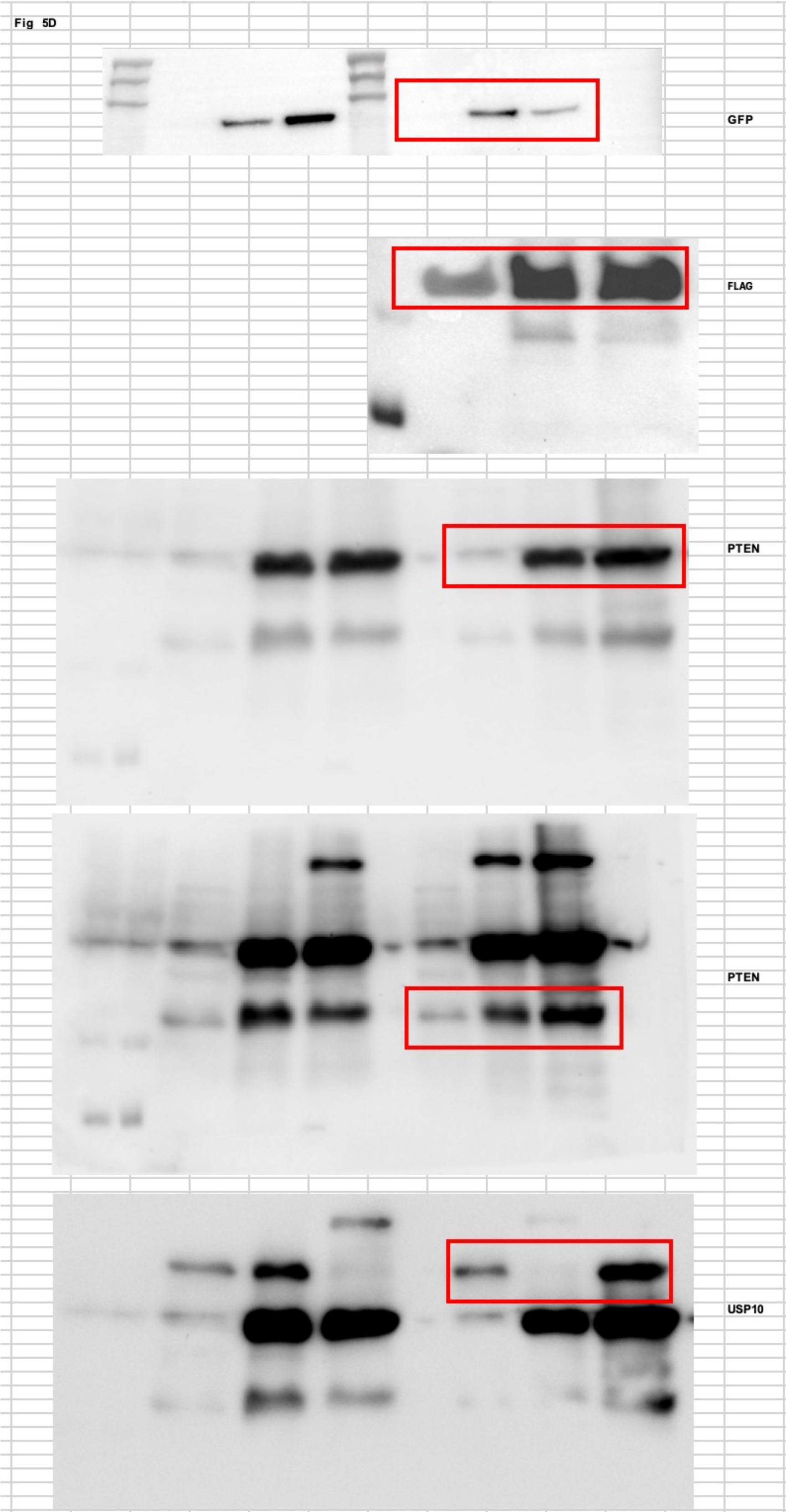

Full unedited gel for Figure 5G

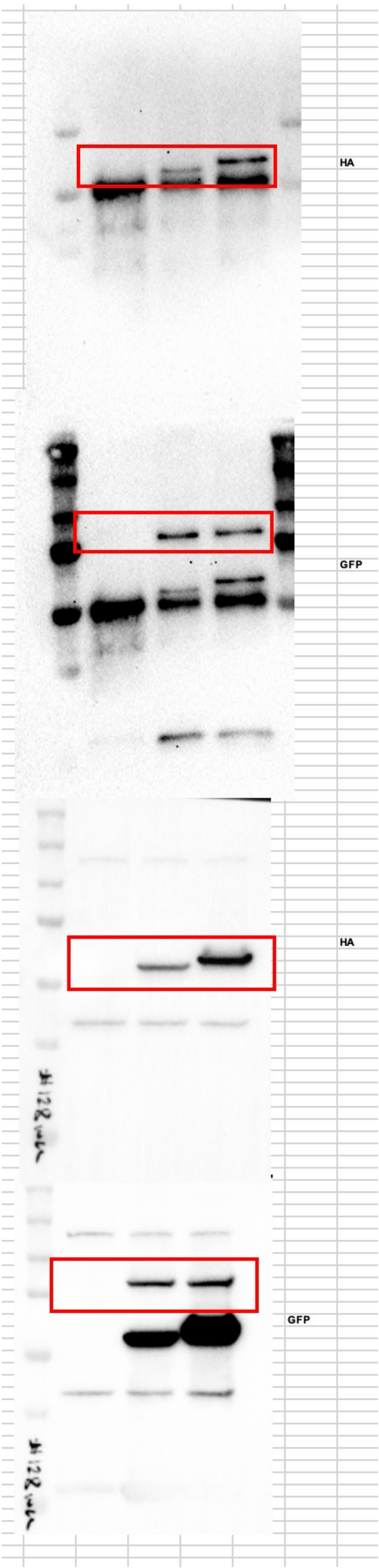

Full unedited gel for Figure 5H

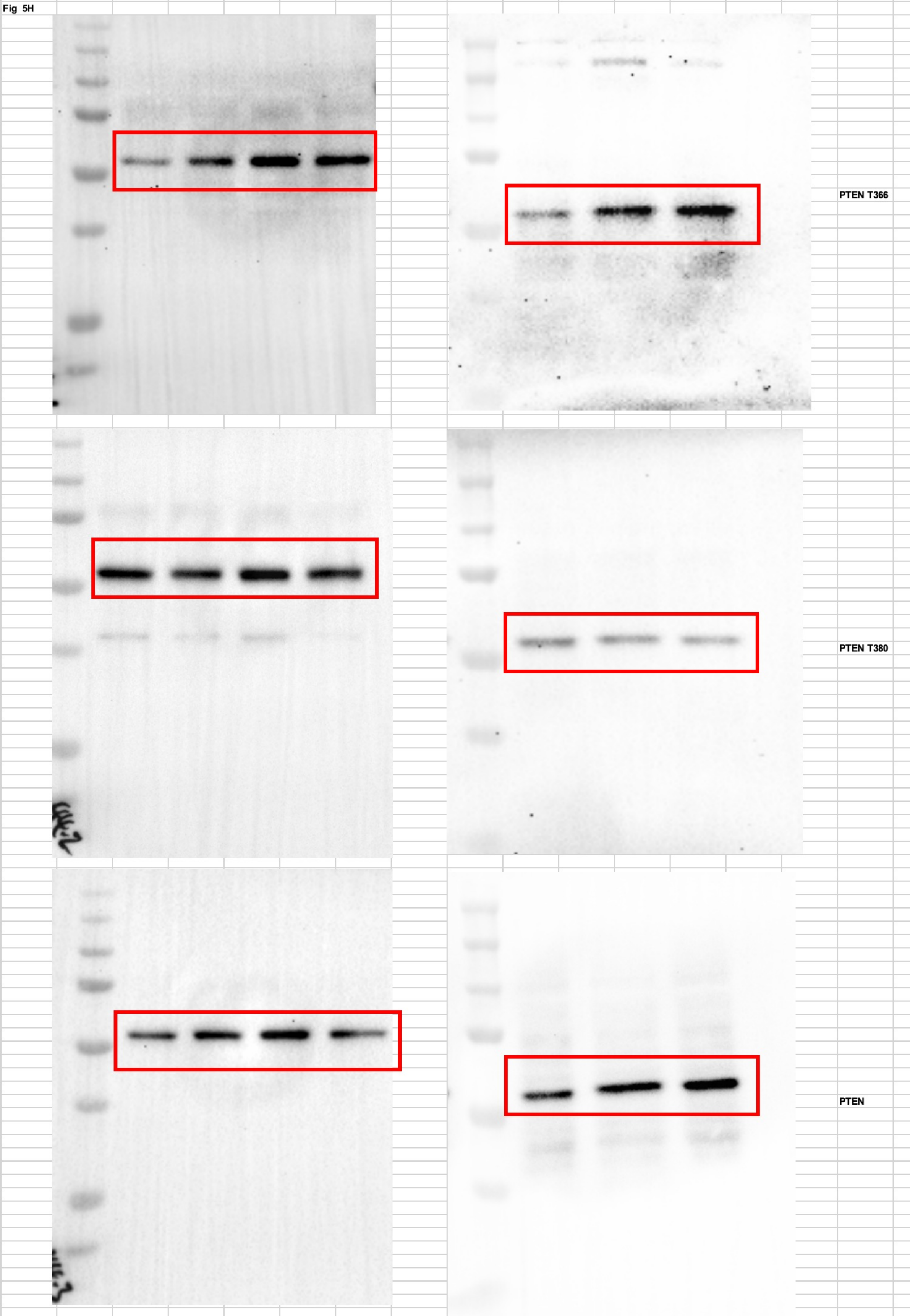

Full unedited gel for Figure 5H

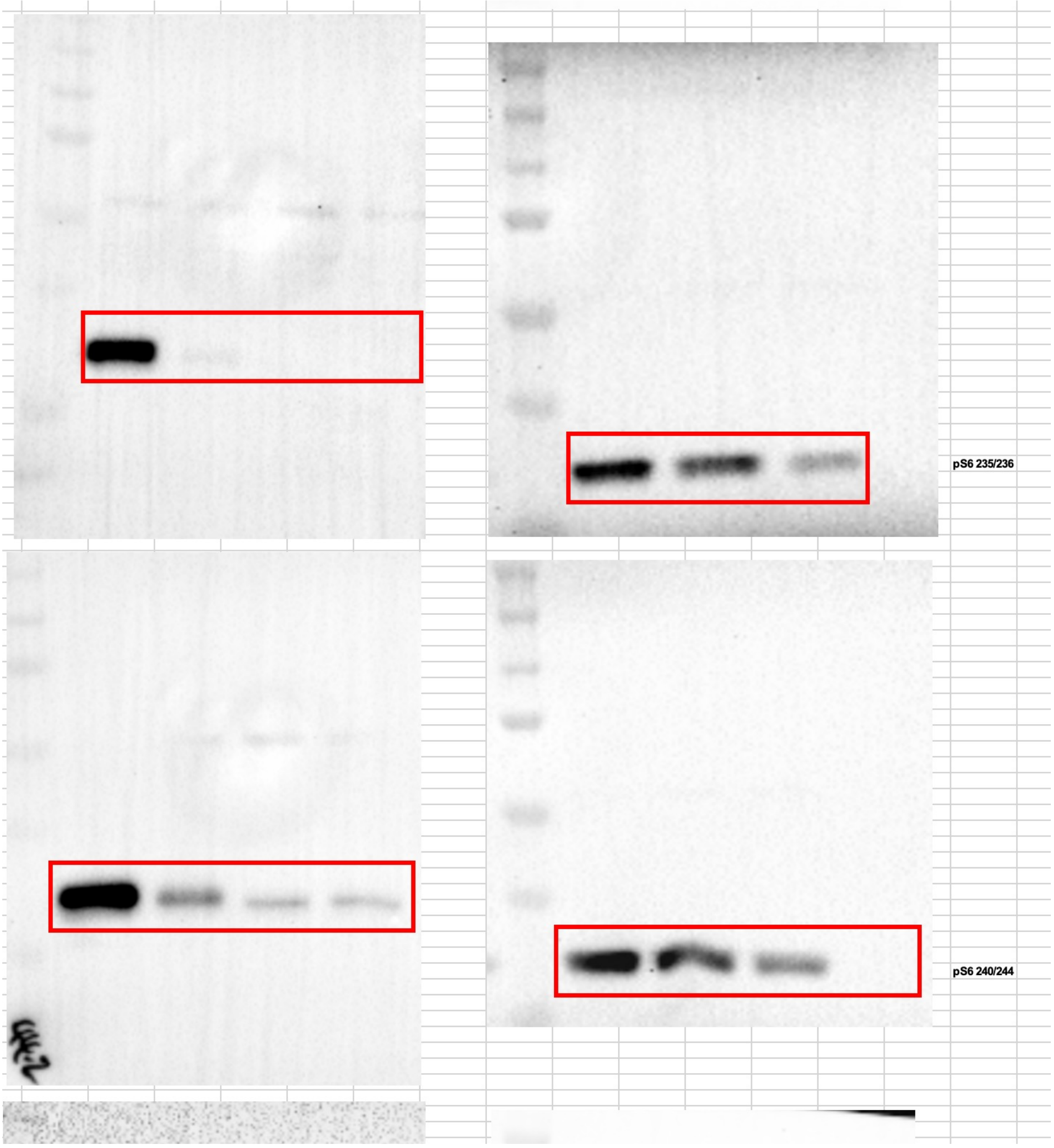

Full unedited gel for Figure 5H

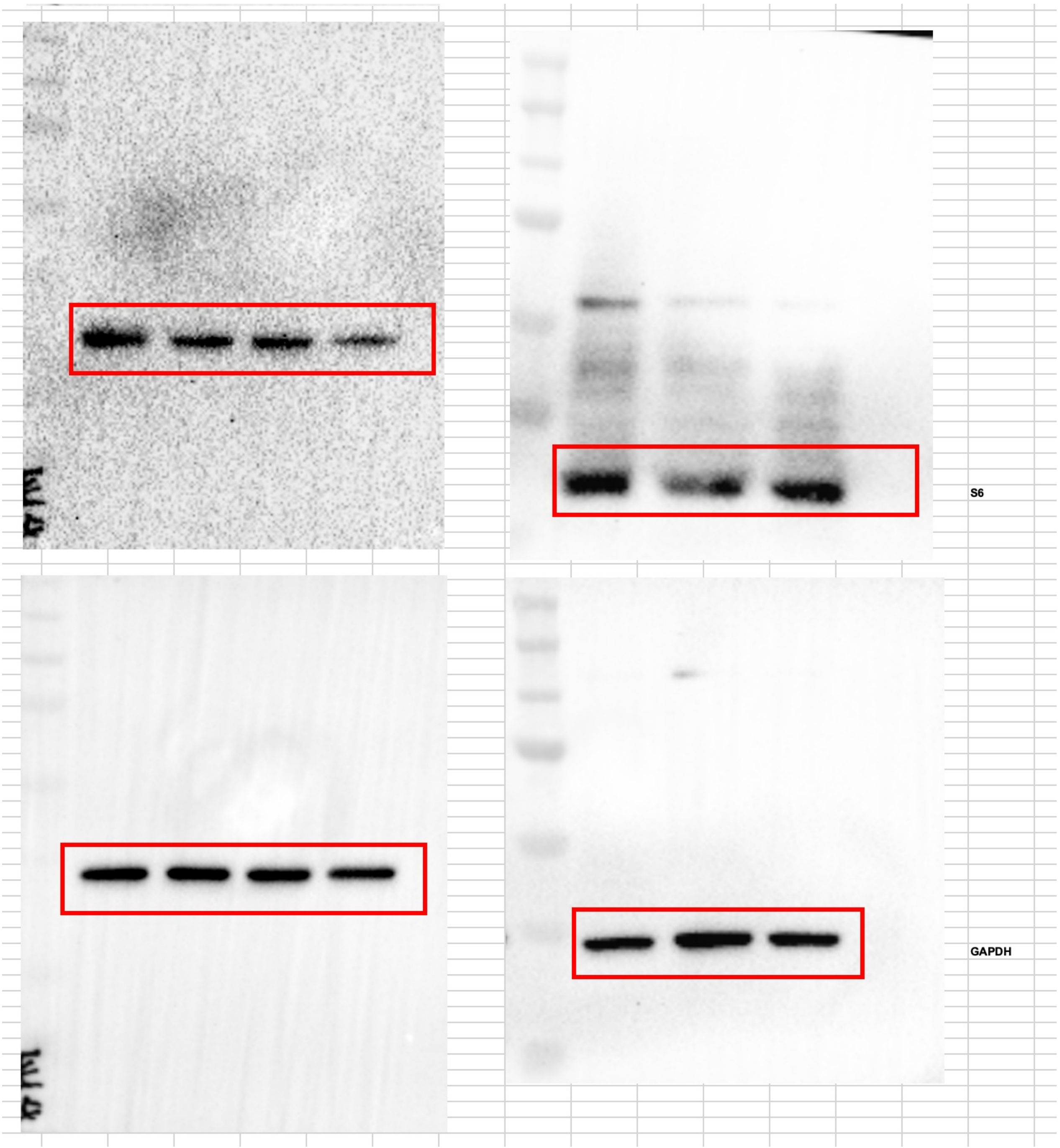

Full unedited gel for Figure 5I

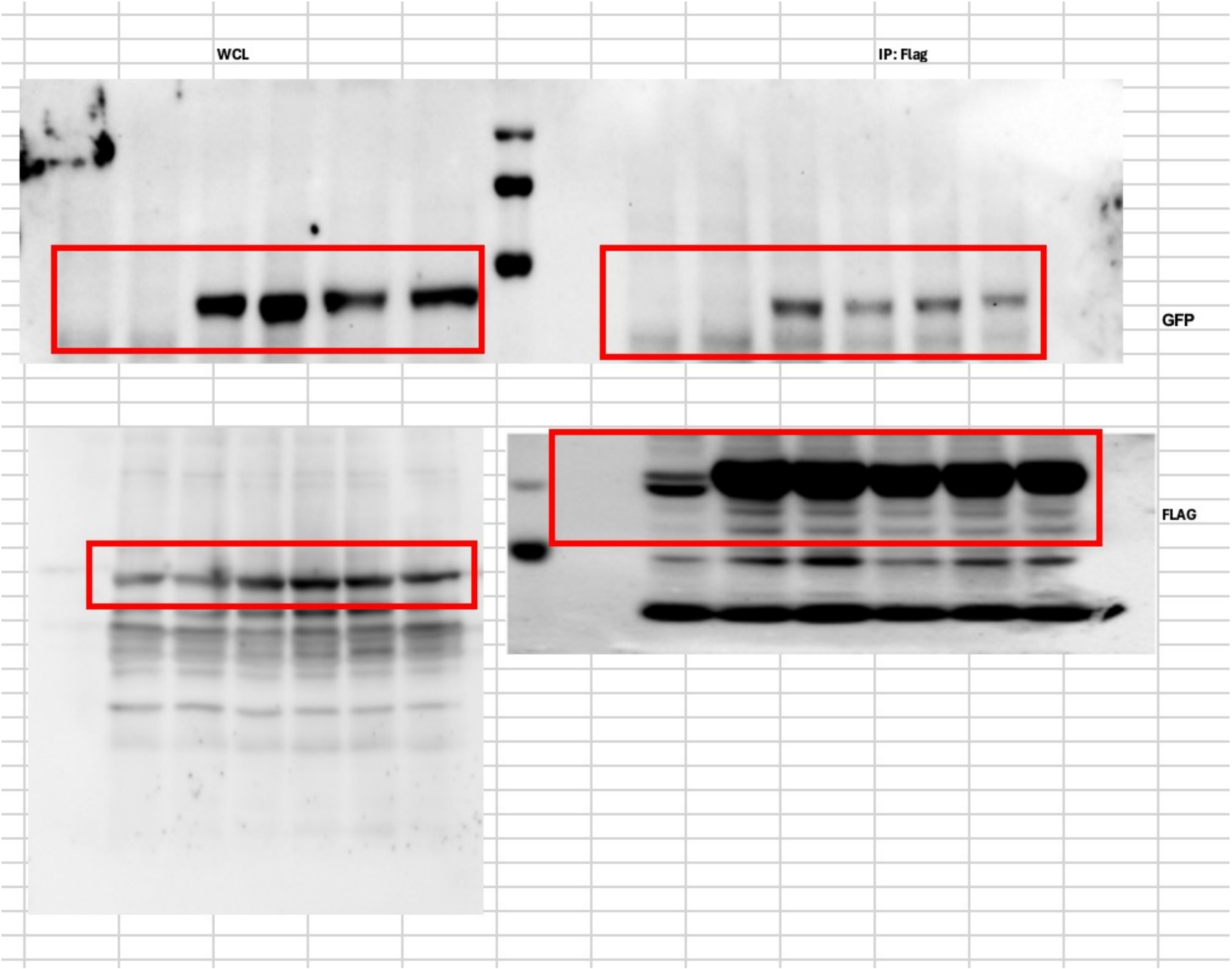

Full unedited gel for Figure 6A

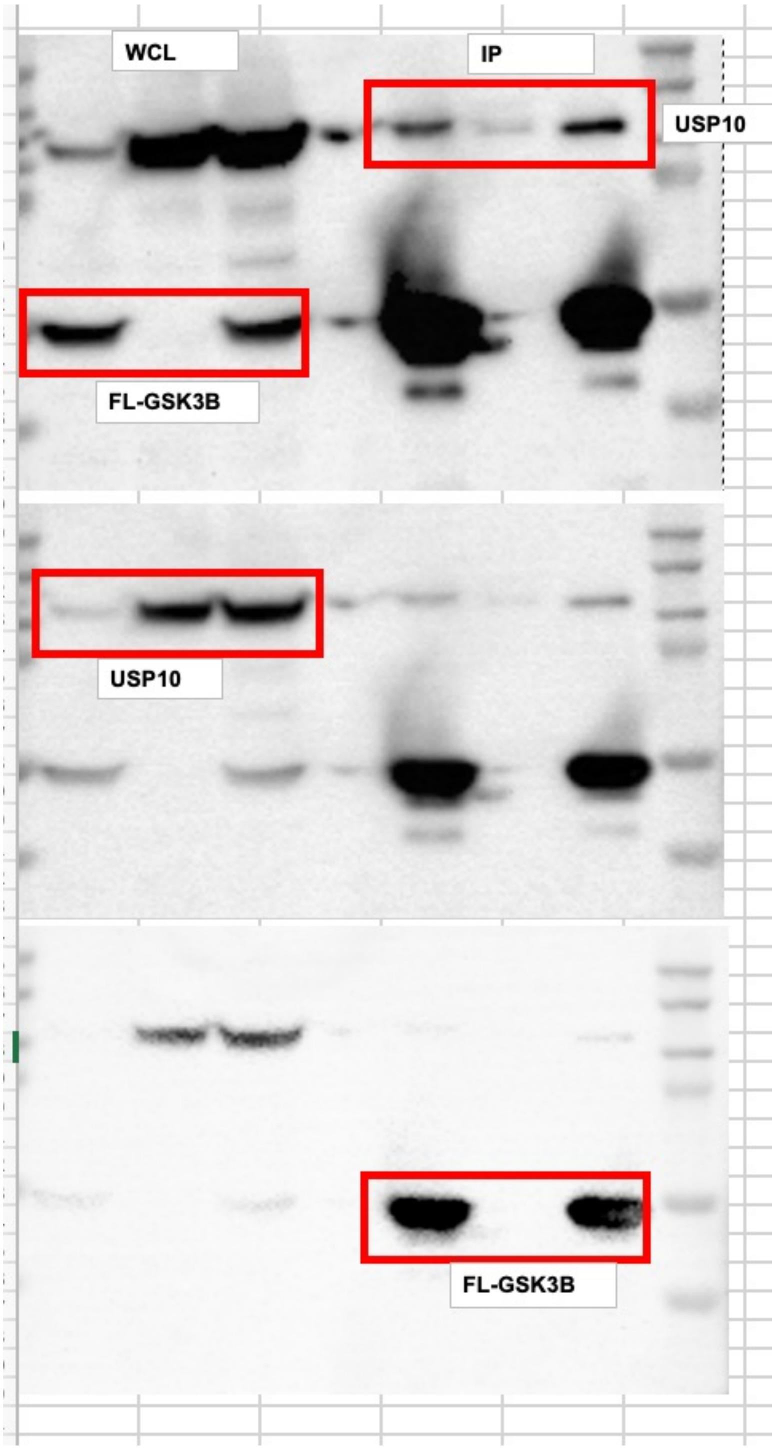

Full unedited gel for Figure 6B

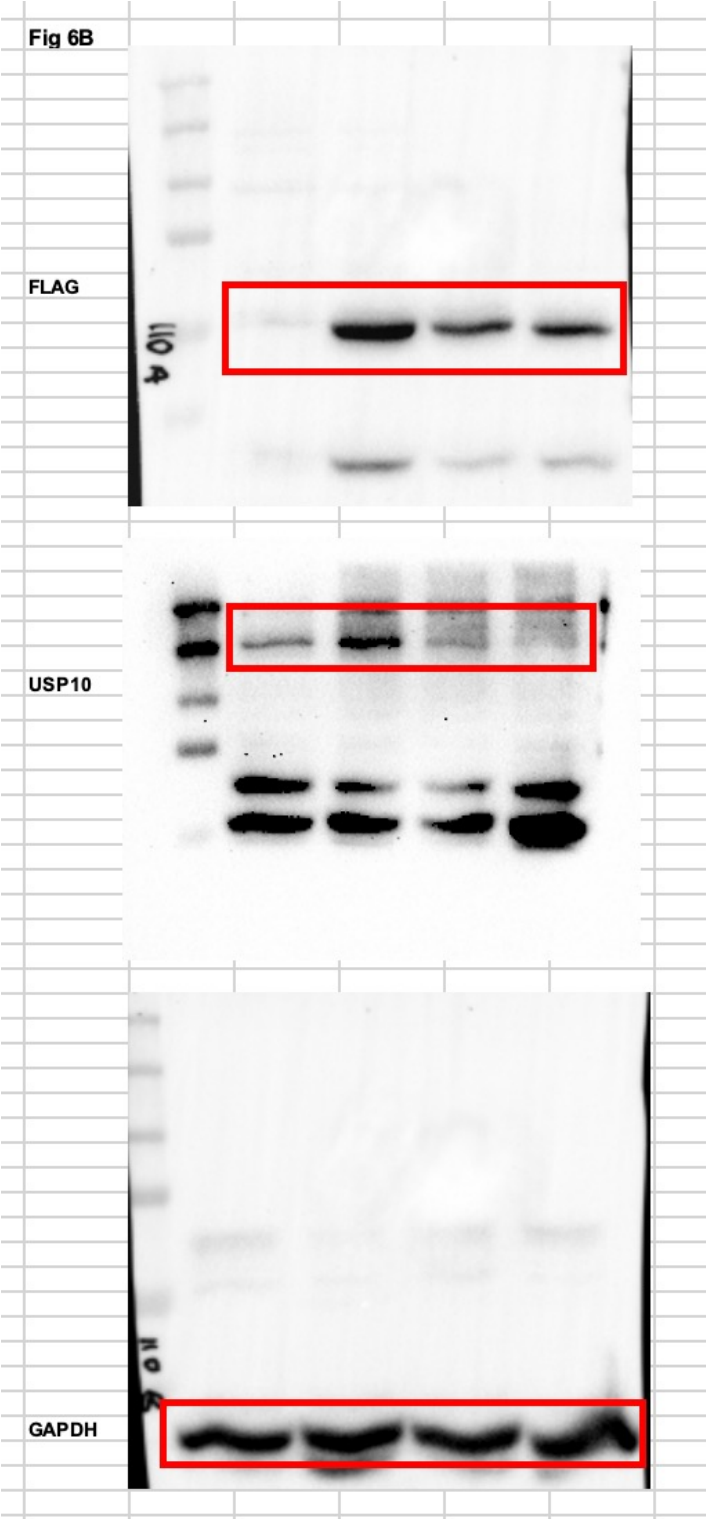

Full unedited gel for Figure 6C

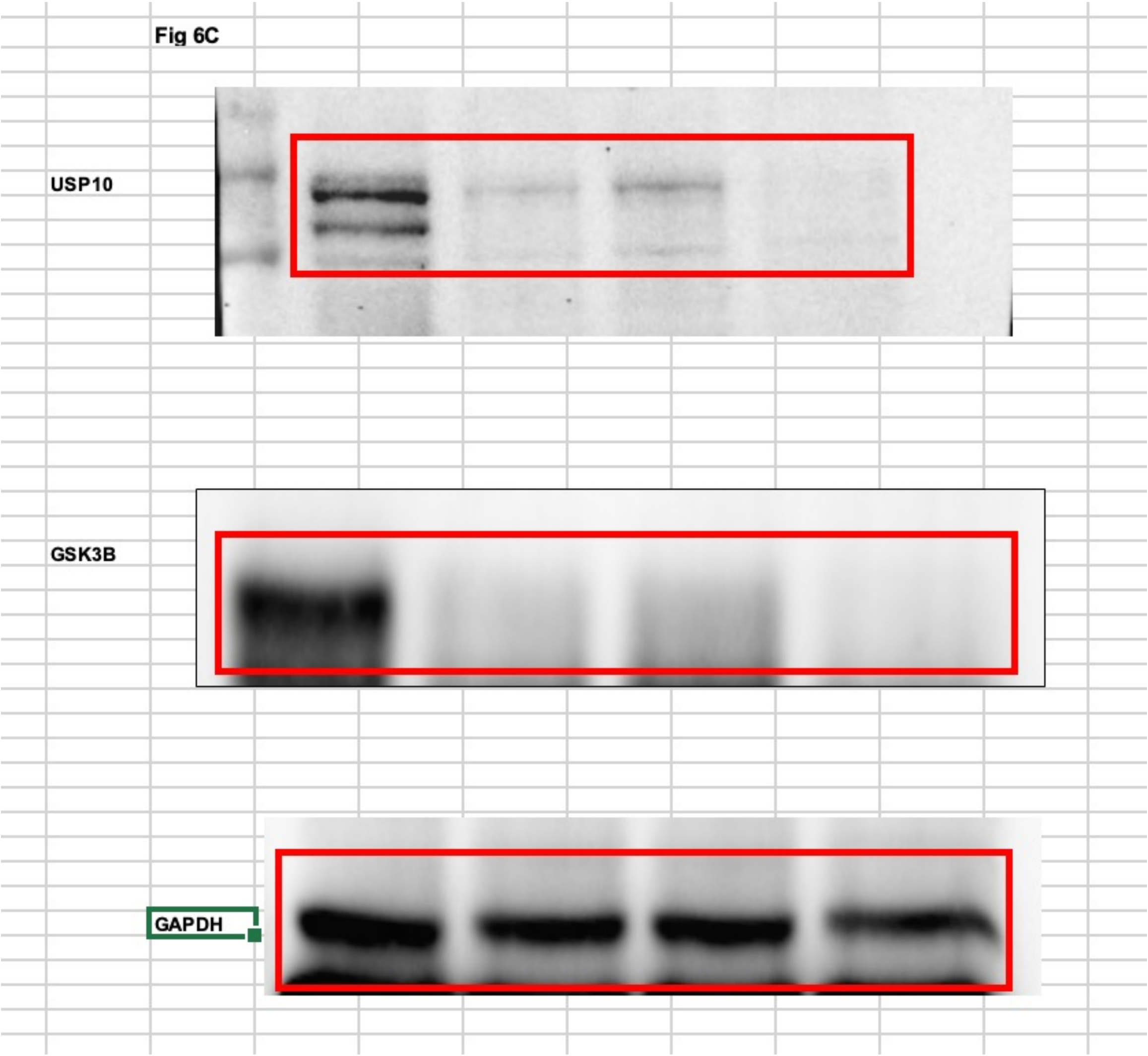

Full unedited gel for Figure 6D

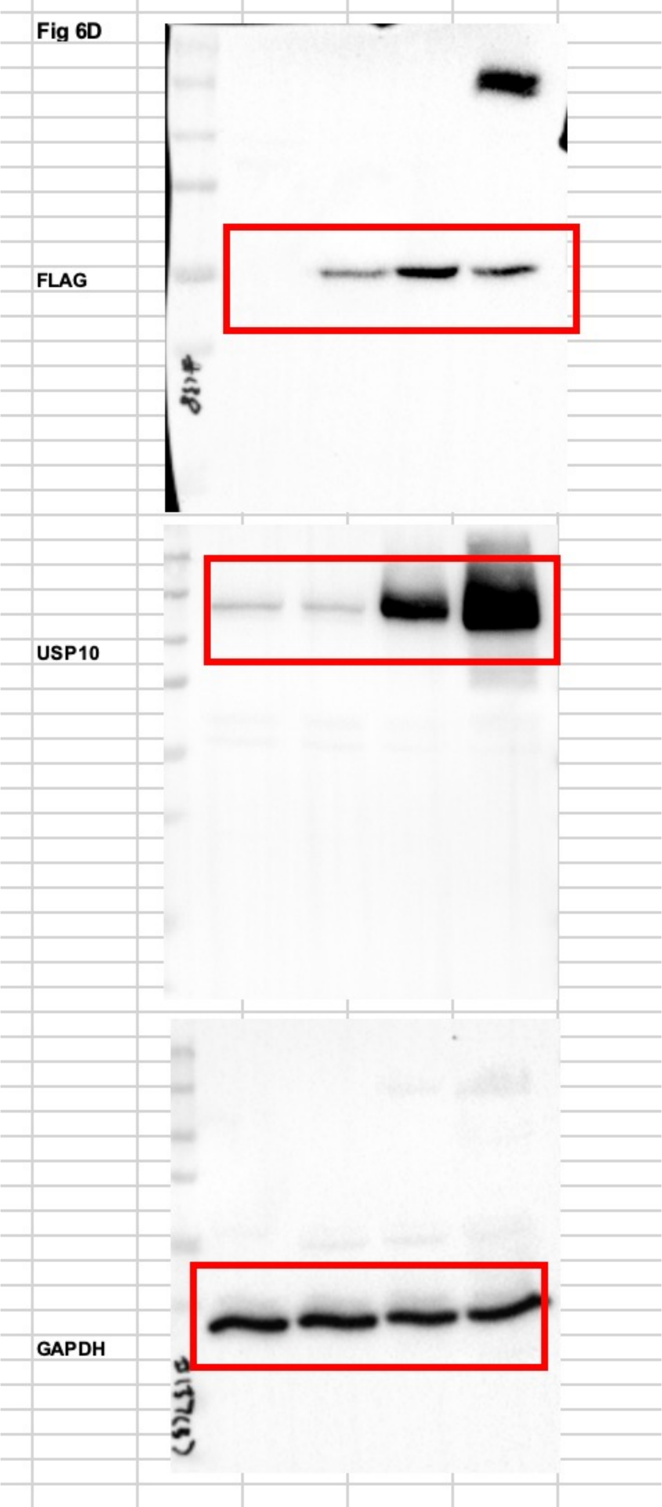

Full unedited gel for Figure 6E

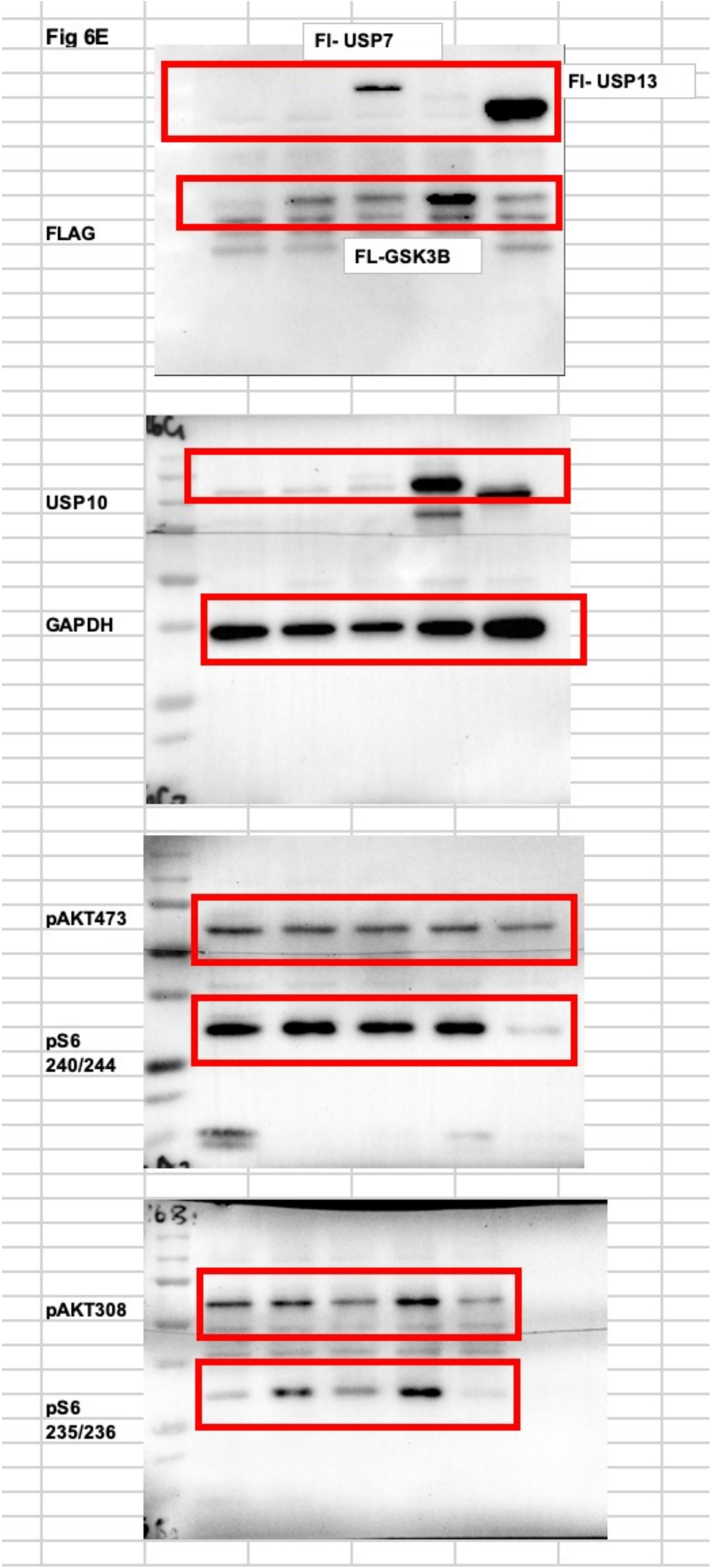

Full unedited gel for Figure 6F

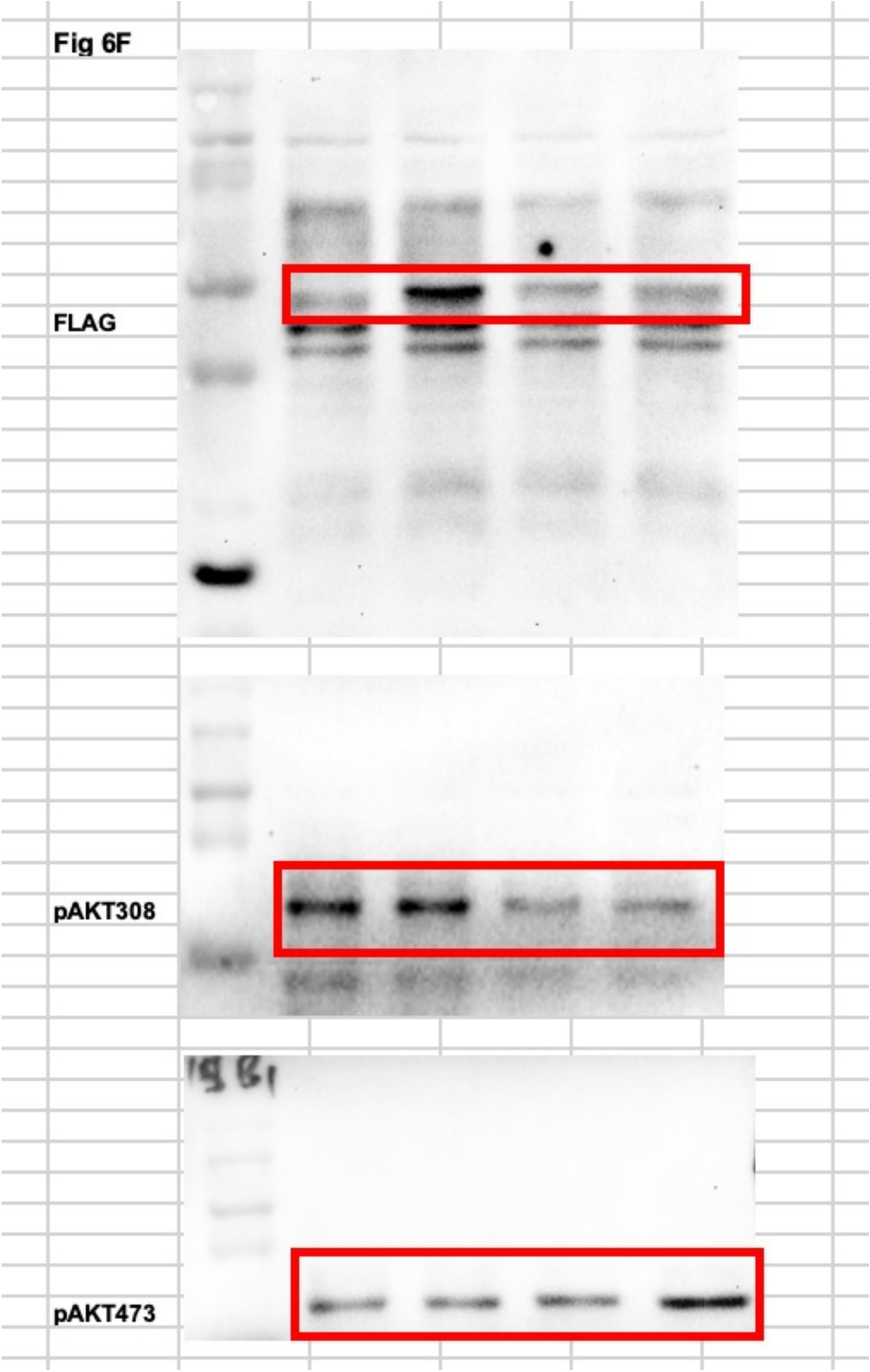

Full unedited gel for Figure 6F

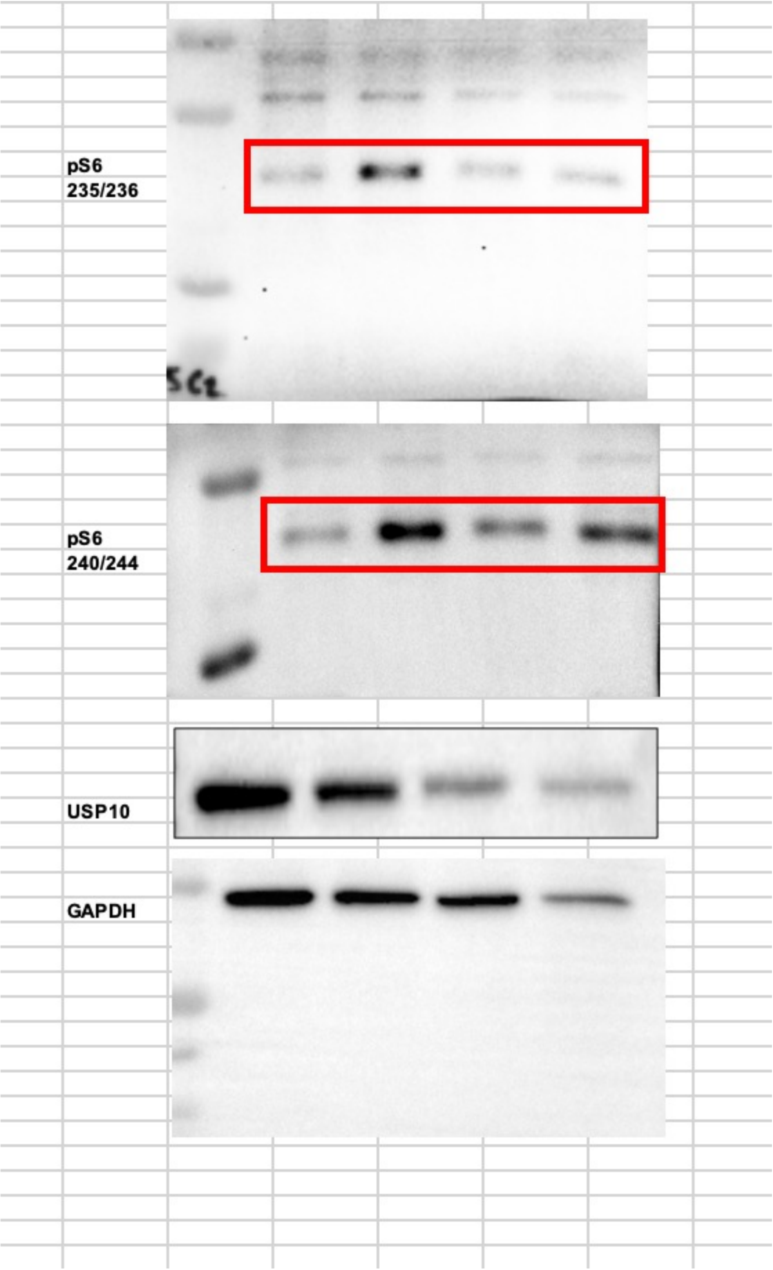

Full unedited gel for Figure 6G

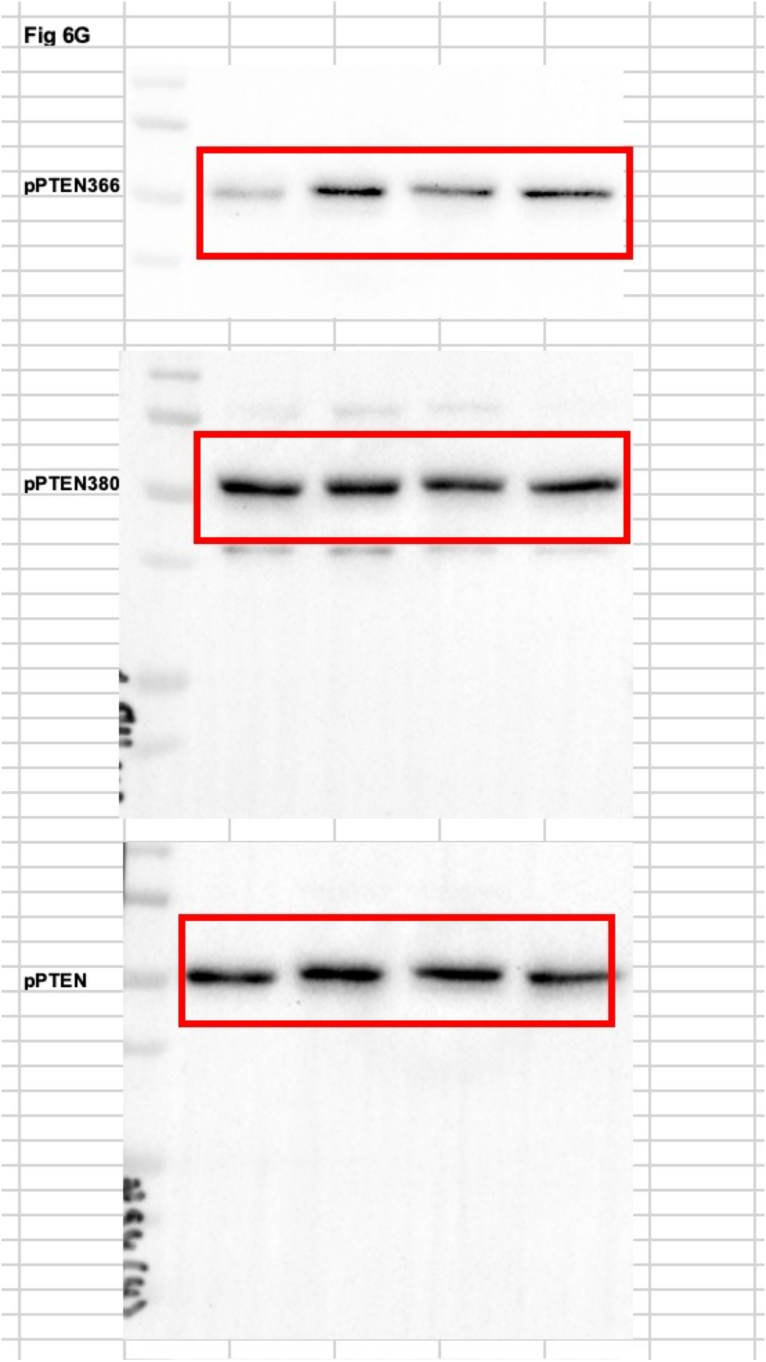

Full unedited gel for Figure 6G

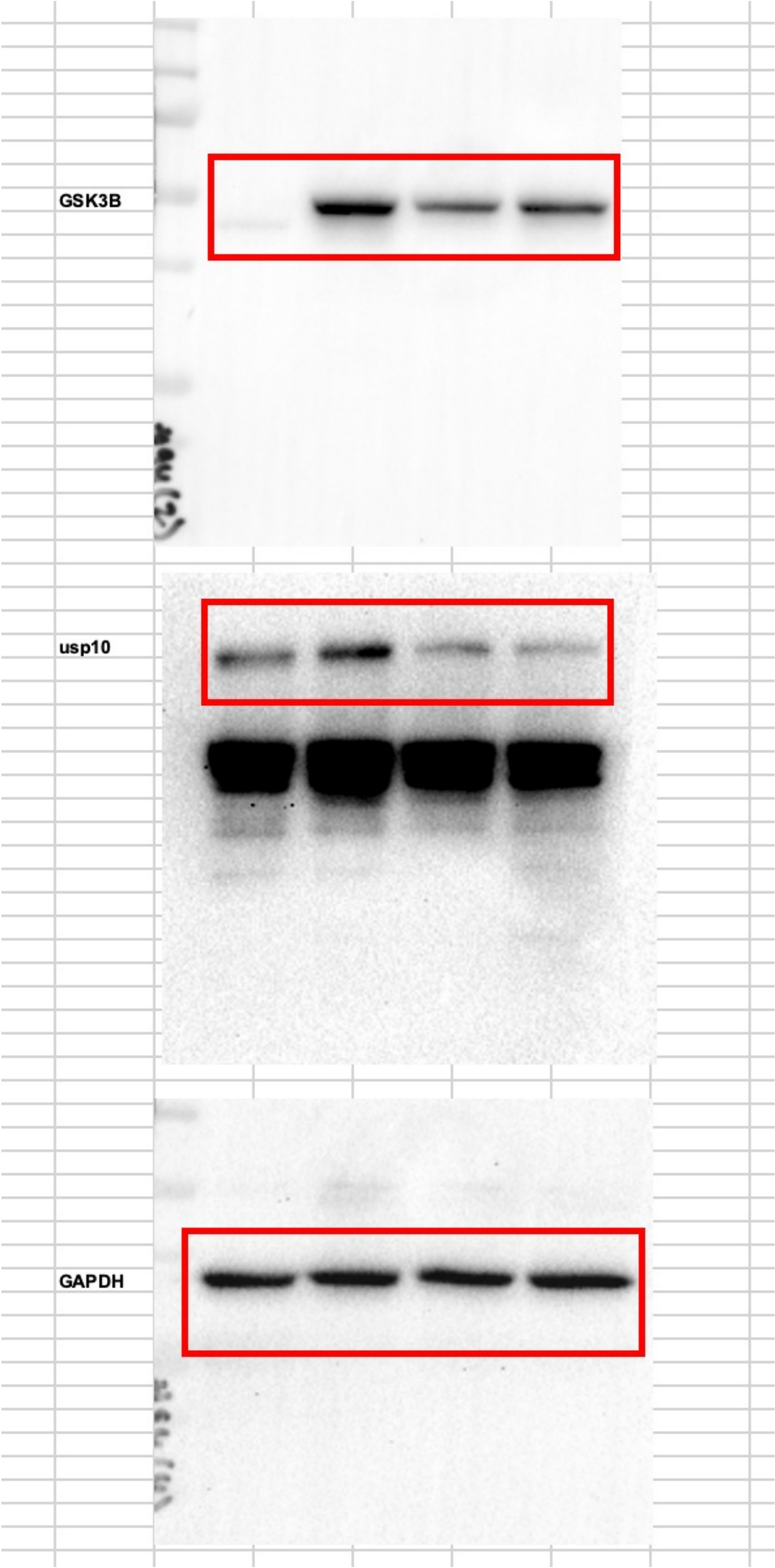

Full unedited gel for Figure 6H

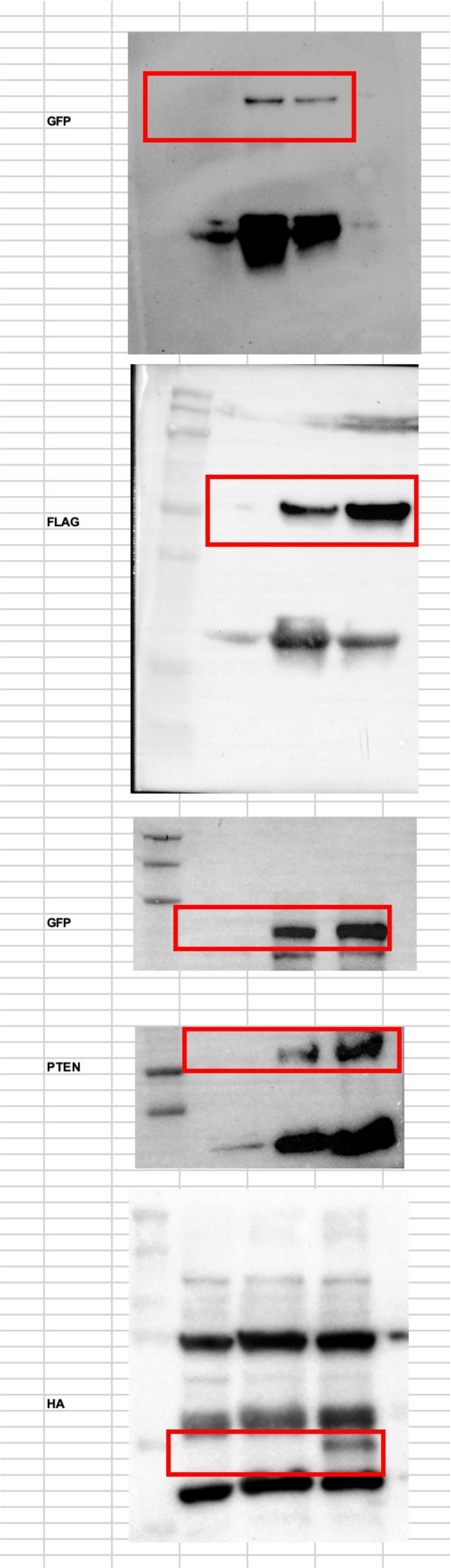

Full unedited gel for Figure 6I

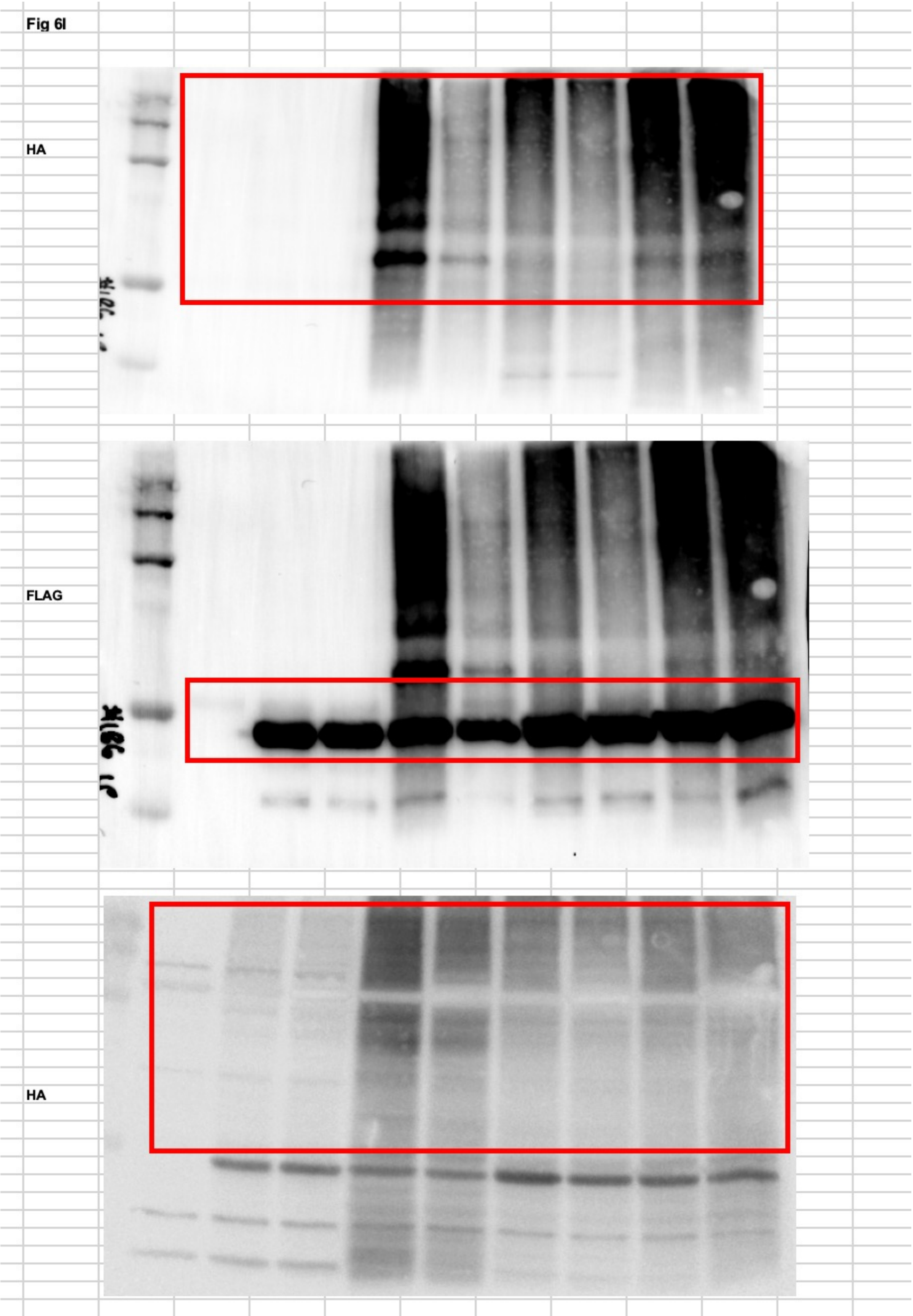

Full unedited gel for Figure 6I

FLAG

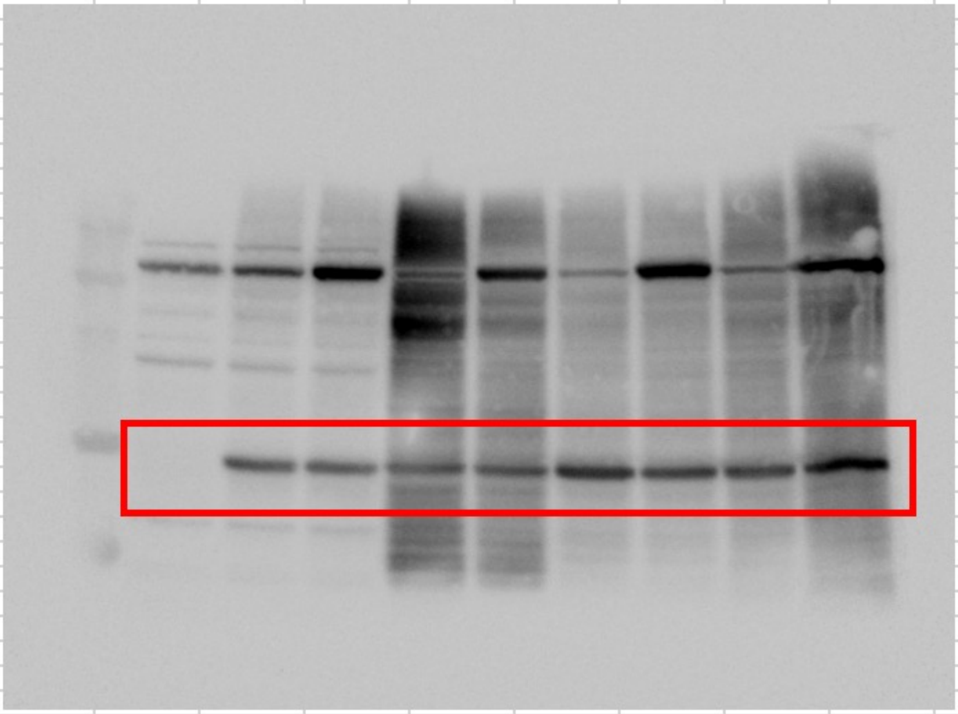

USP10

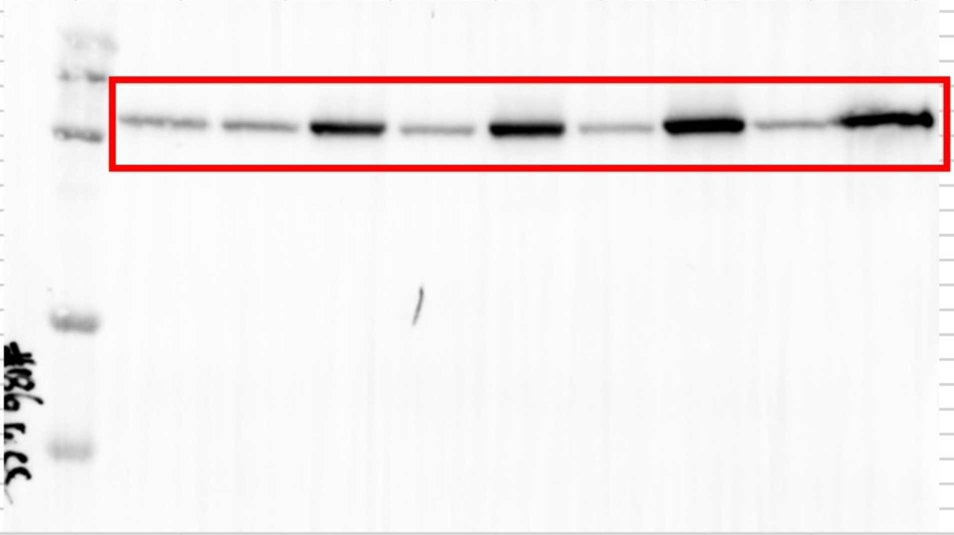

Full unedited gel for Figure 7G

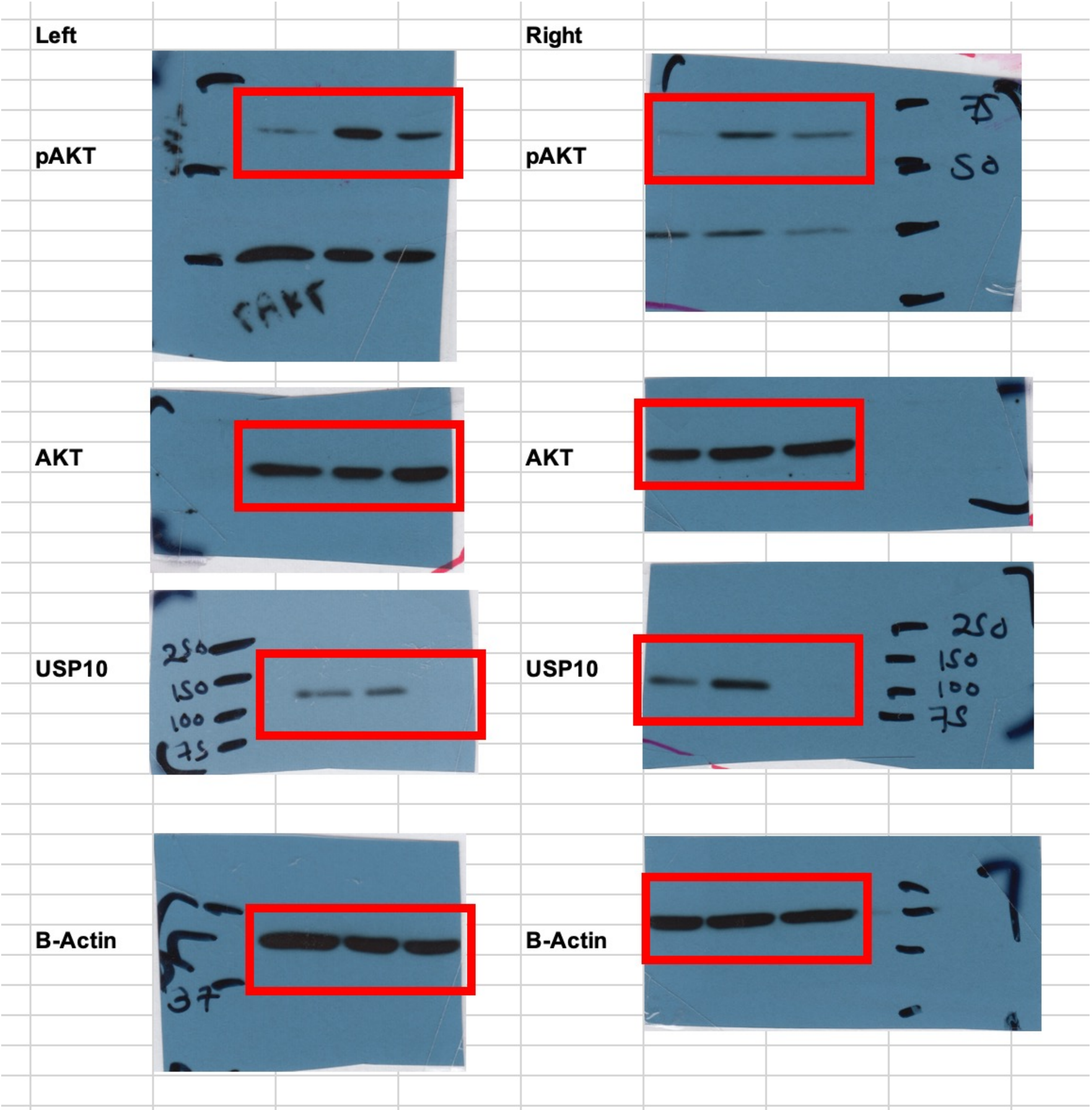

Full unedited gel for Supplementary Figure 8J

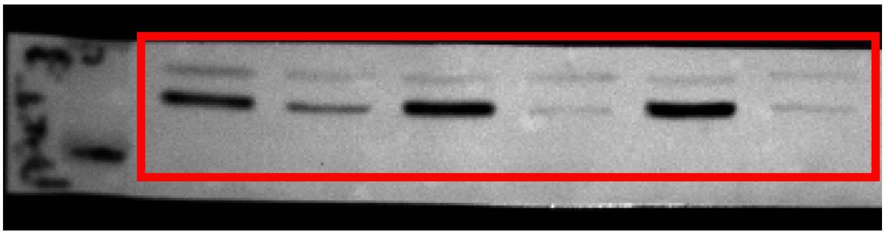

pAKT 473

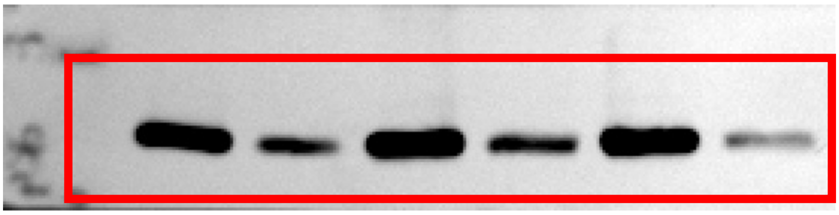

pS6 240/244

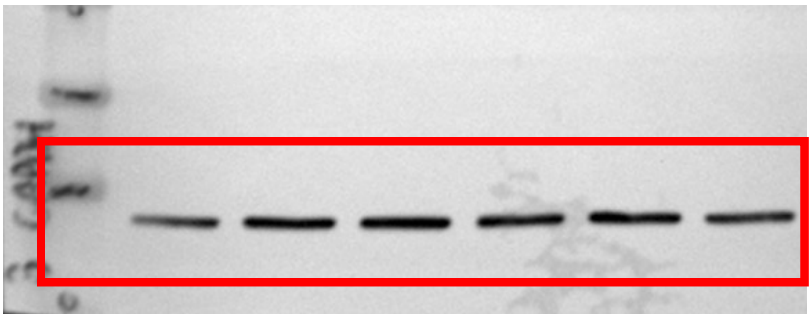

GAPDH

Full unedited gel for Supplementary Figure 3A

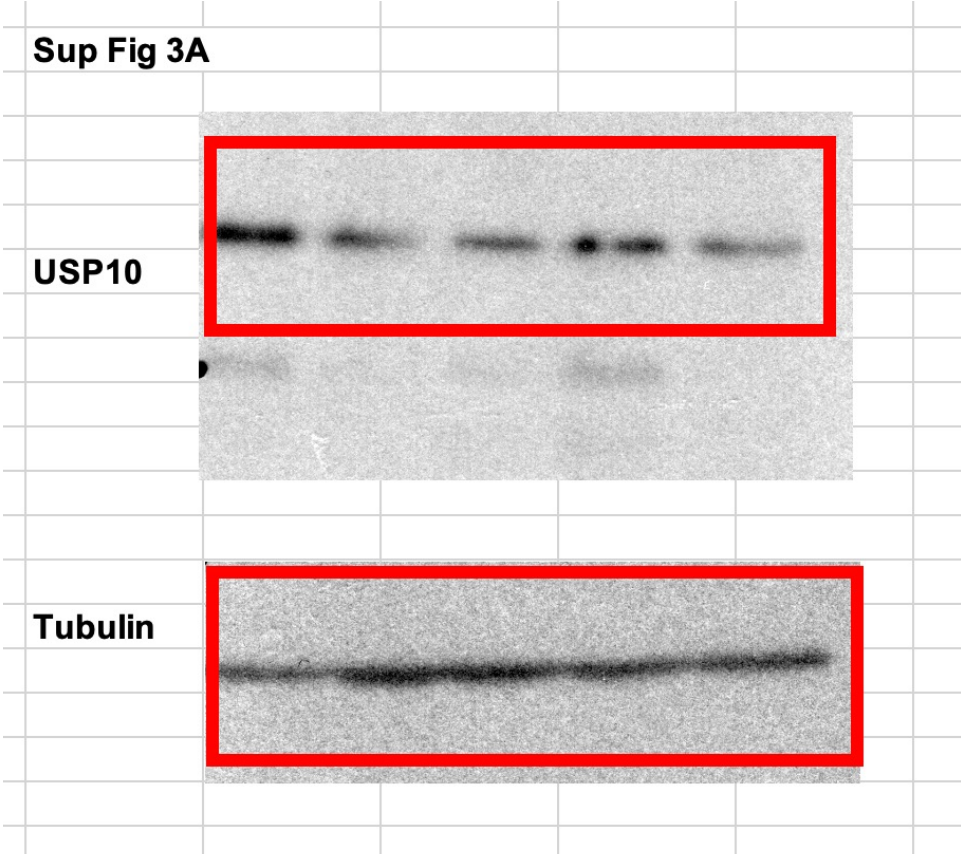

Full unedited gel for Supplementary Figure 3C

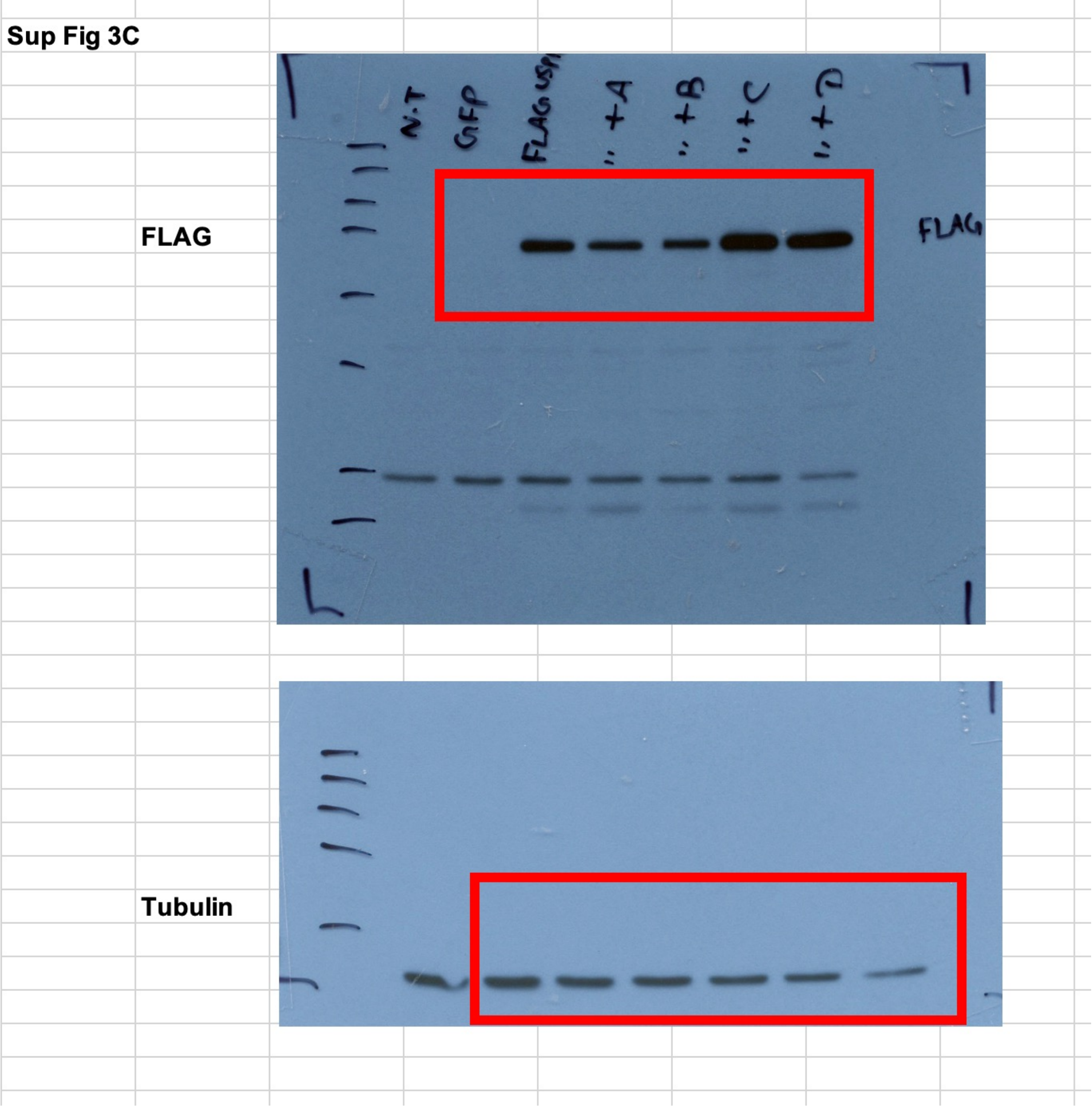

Full unedited gel for Supplementary Figure 3D

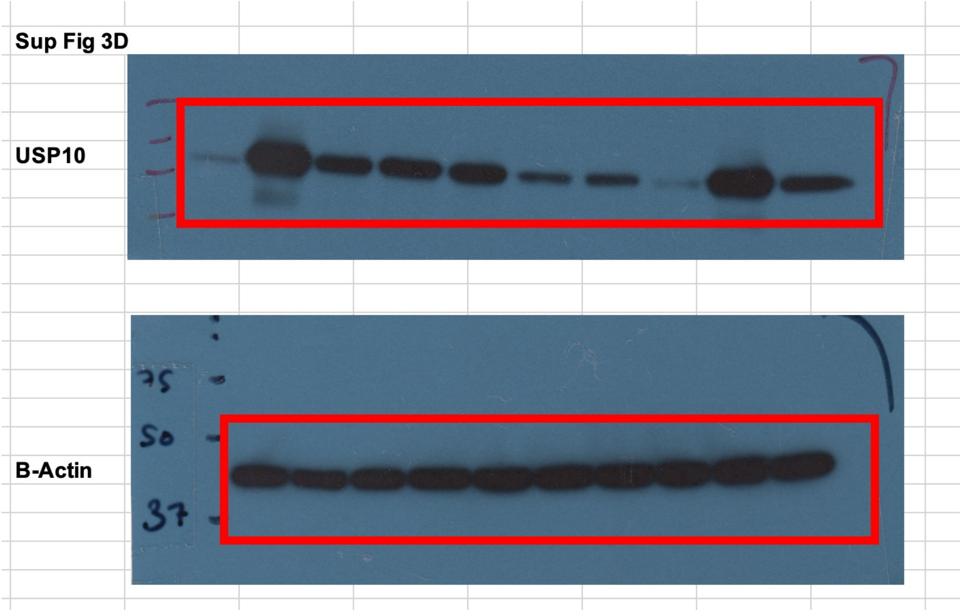

Full unedited gel for Supplementary Figure 3E

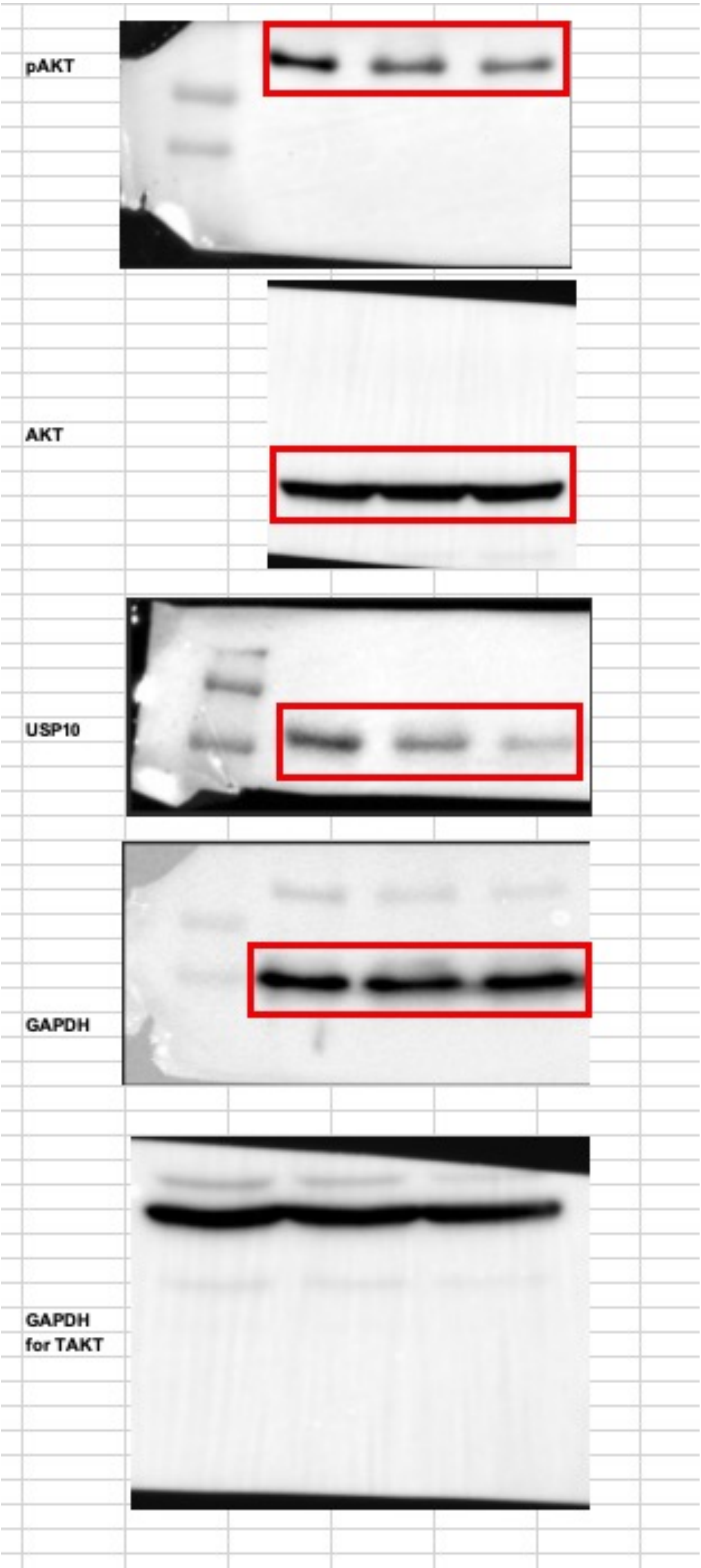

Full unedited gel for Supplementary Figure 9A

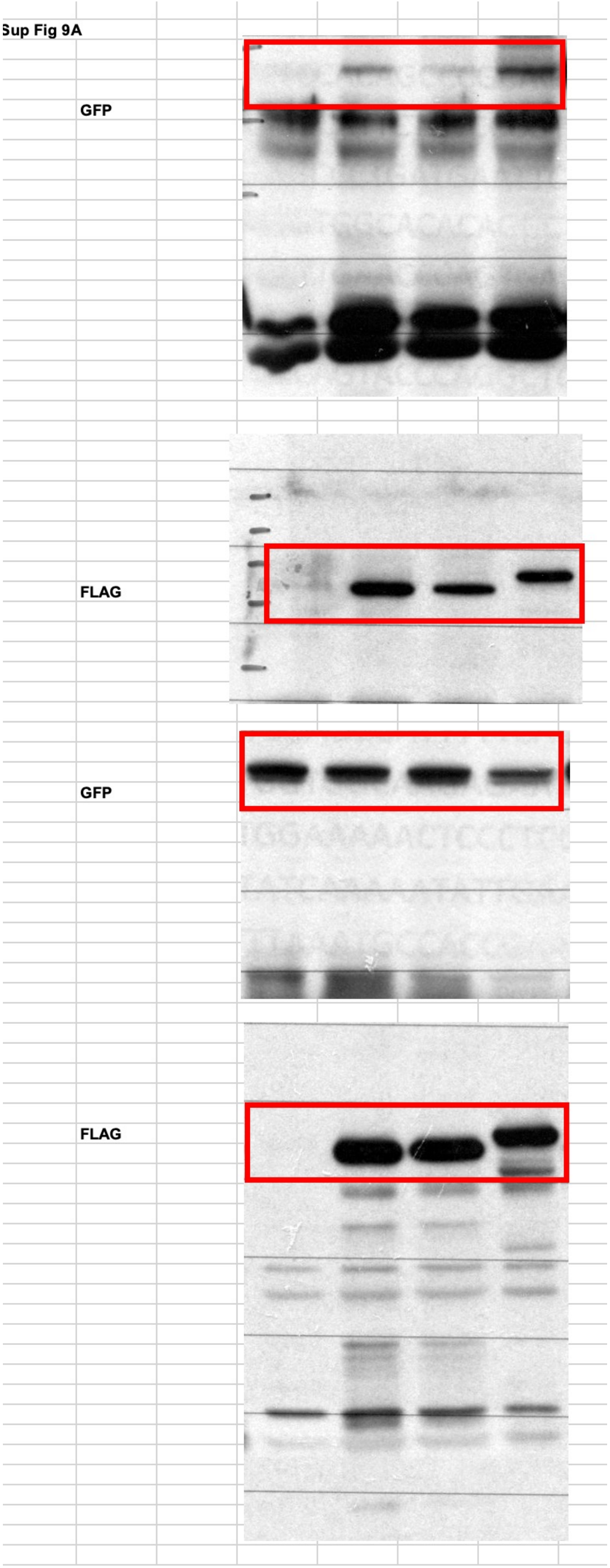

Full unedited gel for Supplementary Figure 9B

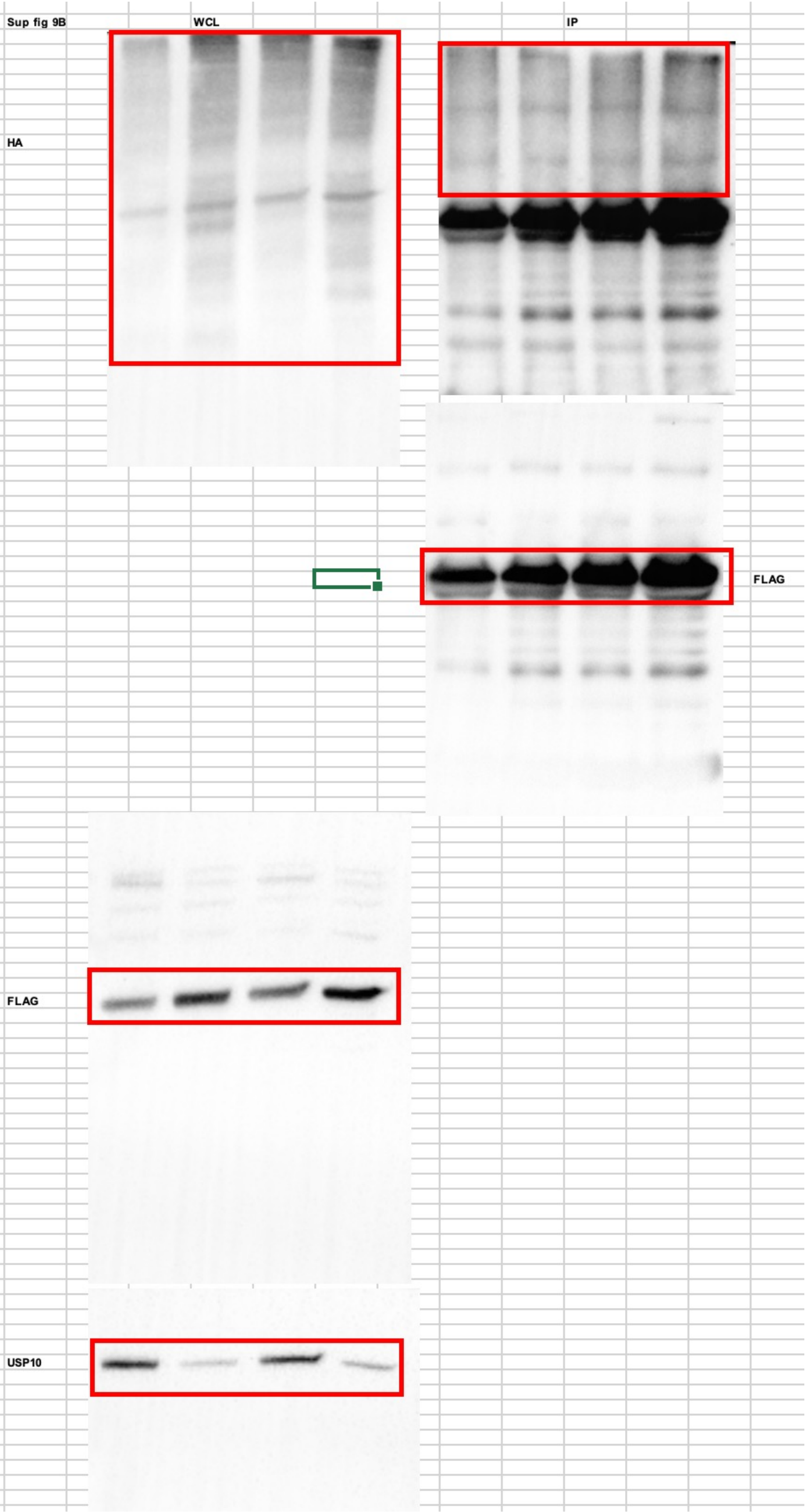

Full unedited gel for Supplementary Figure 9C

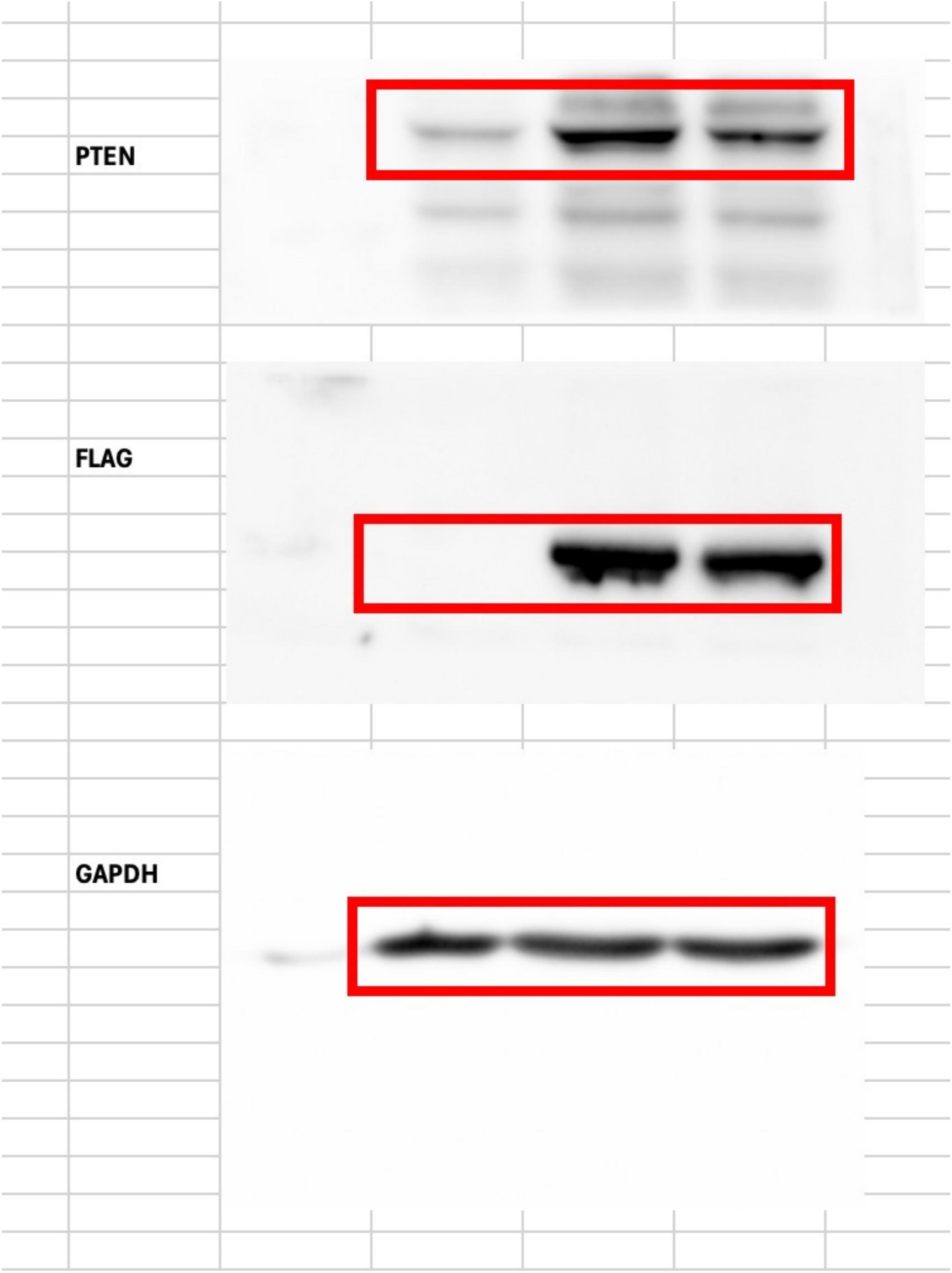

Full unedited gel for Supplementary Figure 9D

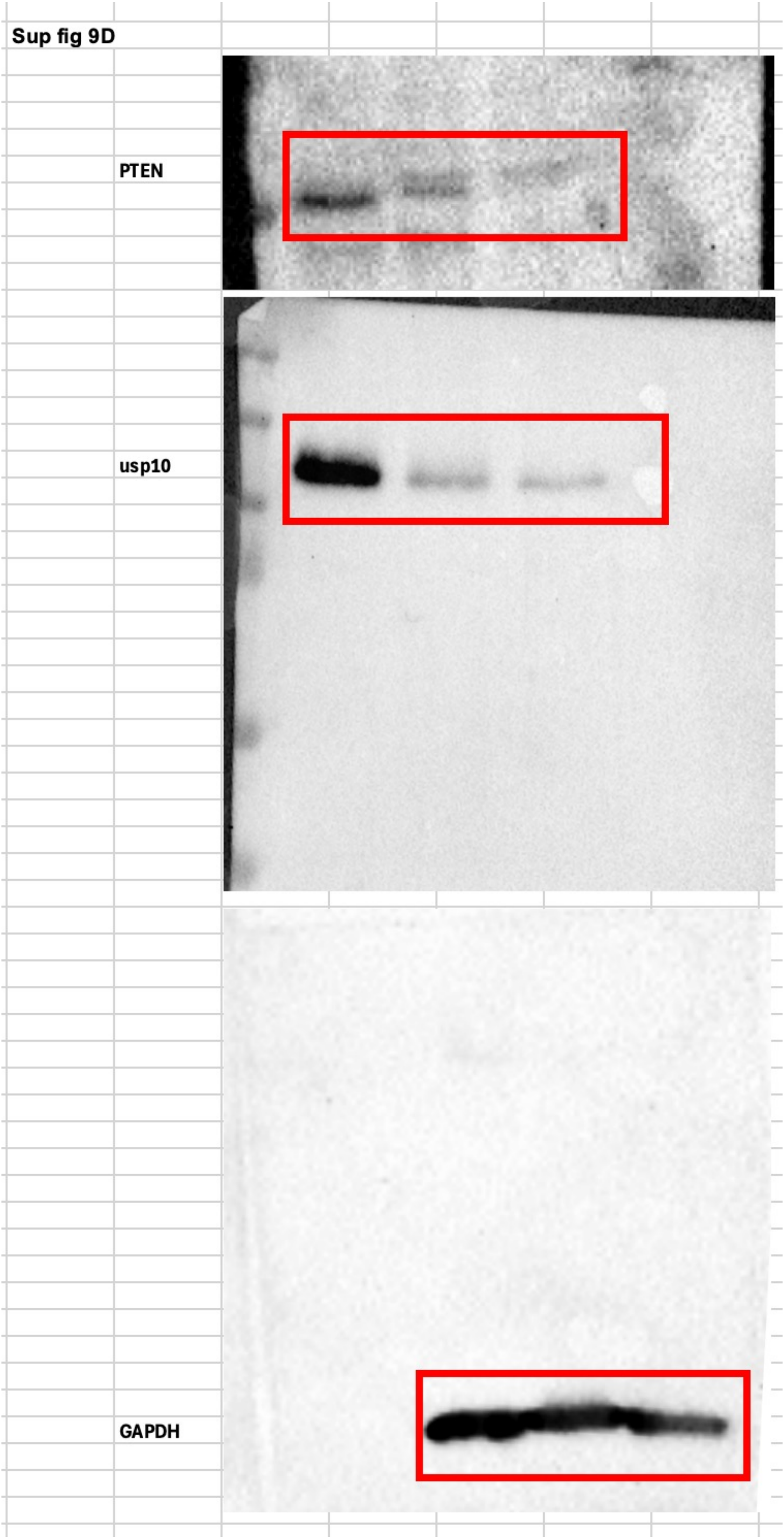

Full unedited gel for Supplementary Figure 9E

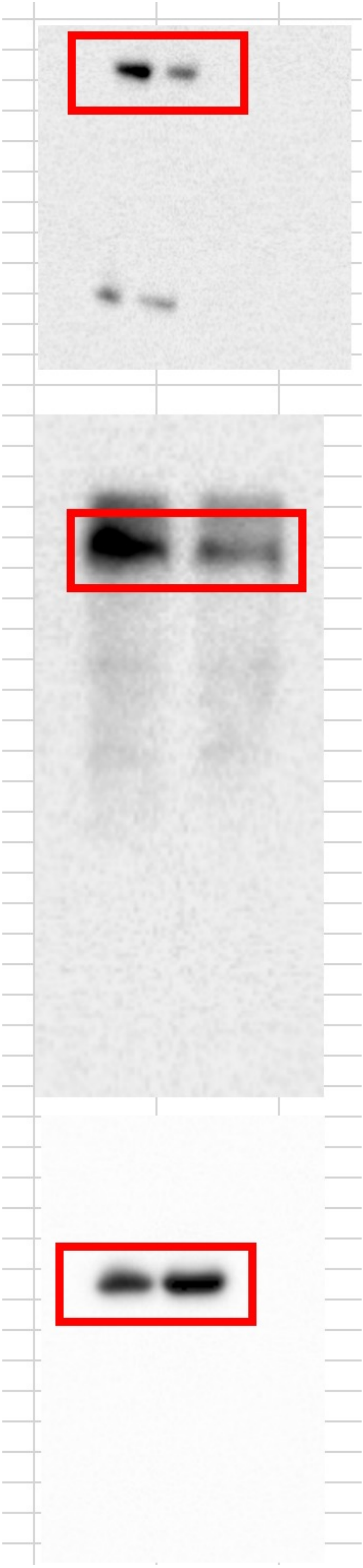

Full unedited gel for Supplementary Figure 9F

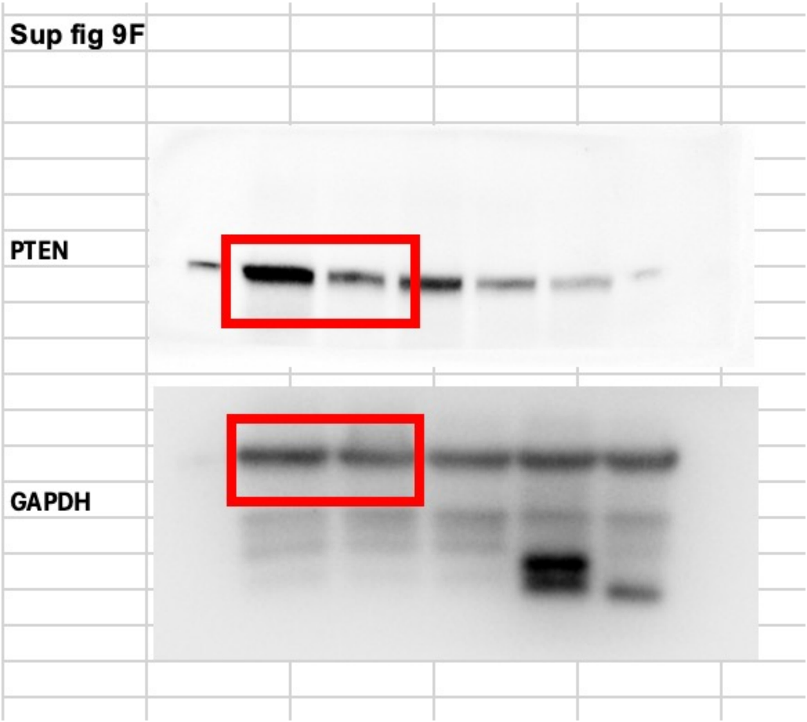

Full unedited gel for Supplementary Figure 9G

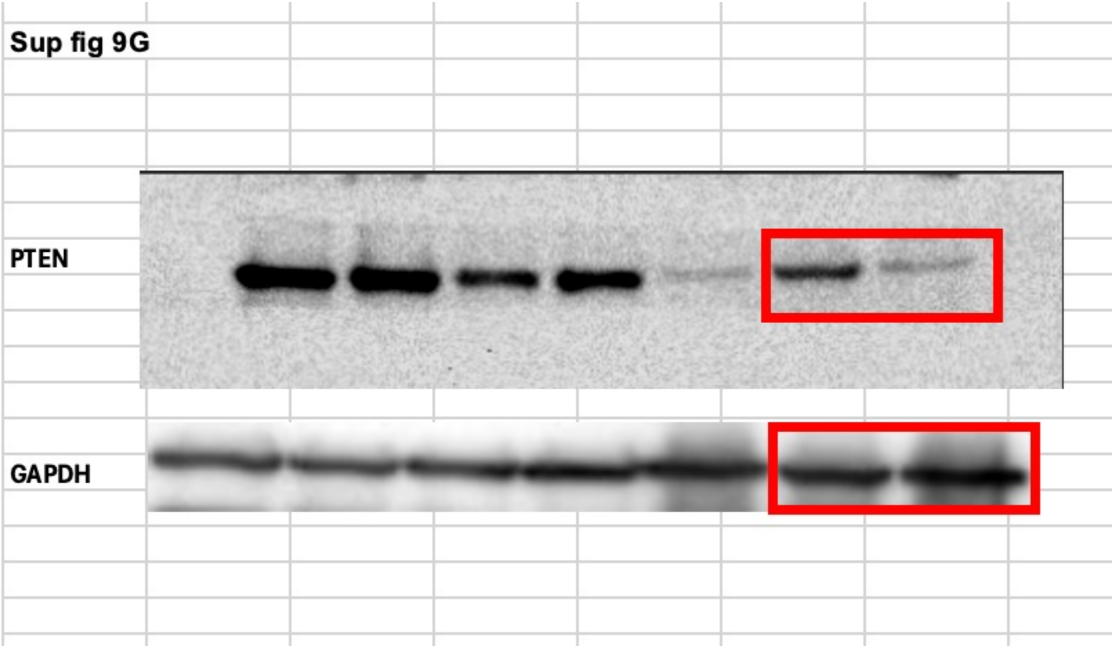

Full unedited gel for Supplementary Figure 9H

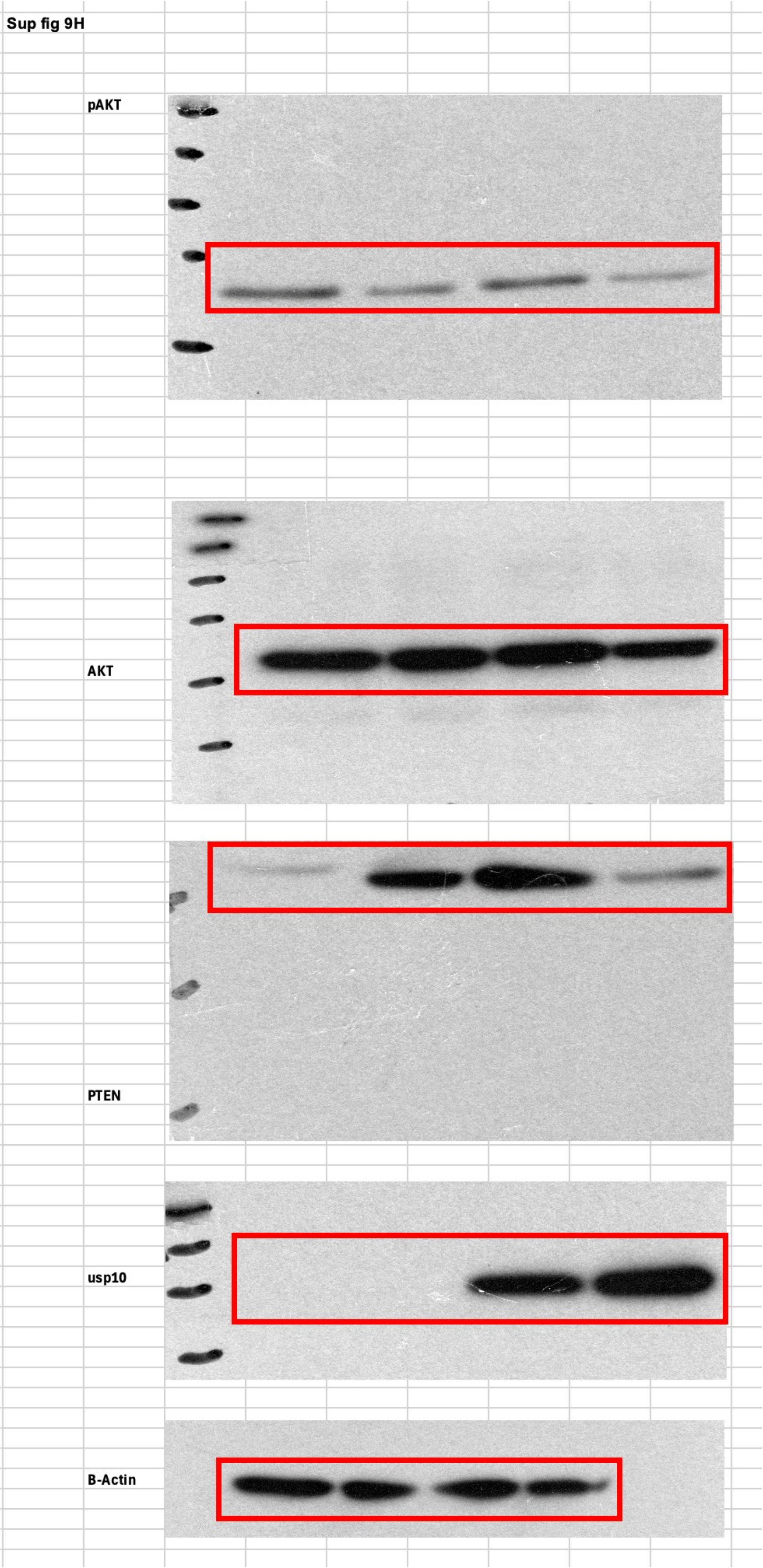

Full unedited gel for Supplementary Figure 9J

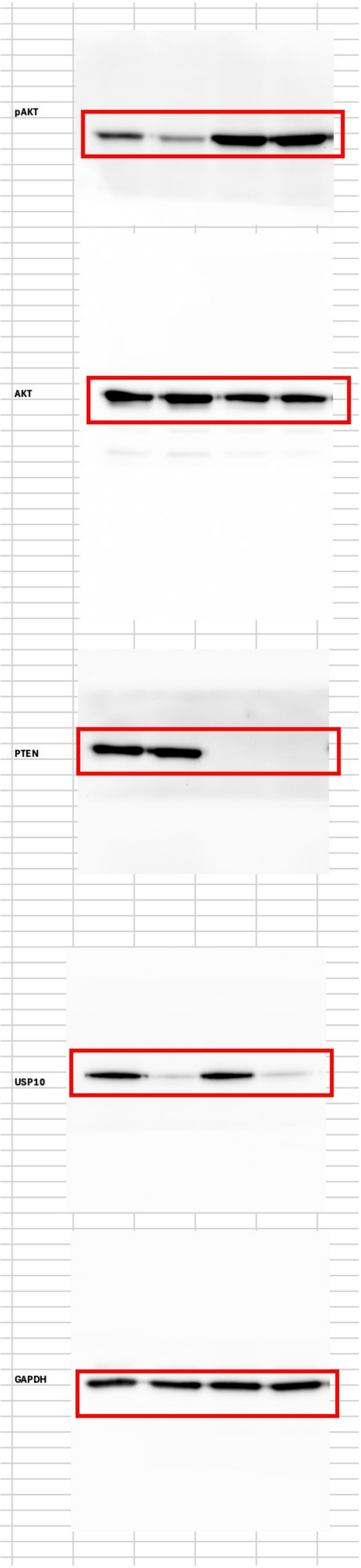

Full unedited gel for Supplementary Figure 11A

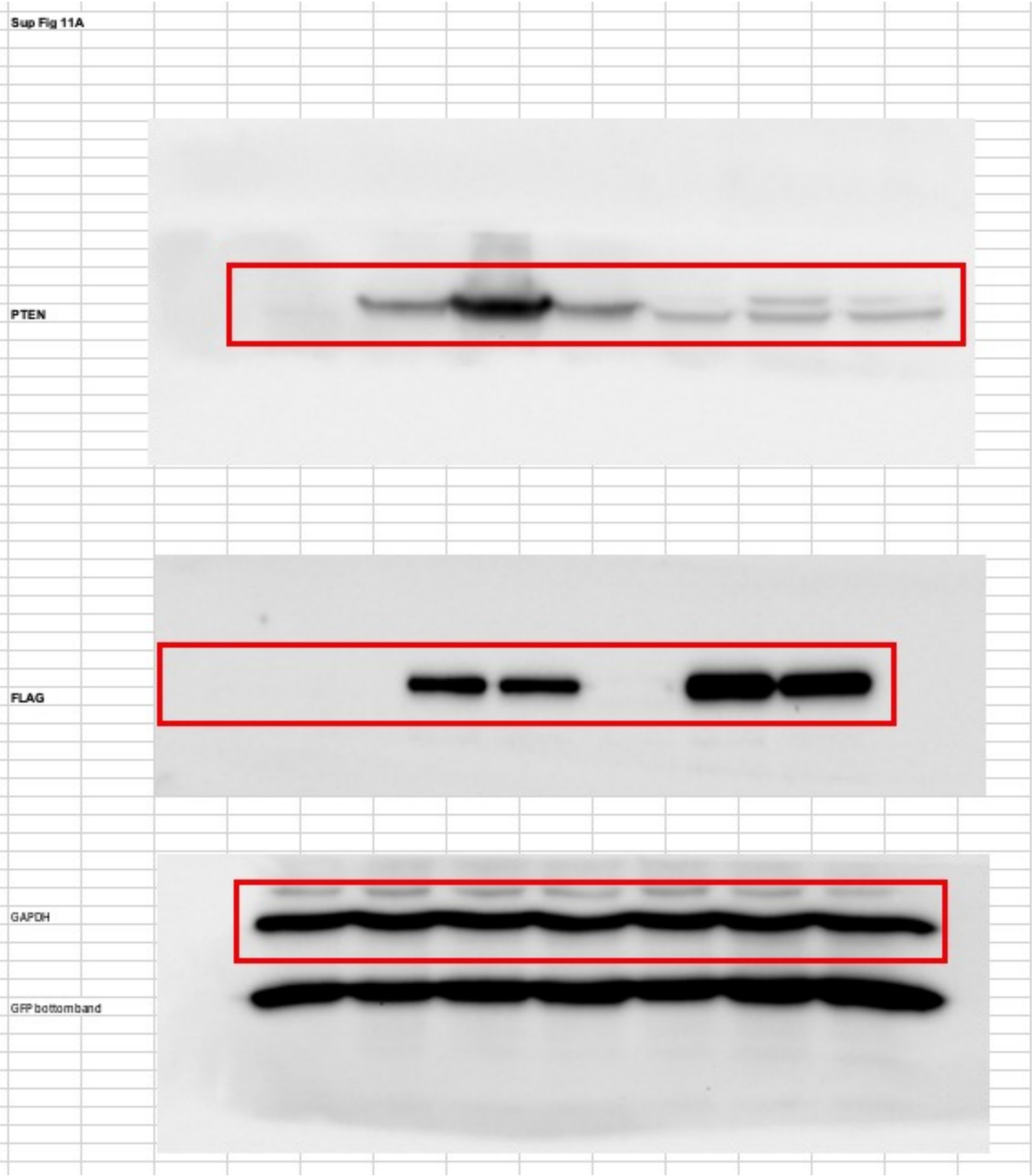

Full unedited gel for Supplementary Figure 11B

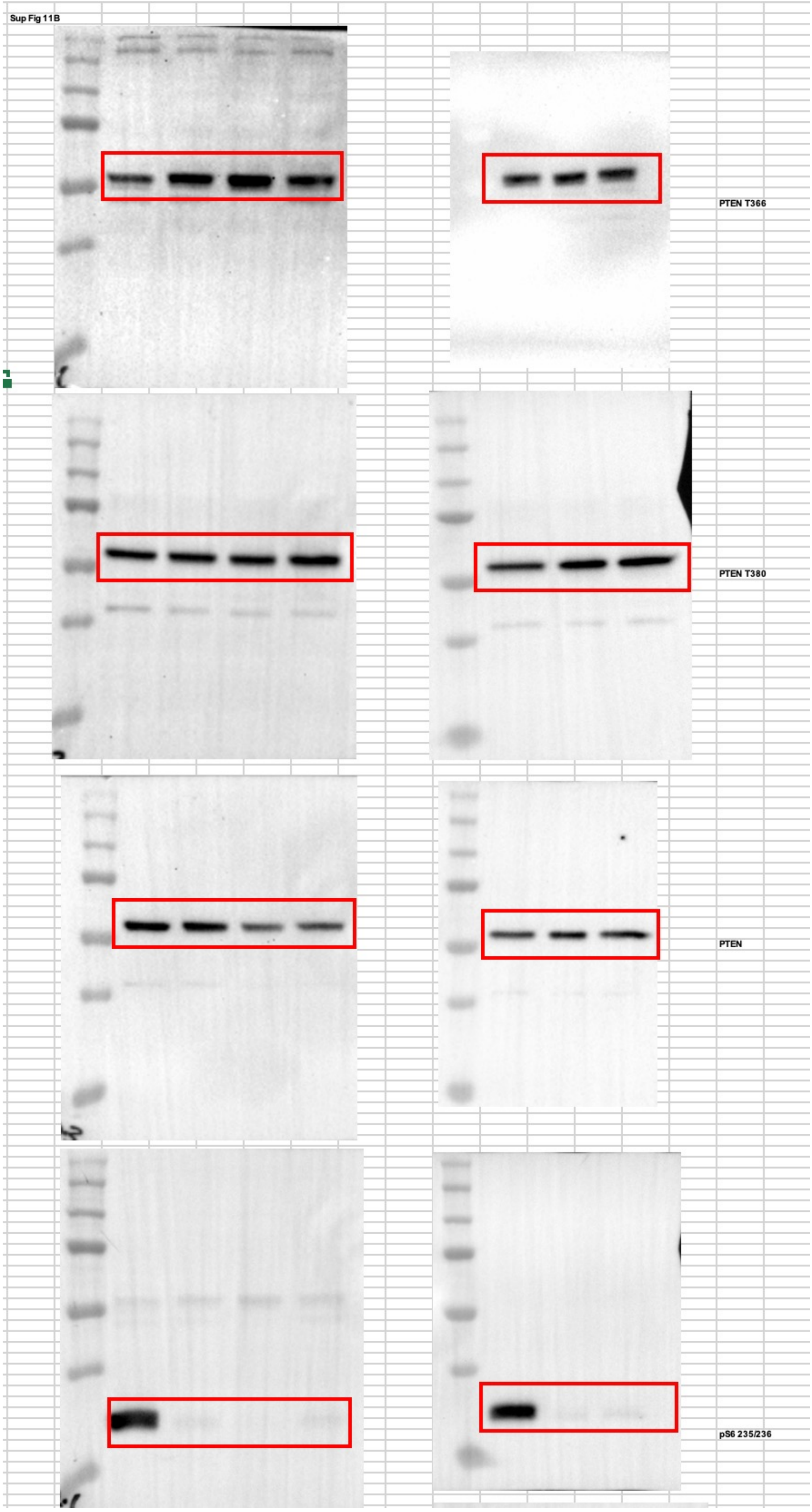

Full unedited gel for Supplementary Figure 11B

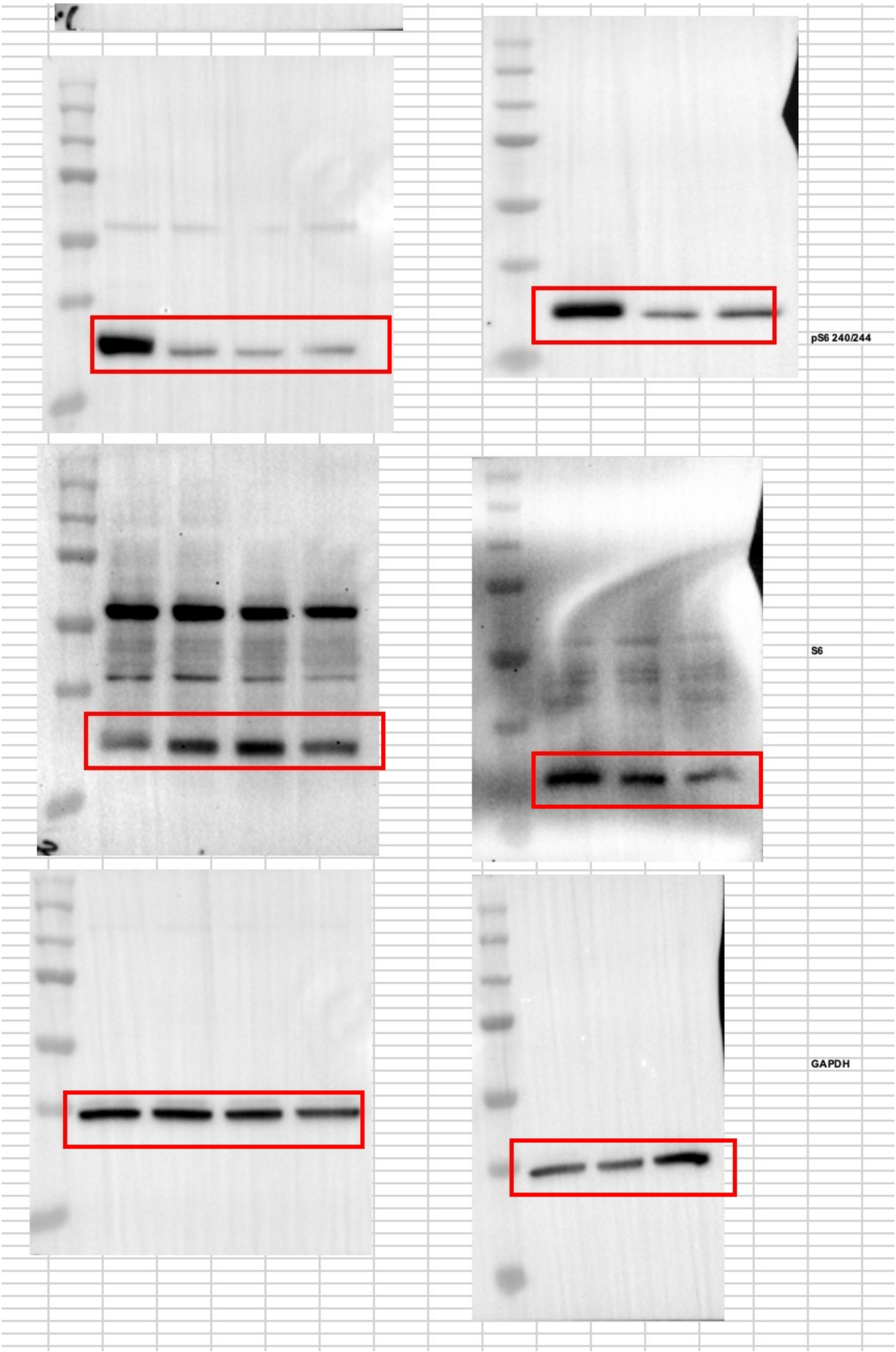

Full unedited gel for Supplementary Figure 11C

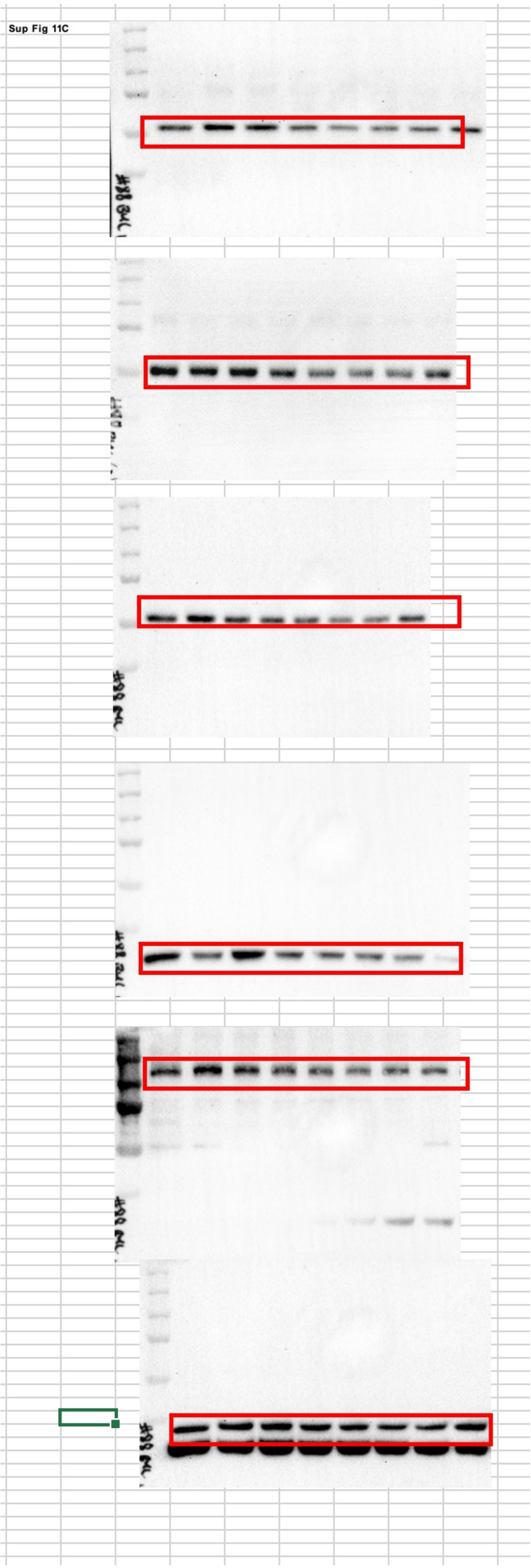

Full unedited gel for Supplementary Figure 11D

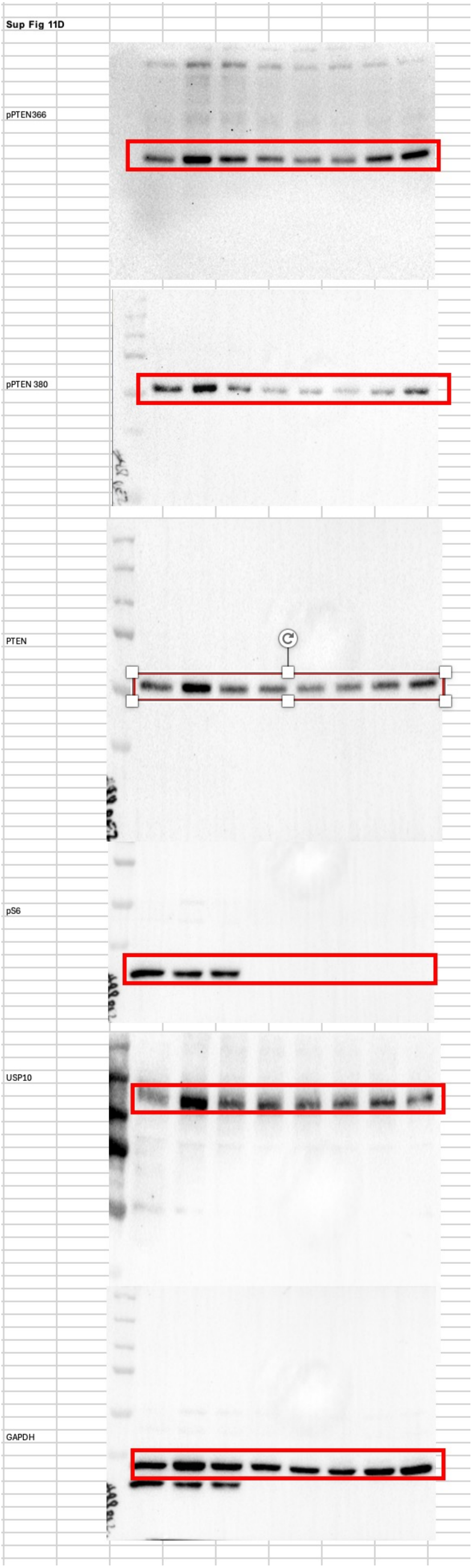

Full unedited gel for Supplementary Figure 11E

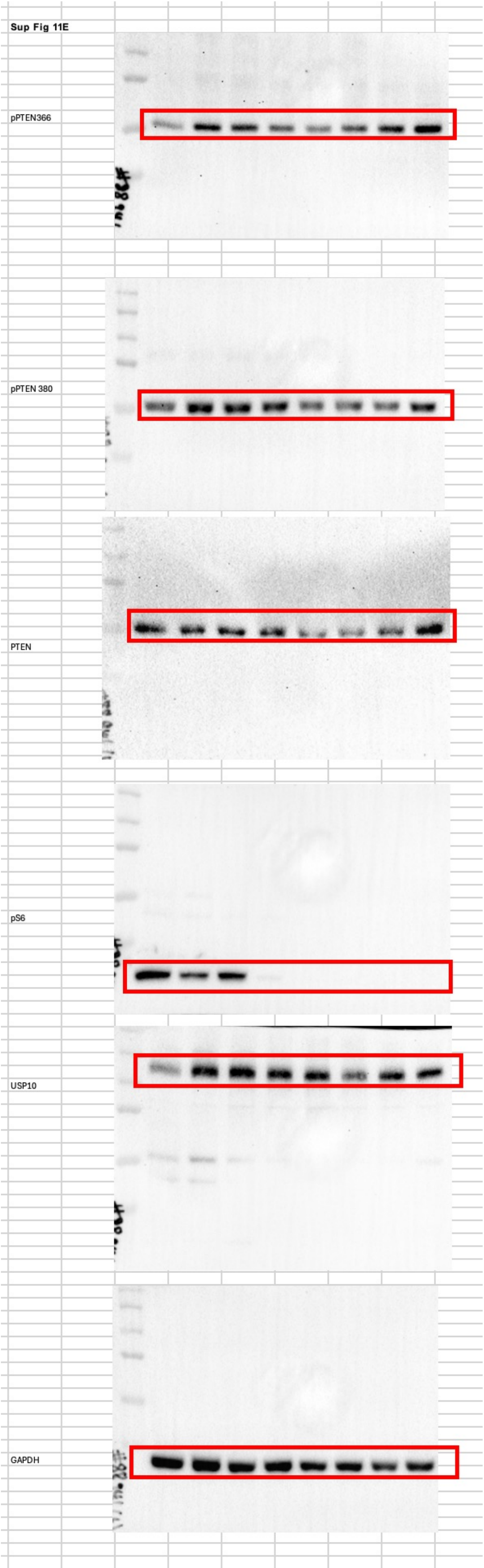

Full unedited gel for Supplementary Figure 11F

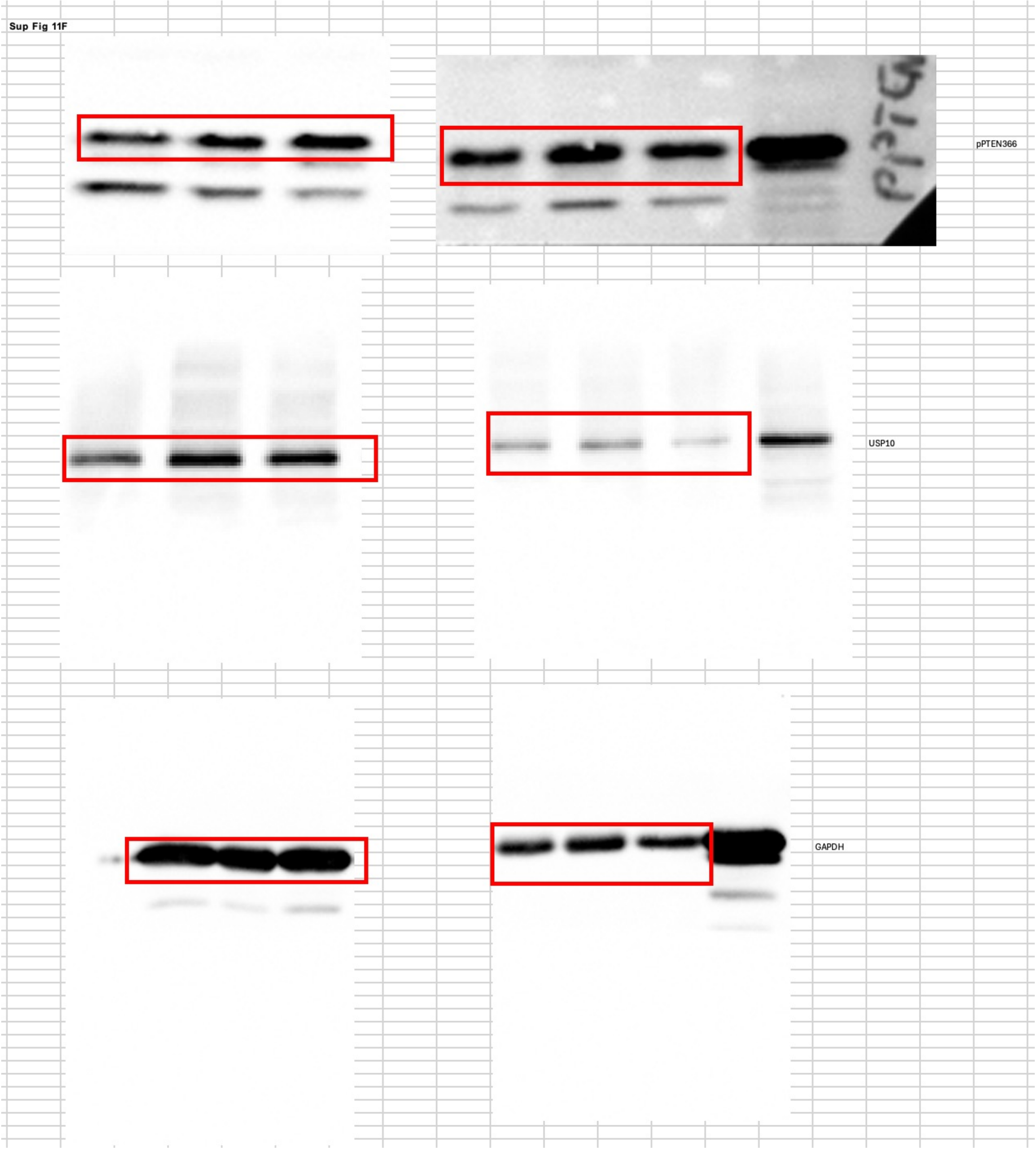

Full unedited gel for Supplementary Figure 12A

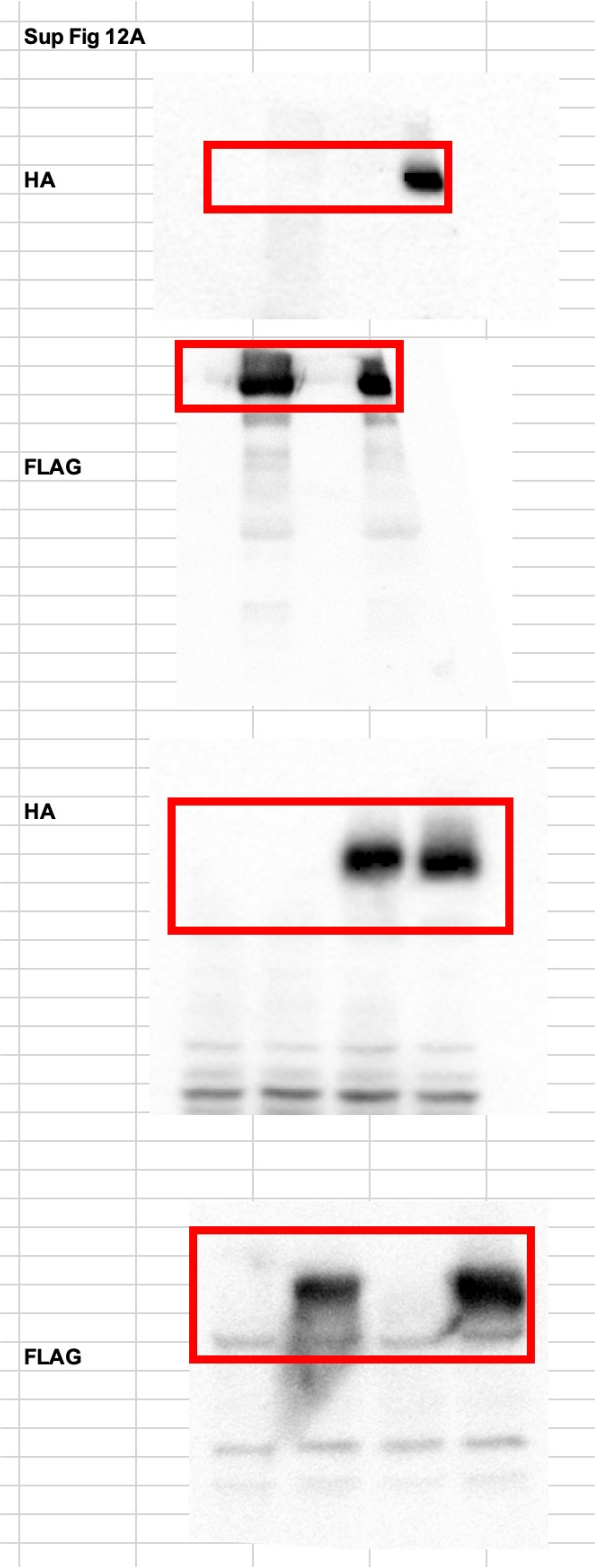

Full unedited gel for Supplementary Figure 12B

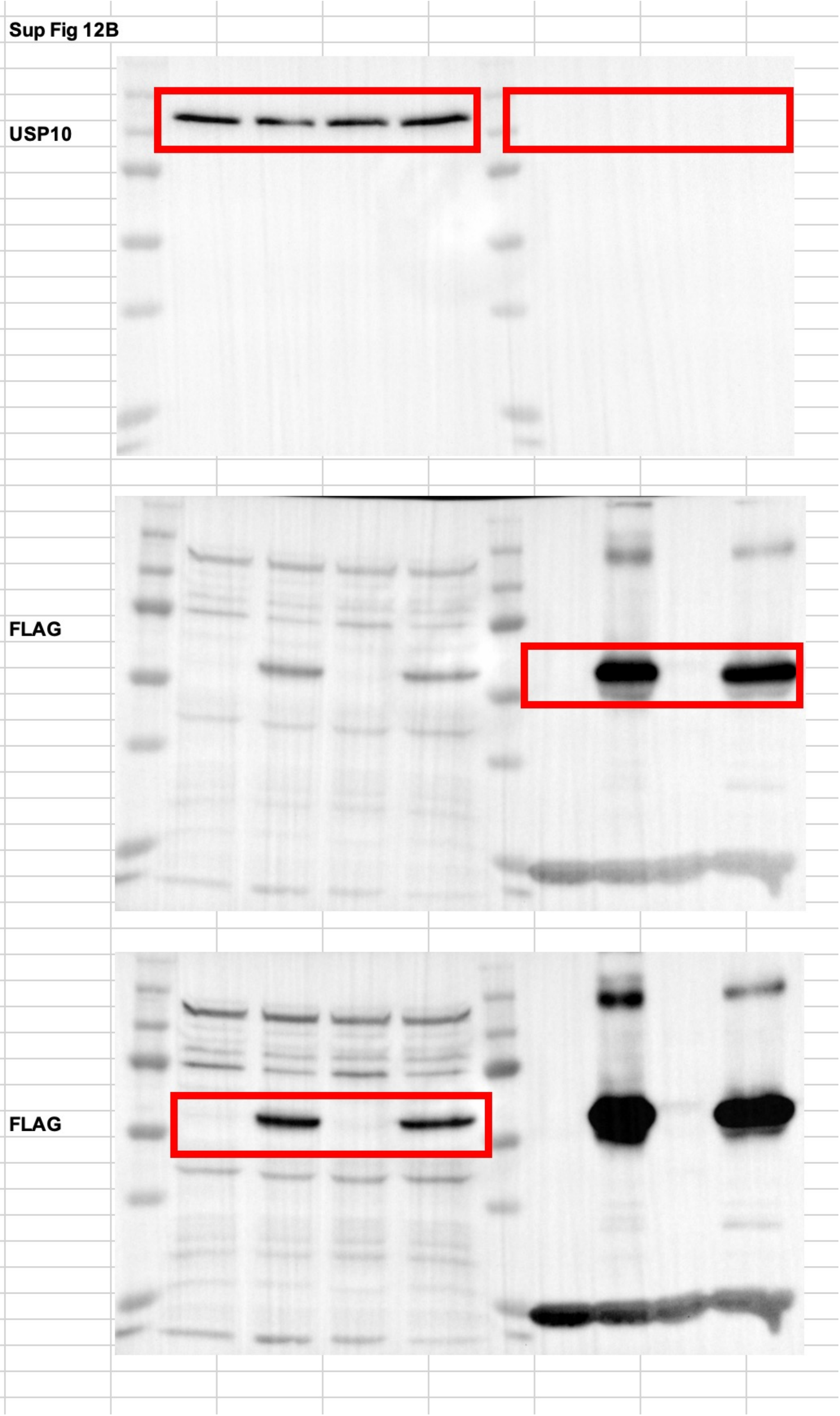

Full unedited gel for Supplementary Figure 12C

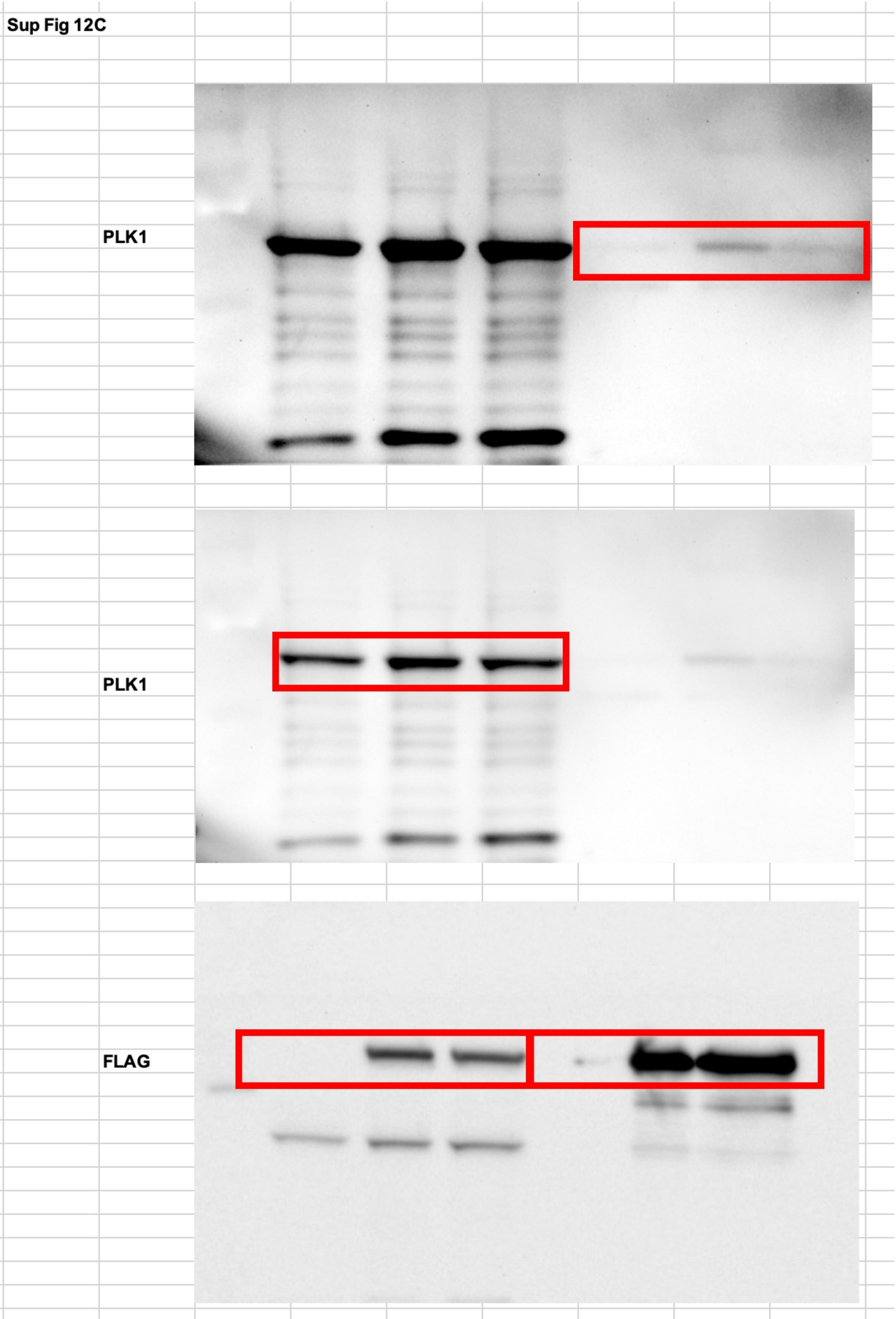

Full unedited gel for Supplementary Figure 12E

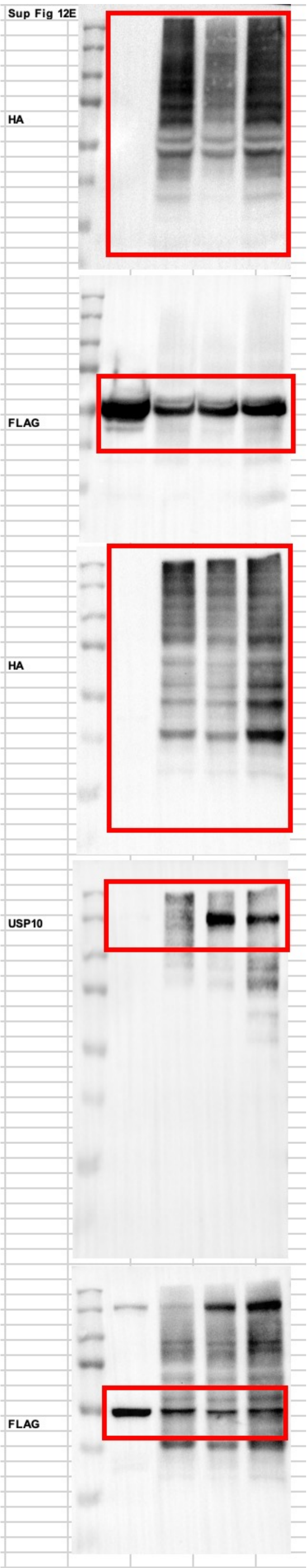

Full unedited gel for Supplementary Figure 13A

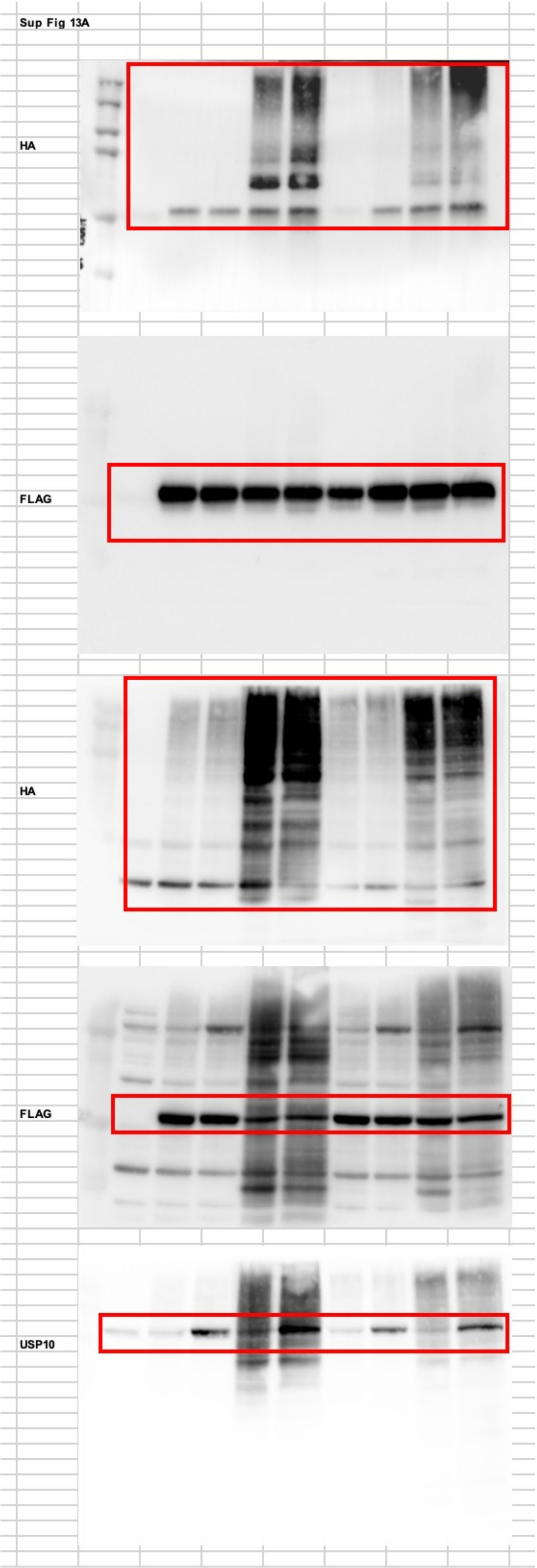

Full unedited gel for Supplementary Figure 13B

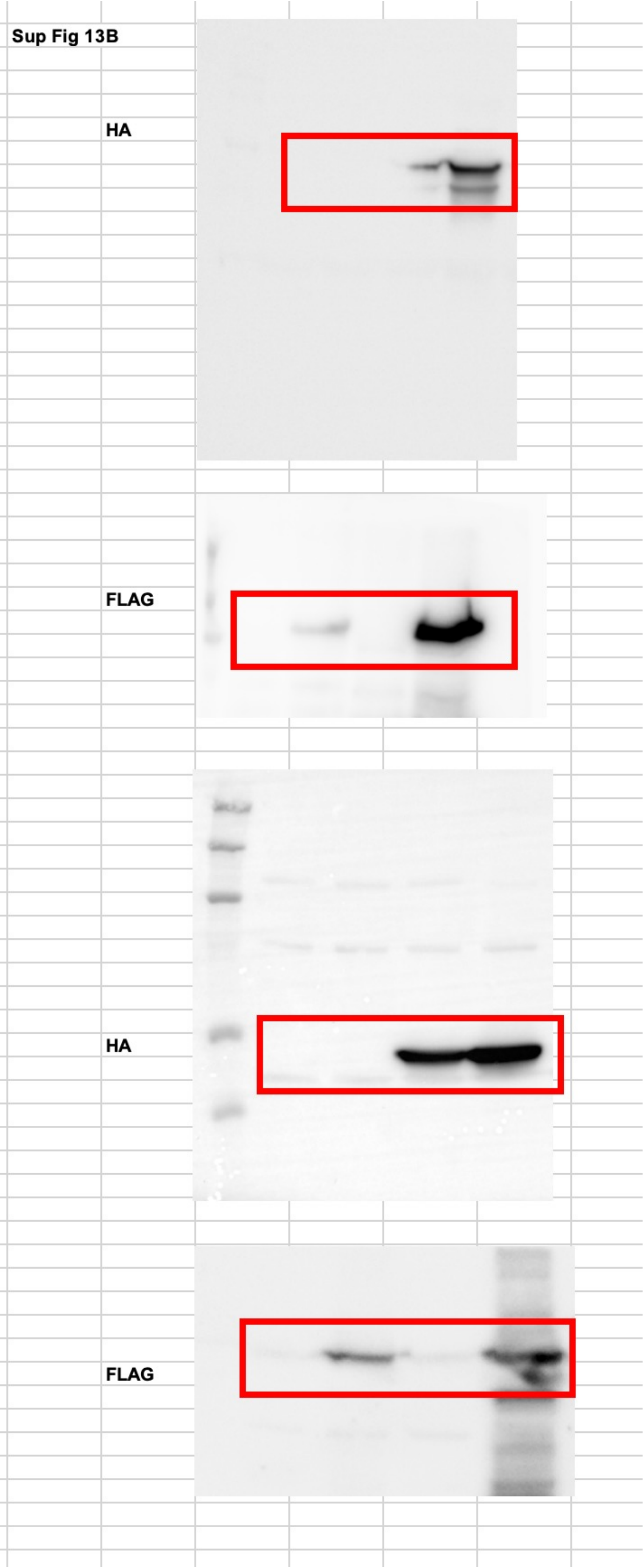

Full unedited gel for Supplementary Figure 13C

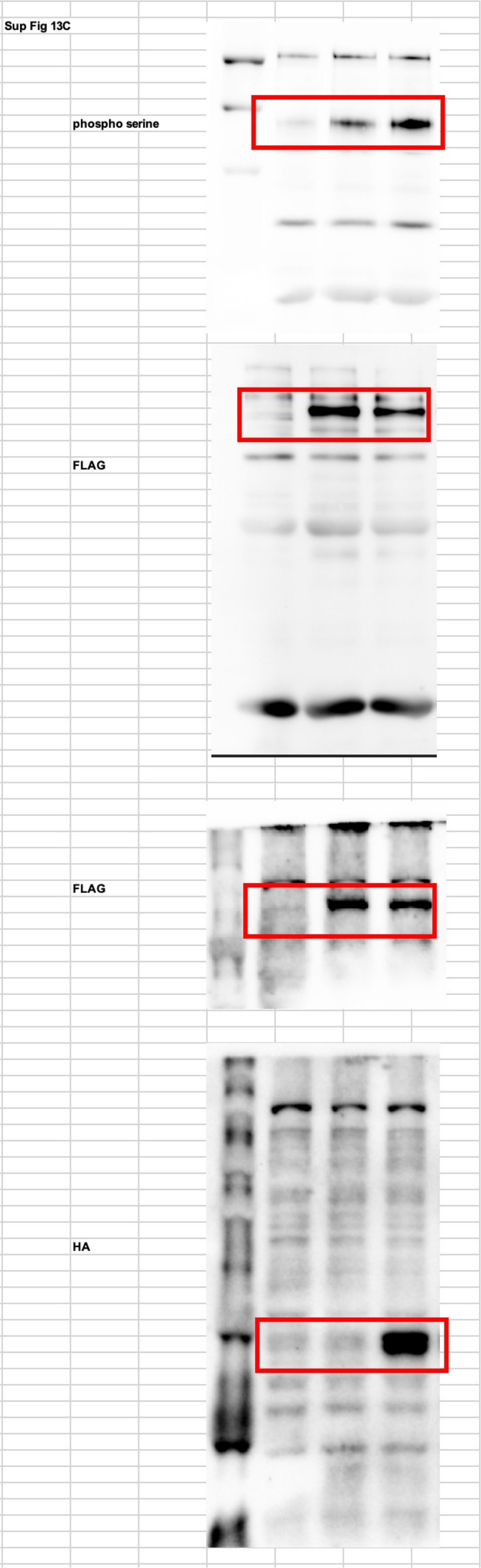

Full unedited gel for Supplementary Figure 13D

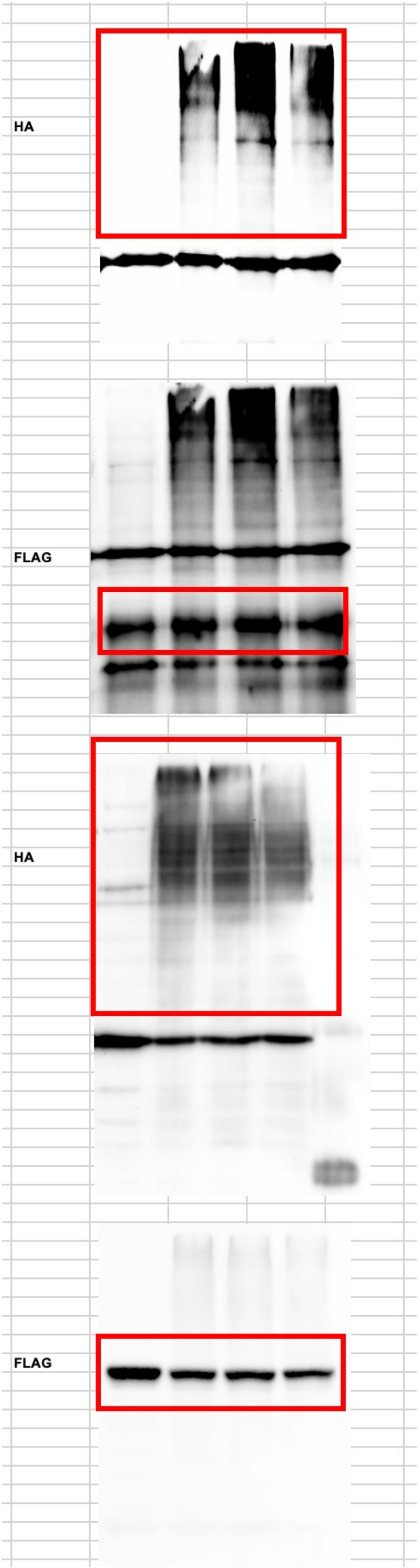

Full unedited gel for Supplementary Figure 15C

Sup Fig 15C

USP10

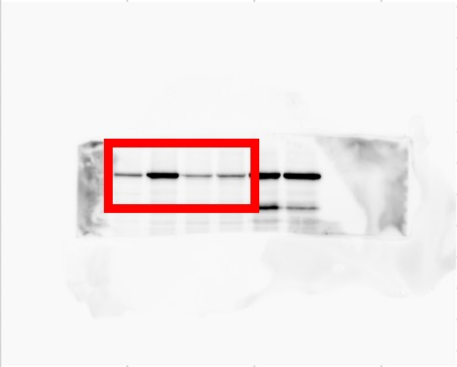

GAPDH

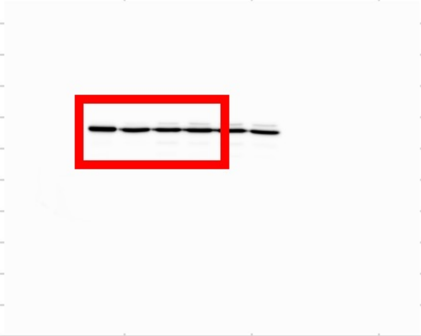

Full unedited gel for Supplementary Figure 15E

Sup Fig 15E

USP10

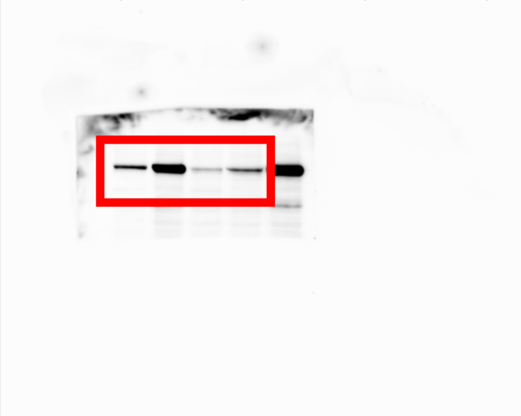

GAPDH

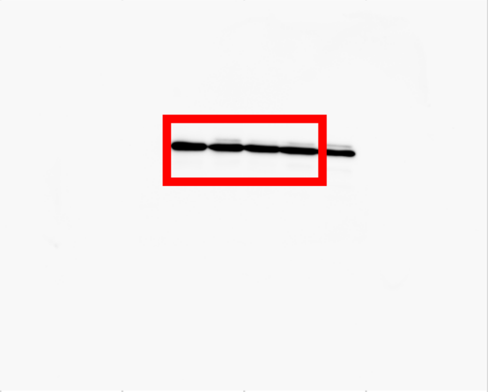

Full unedited gel for Figure 16F

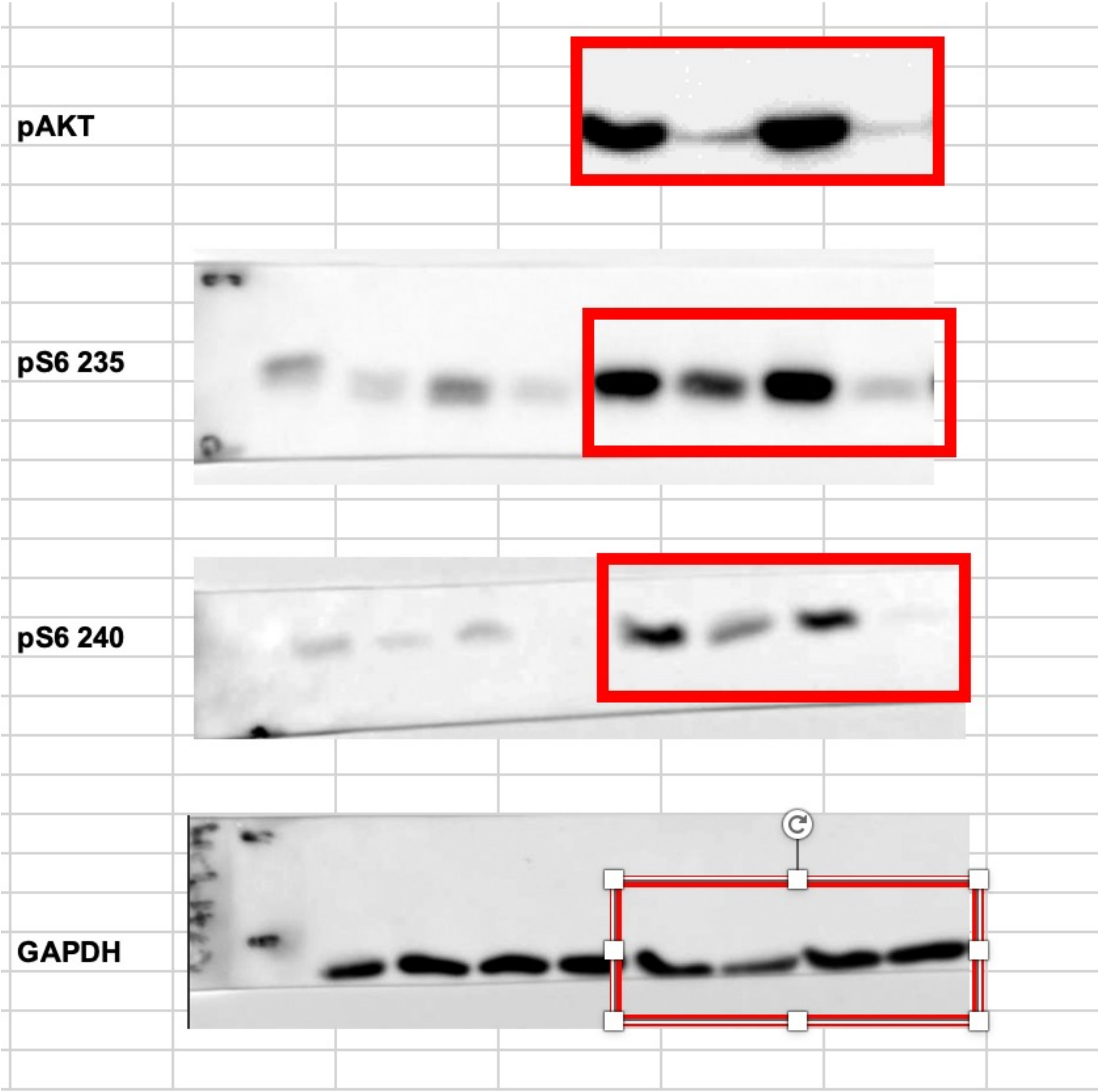

Full unedited gel for Supplementary Figure 17A

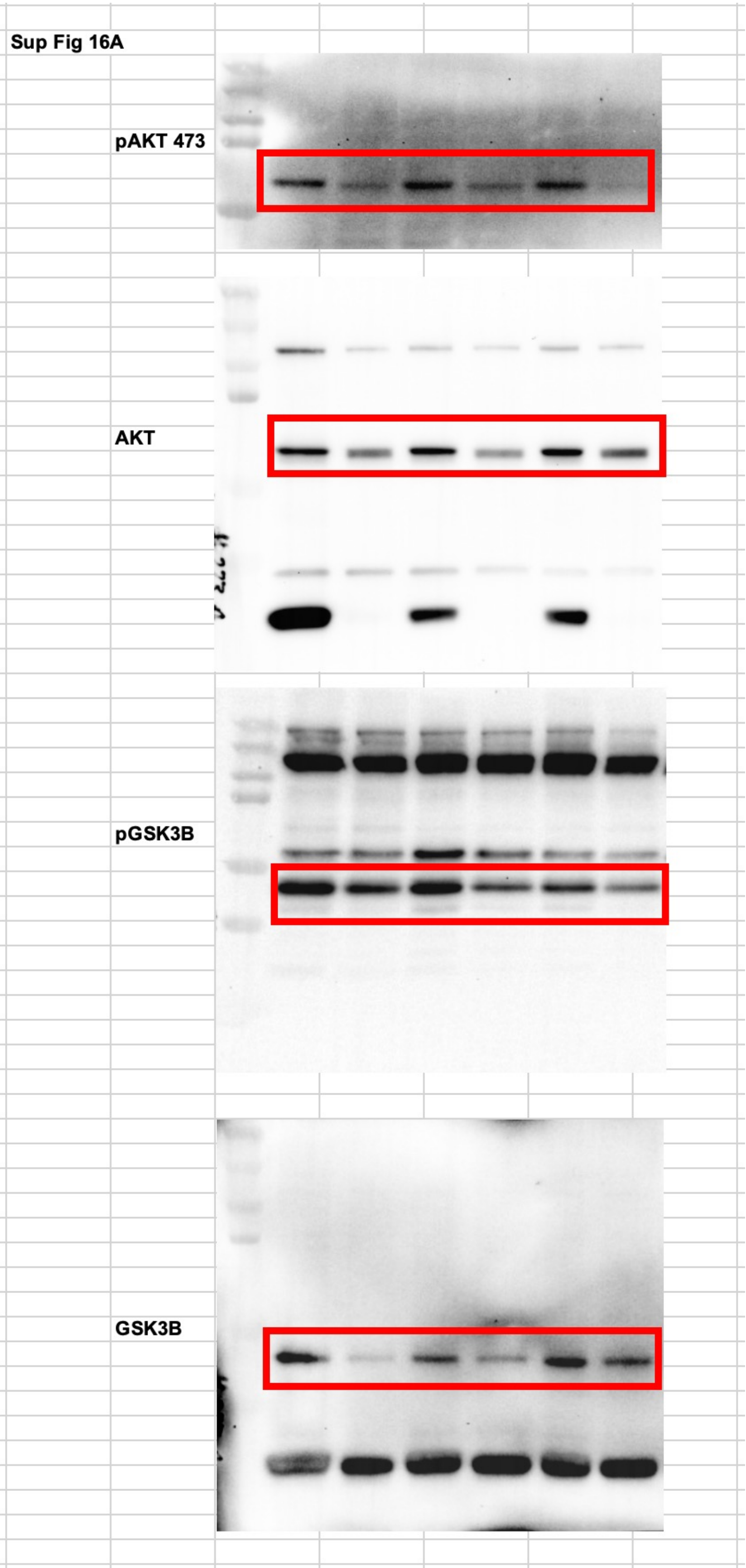

Full unedited gel for Supplementary Figure 17A

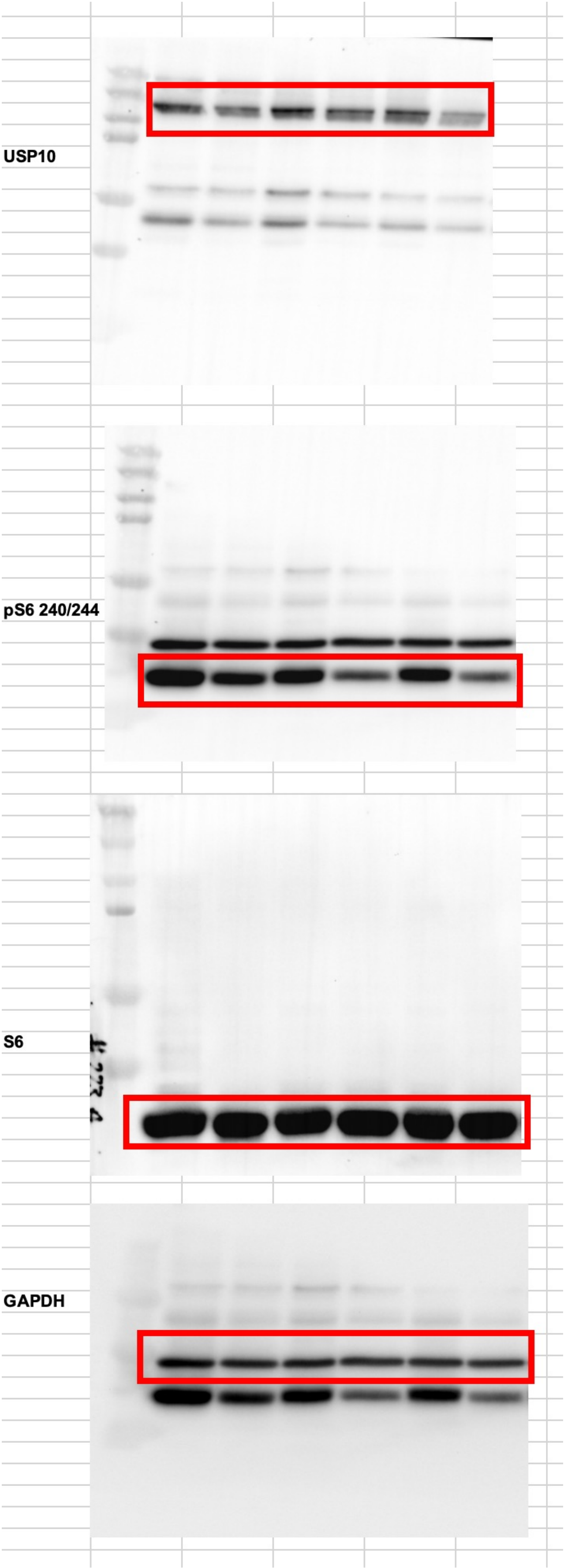

Full unedited gel for Supplementary Figure 17B

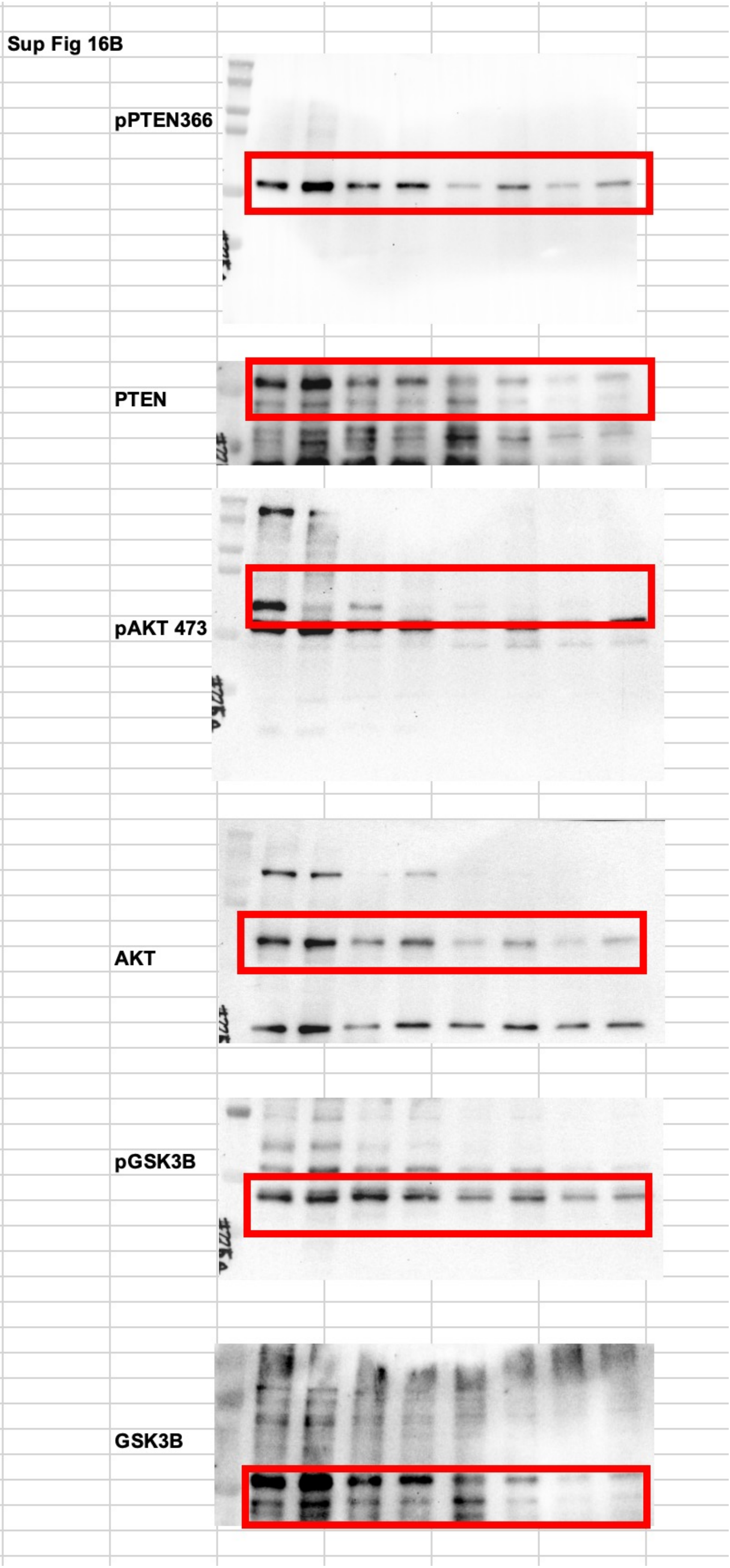

Full unedited gel for Supplementary Figure 17B

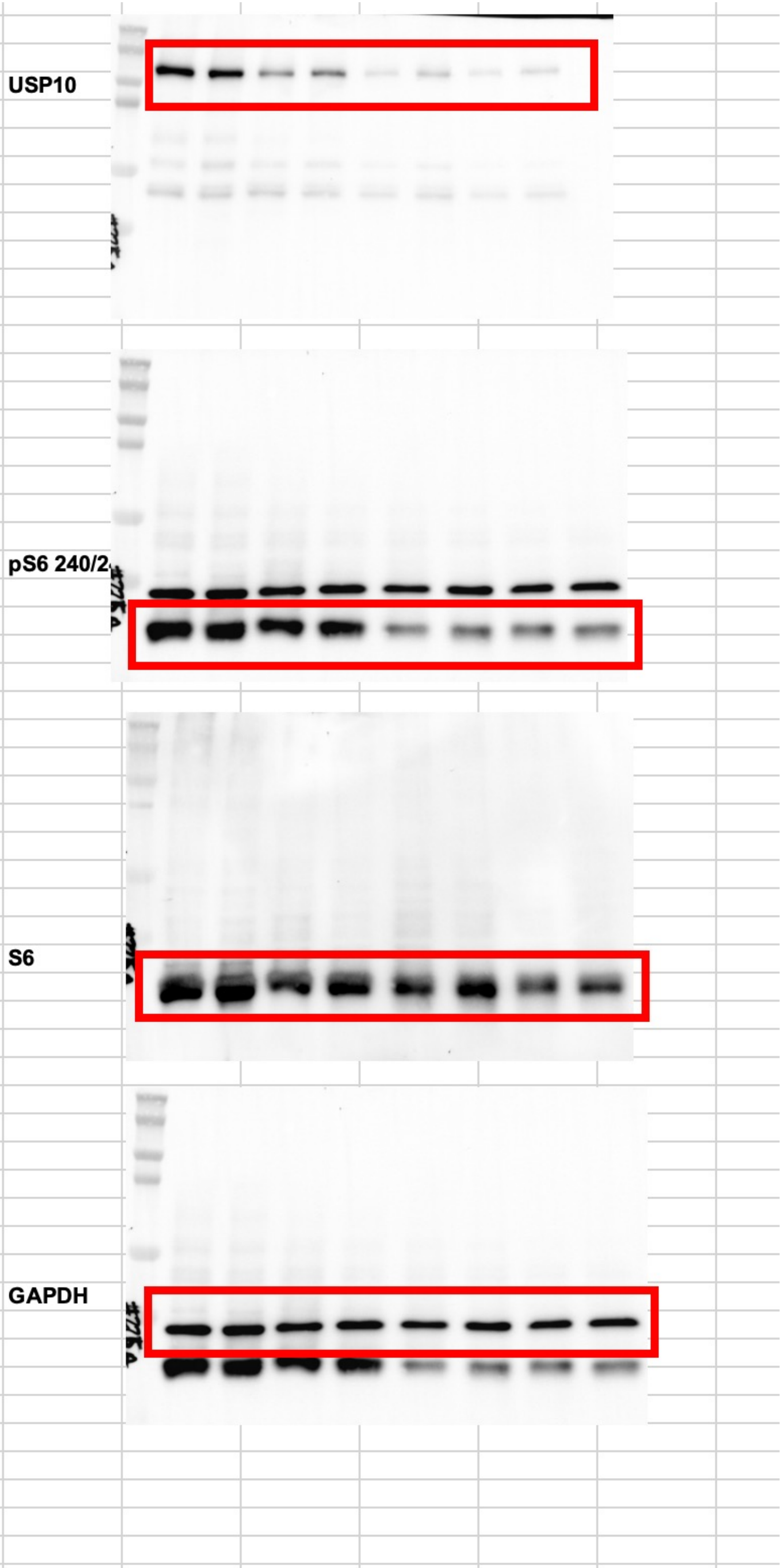

Full unedited gel for Supplementary Figure 18B

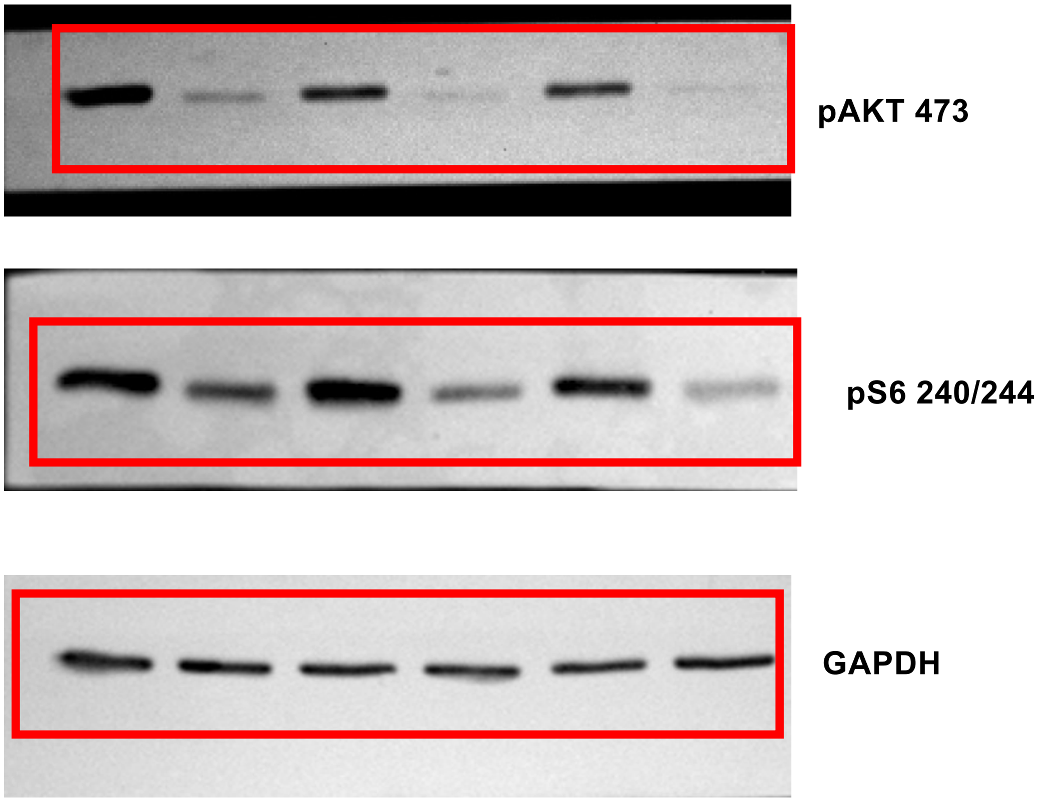

Supplement: Unedited blot and gel images [file jci-135-180927-s158.pdf]
